# Supplementary material for: Precision targeting of β-catenin induces tumor reprogramming and immunity in hepatocellular cancers
Source: Nat Commun. 2025 May 30;16:5009. doi: 10.1038/s41467-025-60457-2 (PMC12122713; doi:10.1038/s41467-025-60457-2)
Supplement: Supplementary file 1 — Supplementary Information [file 41467_2025_60457_MOESM1_ESM.pdf]

Supplementary Information

Precision targeting of β-catenin induces tumor reprogramming and immunity in hepatocellular cancers

Brandon M. Lehrich[1,2,3,4], Evan R. Delgado[3,5], Tyler M. Yasaka[1,2,3,4], Silvia Liu[1,2,3], Catherine Cao[1], Yuqing Liu[1], Mohammad N. Taheri[3,5,6], Xiangnan Guan[7], Hartmut Koeppen[7], Sucha Singh[1,2], Vik Meadows[1,2,3], Jia-Jun Liu[1,2,3], Anya Singh-Varma[1], Yekaterina Krutsenko[1], Minakshi Poddar[1,2], T. Kevin Hitchens[8], Lesley M. Foley[8], Binyong Liang[9], Alex Rialdi[10], Ravi P. Rai[1,2,3], Panari Patel[1,2], Madeline Riley[1,2], Aaron Bell[1,2,3], Reben Raeman[1,2,3], Tulin Dadali[11], Jason J. Luke[12], Ernesto Guccione[10], Mo R. Ebrahimkhani[3,5,6], Amaia Lujambio[10], Xin Chen[13], Martin Maier[11], Yulei Wang[7], Wendy Broom[11], *Junyan Tao[1,2,3], *Satdarshan P. Monga[1,2,3,14]

Affilitations

[1]Organ Pathobiology and Therapeutics Institute, University of Pittsburgh School of Medicine, Pittsburgh, PA, USA.
[2]Department of Pharmacology and Chemical Biology, University of Pittsburgh School of Medicine, Pittsburgh, PA, USA.
[3]Pittsburgh Liver Research Center, University of Pittsburgh and University of Pittsburgh Medical Center, Pittsburgh, PA, USA.
[4]Medical Scientist Training Program, University of Pittsburgh, Pittsburgh, Pennsylvania, USA.
[5]Department of Pathology, Division of Experimental Pathology, University of Pittsburgh School of Medicine, Pittsburgh, PA, USA.
[6]Department of Bioengineering, Swanson School of Engineering, University of Pittsburgh, Pittsburgh, PA, USA.
[7]Translational Medicine, Genentech Inc., San Francisco, CA, USA.
[8]University of Pittsburgh School of Medicine, Pittsburgh, Pennsylvania, USA.
[9]Hepatic Surgery Center, Department of Surgery, Tongji Hospital, Tongji Medical College, Huazhong University of Science and Technology, Wuhan, China.
[10]Department of Oncological Sciences, Icahn School of Medicine at Mount Sinai, New York, NY, USA.
[11]Alnylam Pharmaceuticals, Boston, MA, USA.
[12]UPMC Hillman Cancer Center and University of Pittsburgh, Pittsburgh, PA, USA.
[13]Cancer Biology Program, University of Hawaii Cancer Center, Honolulu, HI, USA.
[14]Division of Gastroenterology, Hepatology and Nutrition, Department of Medicine, University of Pittsburgh School of Medicine, Pittsburgh, PA, USA.
*These authors jointly supervised this work.

Table of Contents (page #'s)

1. **Title pages: 1-2**
2. **Supplemental Figures: 3-74**
3. **Supplemental Tables: 75-85**

SUPPLEMENTAL FIGURES

Supplementary Figure 1

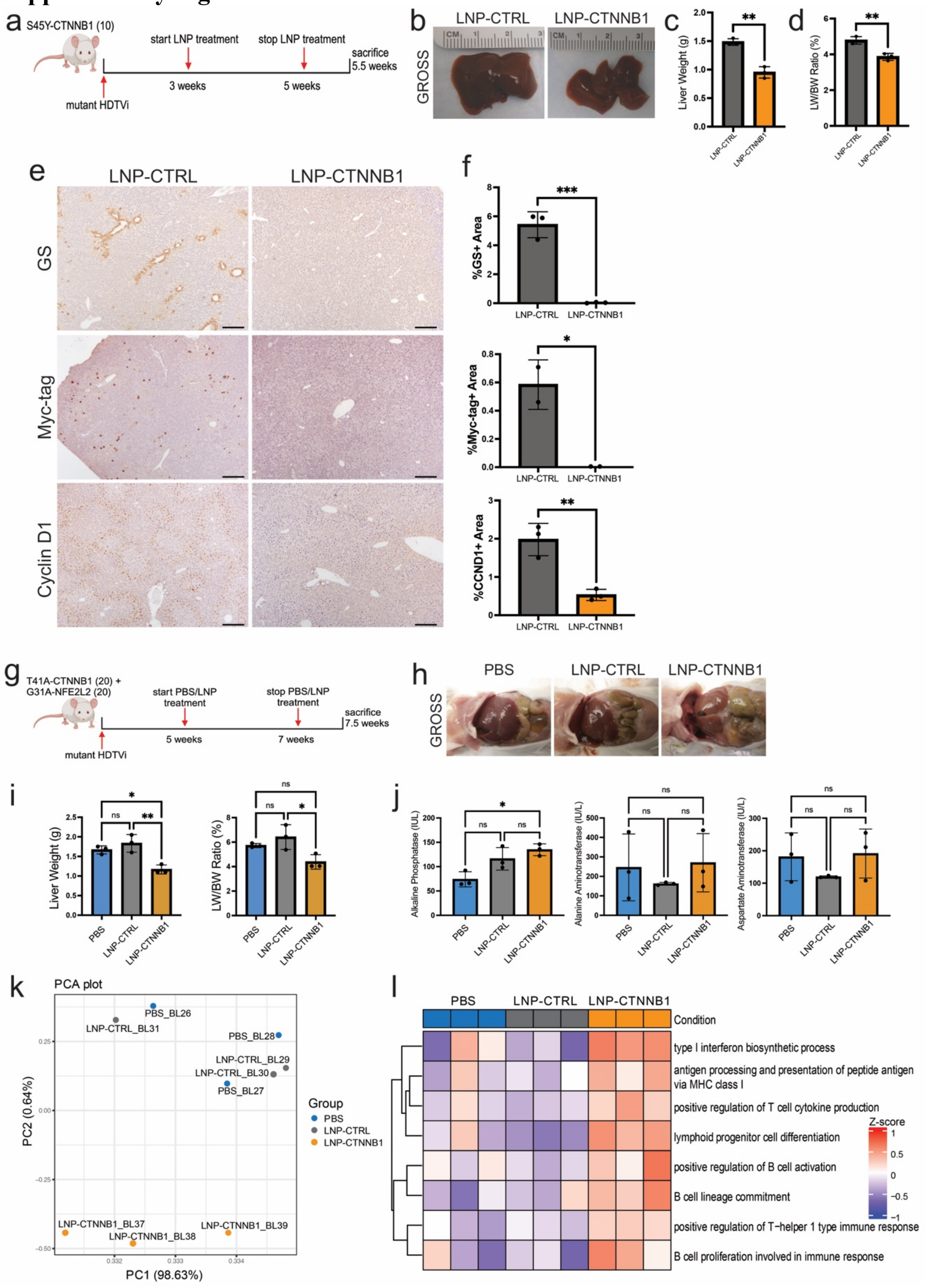

Supplementary Figure 1. RNAi-mediated β-catenin inhibition results in potent and specific *hCTNNB1* and *mCtnnb1* knockdown *in vivo* (related to Figure 1).

(a) LNP treatment scheme with overexpression of S45Y-mutant-β-catenin. Mice were given twice weekly injections at 3mg/kg over two weeks and then sacrificed 5.5-weeks post-hydrodynamic tail vein injection (HDTVi). Created in BioRender. Lehrich, B. (2025) https://BioRender.com/f5xqizn.

(b) Representative gross liver images of LNP-CTRL and LNP-CTNNB1 (3mg/kg) treated mice with overexpression of S45Y-mutant-β-catenin at 5.5-week timepoint.

(c) Liver weights comparing LNP-CTRL (n=3) and LNP-CTNNB1 (n=3; 3mg/kg) treated mice with overexpression of S45Y-mutant-β-catenin at 5.5-week timepoint. $^{**}p$=0.0013 calculated by unpaired two-tailed Student's t-test. Data presented as mean with error bars representing standard deviation (SD).

(d) Liver weight/body weight (LW/BW) ratio comparing LNP-CTRL (n=3) and LNP-CTNNB1 (n=3; 3mg/kg) treated mice with overexpression of S45Y-mutant-β-catenin at 5.5-week timepoint. $^{**}p$=0.0053 calculated by unpaired two-tailed Student's t-test. Data presented as mean with error bars representing SD.

(e) Representative immunohistochemistry (IHC) images from LNP-CTRL and LNP-CTNNB1 (3mg/kg) treated mice with overexpression of S45Y-mutant-β-catenin at 5.5-week timepoint for glutamine synthetase (GS), Cyclin D1, and Myc-tag. 5X objective magnification for the images. Scale bar indicates 200μm.

(f) Quantification of the IHC images from (e) comparing expression of GS, Cyclin D1, and Myc-tag between LNP-CTRL and LNP-CTNNB1 (3mg/kg) treated animals. Quantification performed from n=2-3 biological replicates. $^{***}p$=0.0005, $^{*}p$=0.0429, $^{**}p$=0.005 for GS, Cyclin D1, and Myc-tag, respectively, calculated by unpaired two-tailed Student's t-test. Data presented as mean with error bars representing SD.

(g) LNP treatment scheme with overexpression of T41A-mutant-β-catenin + G31A-NFE2L2. Mice were given twice weekly injections at 3mg/kg over two weeks and then sacrificed 7.5-weeks post-HDTVi. Created in BioRender. Lehrich, B. (2025) https://BioRender.com/xq3f0t6.

(h) Representative gross livers of PBS, LNP-CTRL, and LNP-CTNNB1 (3mg/kg) treated mice with overexpression of T41A-mutant-β-catenin + G31A-NFE2L2 at 7.5-week timepoint.

(i) (Left) Liver weights and (Right) LW/BW comparing PBS (n=3), LNP-CTRL (n=3), and LNP-CTNNB1 (n=3; 3mg/kg) with overexpression of T41A-mutant-β-catenin + G31A-NFE2L2 at 7.5-week timepoint. Data presented as mean with error bars representing SD. P-values calculated by one-way ANOVA with Tukey-HSD post-hoc correction. Ns, not significant. Exact p-values in source data file.

(j) Liver serum function tests of alkaline phosphatase, alanine aminotransferase, and aspartate aminotransferase comparing PBS (n=3), LNP-CTRL (n=3), and LNP-CTNNB1 (n=3; 3mg/kg) treated mice with overexpression of T41A-mutant-β-catenin + G31A-NFE2L2 at 7.5-week timepoint. Data presented as mean with error bars representing SD. P-values calculated by one-way ANOVA with Tukey-HSD post-hoc correction. Ns, not significant. Exact p-values in source data file.

(k) PCA of bulk RNA-seq of β-catenin-Nrf2 (β-N) model each treated with PBS (n=3), LNP-CTRL (n=3), and LNP-CTNNB1 (n=3; 3mg/kg).

(l) Gene set variation analysis for gene ontology immune-related pathways for β-N model treated with either PBS (n=3), LNP-CTRL (n=3), and LNP-CTNNB1 (n=3; 3mg/kg).

Supplementary Figure 2

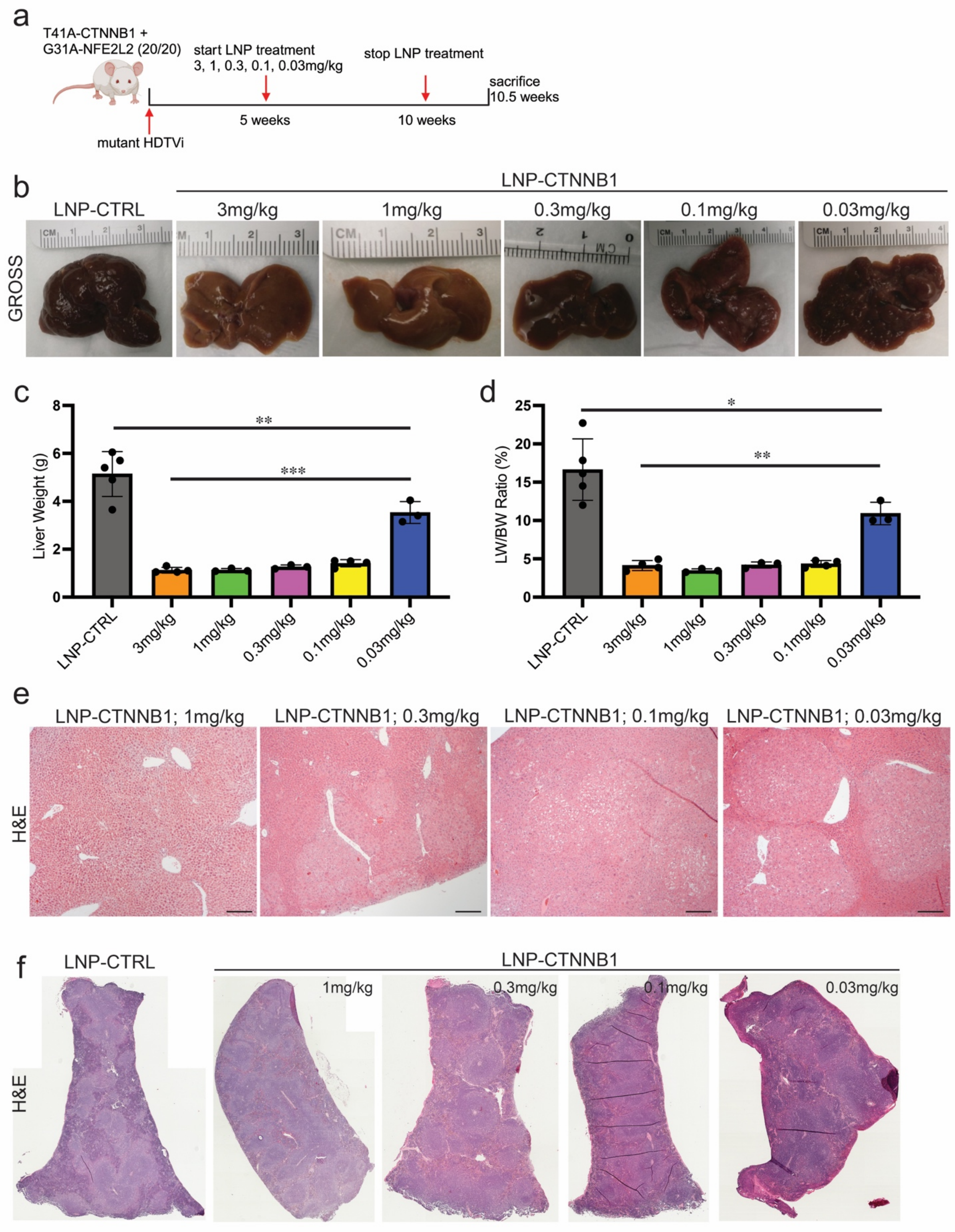

Supplementary Figure 2. RNAi-mediated β-catenin inhibition is efficacious at 1mg/kg dosage in β-catenin-Nrf2 model in early-stage disease (related to Figure 1).

(a) LNP treatment scheme in β-catenin-Nrf2 (β-N) model. Mice received weekly intravenous (I.V.) injections of LNP-CTNNB1 at 3mg/kg, 1mg/kg, 0.3mg/kg, 0.1mg/kg, and 0.03mg/kg dosages starting at 5-weeks post-hydrodynamic tail vein injection (HDTVi). Created in BioRender. Lehrich, B. (2025) https://BioRender.com/hjch5c3.

(b) Representative gross liver images of LNP-CTRL and LNP-CTNNB1 (at each dosage: 3mg/kg, 1mg/kg, 0.3mg/kg, 0.1mg/kg, and 0.03mg/kg) treated β-N animals at 10.5-week timepoint.

(c) Liver weights comparing LNP-CTRL (n=5), 3mg/kg LNP-CTNNB1 (n=4), 1mg/kg LNP-CTNNB1 (n=3), 0.3mg/kg LNP-CTNNB1 (n=3), 0.1m/kg LNP-CTNNB1 (n=4), 0.03mg/kg LNP-CTNNB1 (n=3) treated β-N animals at 10.5-week timepoint. Data presented as mean with error bars representing standard deviation (SD). P-values calculated by one-way ANOVA with Tukey-HSD post-hoc correction. Exact p-values in source data file.

(d) Liver weight/body weight (LW/BW) ratio comparing LNP-CTRL (n=5), 3mg/kg LNP-CTNNB1 (n=4), 1mg/kg LNP-CTNNB1 (n=3), 0.3mg/kg LNP-CTNNB1 (n=3), 0.1m/kg LNP-CTNNB1 (n=4), 0.03mg/kg LNP-CTNNB1 (n=3) treated β-N animals at 10.5-week timepoint. Data presented as mean with error bars representing SD. P-values calculated by one-way ANOVA with Tukey-HSD post-hoc correction. Exact p-values in source data file.

(e) Representative hematoxylin and eosin (H&E) staining comparing LNP-CTNNB1 at each dosage: 1mg/kg, 0.3mg/kg, 0.1mg/kg, and 0.03mg/kg in β-N animals at 10.5-week timepoint. 5X objective magnification for each of the images. Scale bar indicates 200μm.

(f) Representative tiled images of hematoxylin and eosin (H&E) staining of spleens comparing LNP-CTRL and LNP-CTNNB1 treated animals at each dosage: 1mg/kg, 0.3mg/kg, 0.1mg/kg, and 0.03mg/kg in β-N animals at 10.5-week timepoint. Scale bar indicates magnification.

Supplementary Figure 3

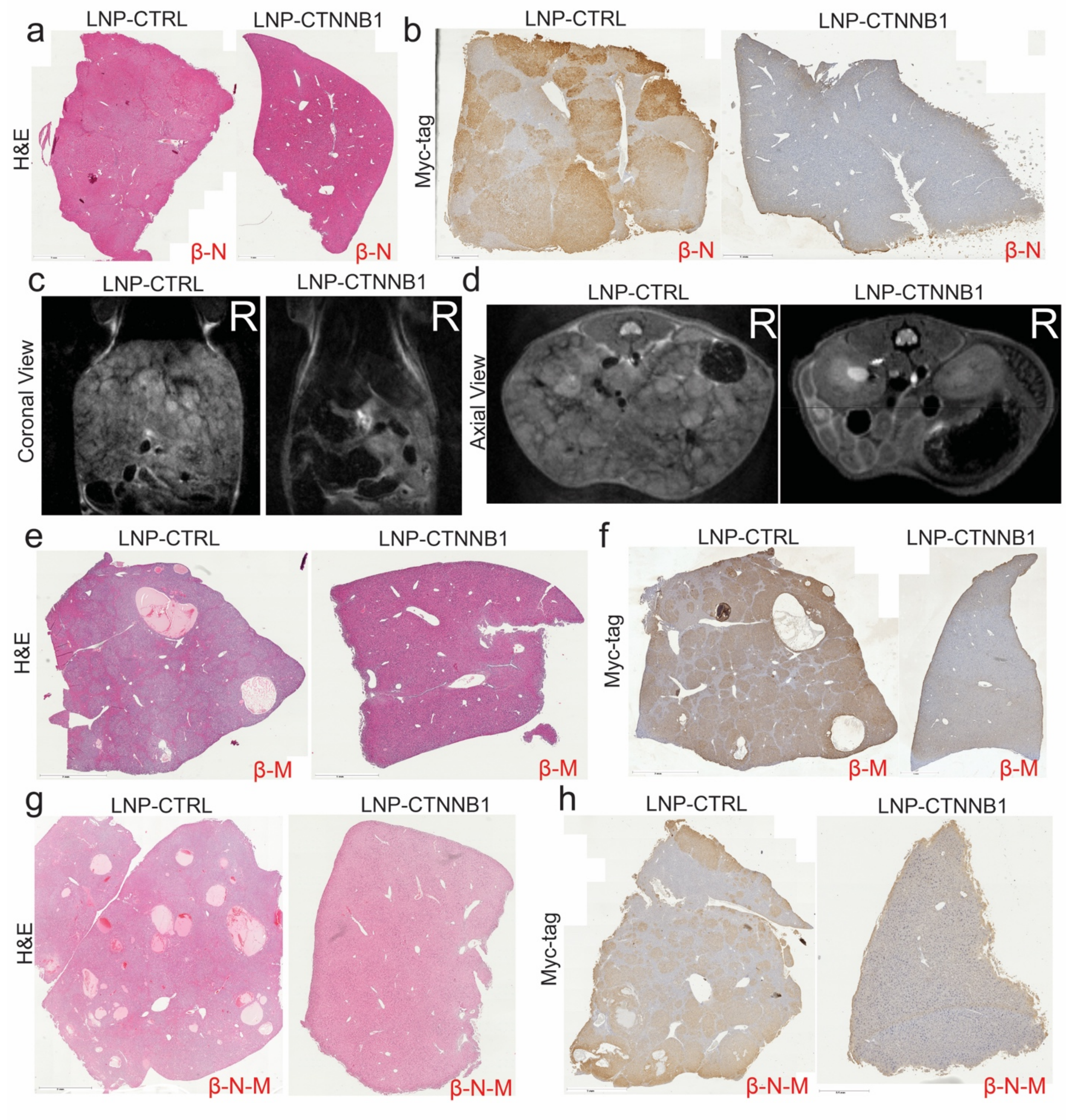

Supplementary Figure 3. RNAi-mediated β-catenin inhibition is efficacious in multiple *CTNNB1*-mutated mouse models in early-stage disease setting (related to Figure 1).

(a) Representative tiled images of hematoxylin and eosin (H&E) staining comparing LNP-CTRL and LNP-CTNNB1 (1mg/kg) treated β-catenin-Nrf2 (β-N) animals at 10.5-week timepoint. Scale bar indicates magnification.

(b) Representative tiled images of Myc-tag (present on the β-catenin plasmid) immunohistochemistry (IHC) comparing LNP-CTRL and LNP-CTNNB1 (1mg/kg) treated β-N animals at 10.5-week timepoint. Scale bar indicates magnification.

(c) Representative magnetic resonance image (MRI) coronal sections comparing LNP-CTRL and LNP-CTNNB1 (1mg/kg) treated β-N animals at 10.5-week timepoint.

(d) Representative MRI axial sections comparing LNP-CTRL and LNP-CTNNB1 (1mg/kg) treated β-N animals at 10.5-week timepoint.

(e) Representative tiled images of H&E staining comparing LNP-CTRL and LNP-CTNNB1 (1mg/kg) treated β-catenin-hMet (β-M) animals at 8.5-week timepoint. Scale bar indicates magnification.

(f) Representative tiled images of Myc-tag IHC comparing LNP-CTRL and LNP-CTNNB1 (1mg/kg) treated β-M animals at 8.5-week timepoint. Scale bar indicates magnification.

(g) Representative tiled images of H&E staining comparing LNP-CTRL and LNP-CTNNB1 (1mg/kg) treated β-catenin-Nrf2-hMet (β-N-M) animals at 7.5-week timepoint. Scale bar indicates magnification.

(h) Representative tiled images of Myc-tag IHC comparing LNP-CTRL and LNP-CTNNB1 (1mg/kg) treated β-N-M animals at 7.5-week timepoint. Scale bar indicates magnification.

Supplementary Figure 4

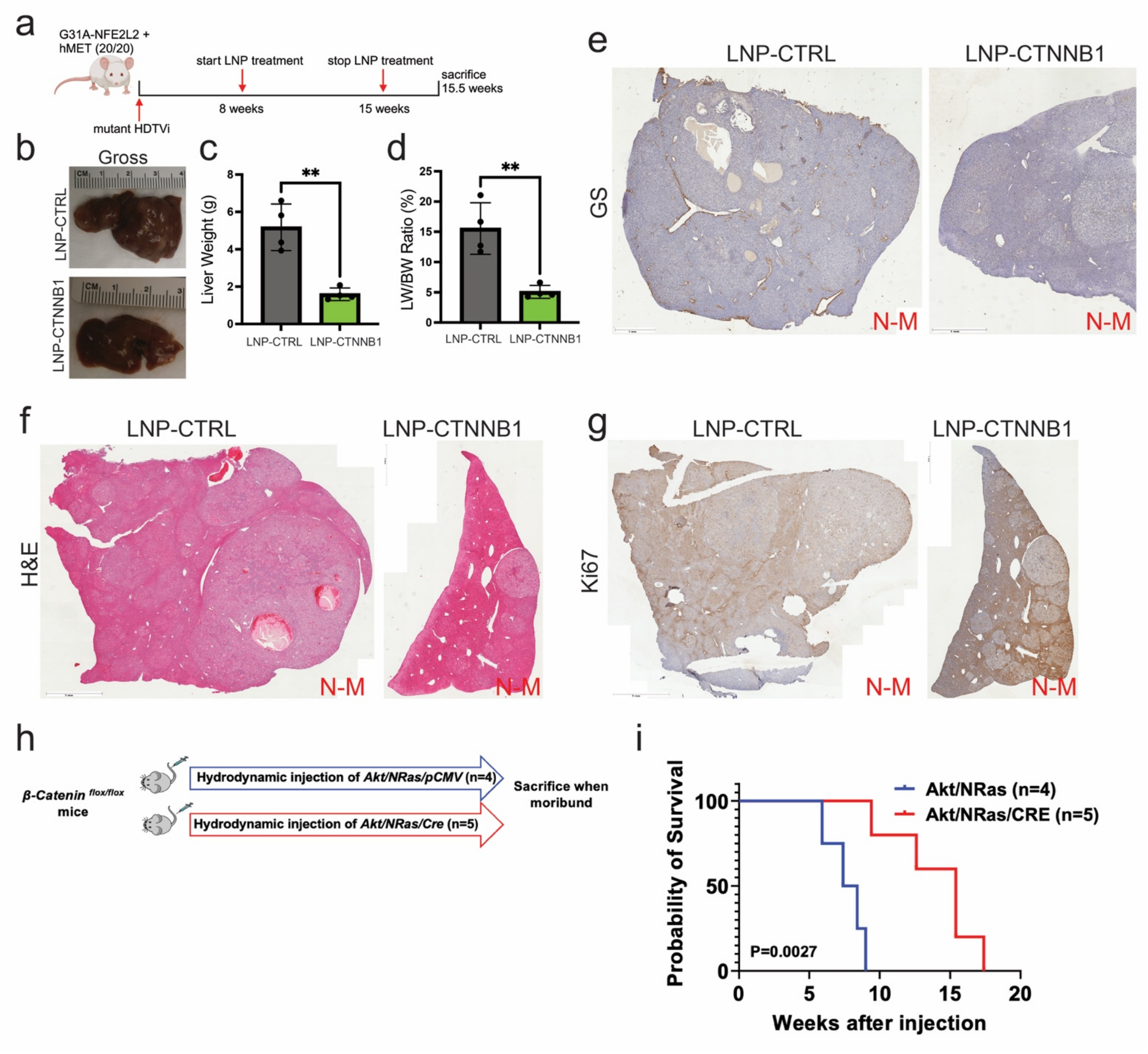

Supplementary Figure 4. RNAi-mediated β-catenin inhibition or β-catenin knockout in *CTNNB1*-wild-type HCC models leads to partial responses (related to Figure 1).

(a) LNP treatment scheme in Nrf2-hMet (N-M) model. Mice received once weekly I.V. injections at 1mg/kg dosage starting at 8-weeks post-hydrodynamic tail vein injection (HDTVi). Created in BioRender. Lehrich, B. (2025) https://BioRender.com/gad9tr4.

(b) Representative gross liver images of LNP-CTRL and LNP-CTNNB1 (1mg/kg) treated N-M animals at 15.5-week timepoint.

(c) Liver weights comparing LNP-CTRL (n=4) and LNP-CTNNB1 (n=4; 1mg/kg) treated N-M animals at 15.5-week timepoint. Data presented as mean with error bars representing standard deviation (SD). **p<0.01 calculated by unpaired two-tailed Student's t-test. Exact p-values in source data file.

(d) Liver weight/body weight (LW/BW) ratio comparing LNP-CTRL (n=4) and LNP-CTNNB1 (n=4; 1mg/kg) treated N-M animals at 15.5-week timepoint. Data presented as mean with error bars representing SD. **p<0.01 calculated by unpaired two-tailed Student's t-test. Exact p-values in source data file.

(e) Representative tiled images of immunohistochemistry (IHC) for GS comparing LNP-CTRL and LNP-CTNNB1 (1mg/kg) treated N-M animals at 15.5-week timepoint. Scale bar indicates magnification.

(f) Representative tiled images of hematoxylin and eosin (H&E) staining comparing LNP-CTRL and LNP-CTNNB1 (1mg/kg) treated Nrf2-hMet animals at 15.5-week timepoint. Scale bar indicates magnification.

(g) Representative tiled images of Ki67 IHC comparing LNP-CTRL and LNP-CTNNB1 (1mg/kg) treated Nrf2-hMet animals at 15.5-week timepoint. Scale bar indicates magnification.

(h) Schematic illustrating β-catenin deletion in *Ctnnb1*$^{flox/flox}$ mice via pCMV-Cre at time of HDTVi. Mice were sacrificed when moribund. Graphic created in BioRender.

(i) Kaplan-Meier curve of overall survival demonstrating significantly longer survival time in Akt/NRas/pCMV-Cre (n=5) mice compared to Akt/NRas/pCMV (n=4) mice. P-value calculated via Kaplan-Meier log-rank test. Exact p-values in source data file.

Supplementary Figure 5

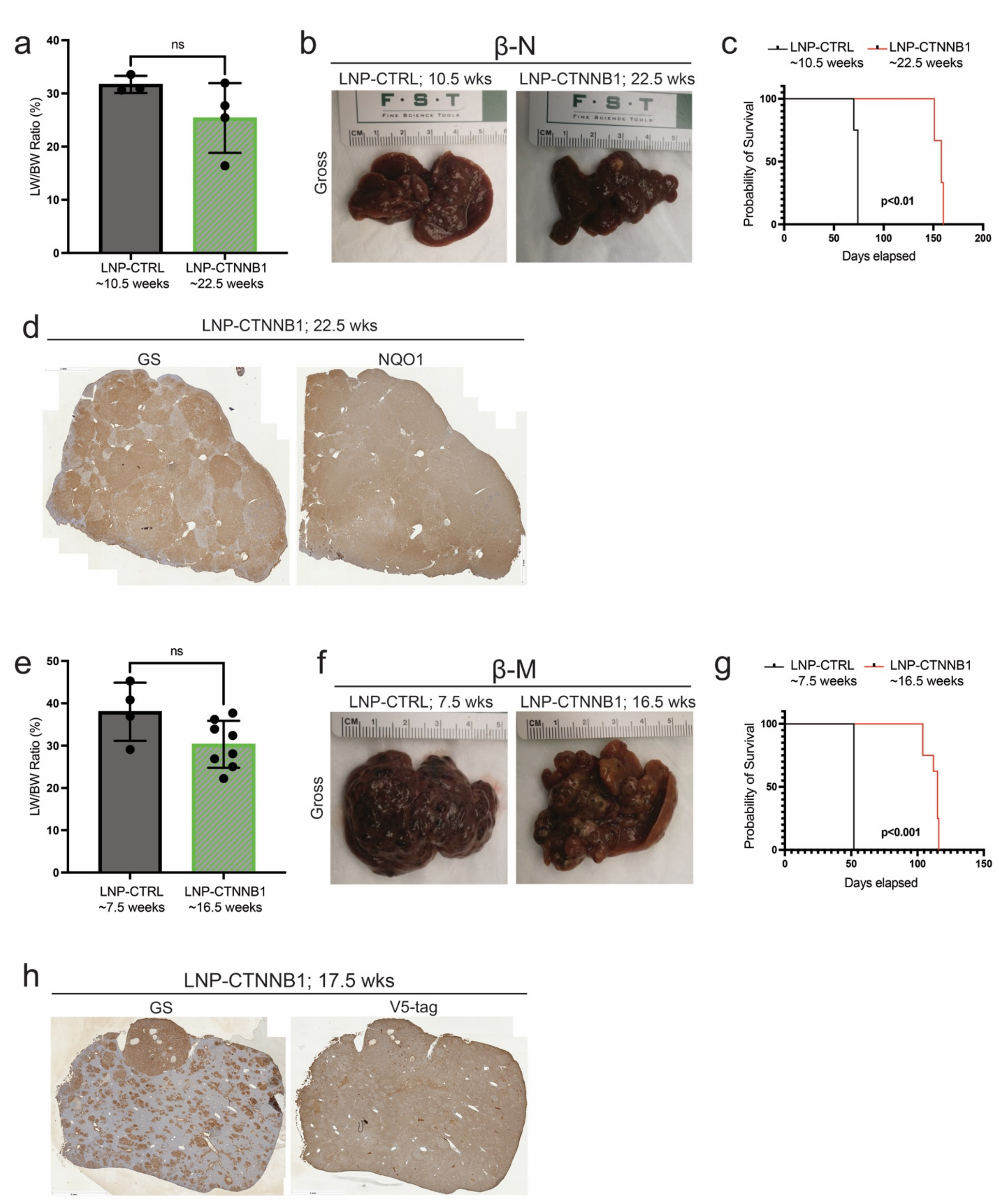

Supplementary Figure 5. RNAi-mediated β-catenin inhibition in early-stage disease leads to durable response and improved overall survival in multiple immunocompetent *CTNNB1*-mutated HCC mouse models (related to Figure 1).

(a) Liver weight/body weight (LW/BW) ratio comparing LNP-CTRL (n=3) at 10.5-weeks and LNP-CTNNB1 (n=4; 1mg/kg) at 22.5-weeks post-hydrodynamic tail vein injection (HDTVi) in β-catenin-Nrf2 (β-N) model. For LNP-CTNNB1 (1mg/kg) treated β-N mice, LNP treatment followed same treatment scheme as in Figure 1c and then mice were monitored weekly for morbidiy. Data presented as mean with error bars representing standard deviation (SD). P-value calculated by unpaired two-tailed Student's t-test. Exact p-values in source data file.

(b) Representative gross liver images of LNP-CTRL and LNP-CTNNB1 (1mg/kg) treated β-N animals at indicated time points.

(c) Kaplan-Meier survival curve of overall survival comparing β-N treated animals receiving either LNP-CTRL (n=4) or LNP-CTNNB1 (n=6; 1mg/kg). $^{**}p<0.01$ calculated by Kaplan-Meier log-rank test. Exact p-values in source data file.

(d) (Left) Representative tiled images of glutamine synthetase (GS) staining on LNP-CTNNB1 (1mg/kg) treated β-N animals at 22.5-week timepoint. (Right) Representative tiled images of NQO1 (downstream of Nrf2) staining on LNP-CTNNB1 (1mg/kg) treated β-N animals at 22.5-week timepoint. Scale bar indicates magnification.

(e) LW/BW ratio comparing LNP-CTRL (n=4) at 7.5-weeks and LNP-CTNNB1 (n=8; 1mg/kg) at 16.5-weeks post-HDTVi in β-catenin-hMet (β-M) model. For LNP-CTNNB1 (1mg/kg) treated β-M mice, LNP treatment followed same treatment scheme as in Figure 1h and then mice were monitored weekly for morbidiy. Data presented as mean with error bars representing SD. P-value calculated by unpaired two-tailed Student's t-test. Exact p-values in source data file.

(f) Representative gross liver images of LNP-CTRL and LNP-CTNNB1 (1mg/kg) treated β-M animals at indicated time points.

(g) Kaplan-Meier survival curve of overall survival comparing β-M treated animals receiving either LNP-CTRL (n=4) or LNP-CTNNB1 (n=8; 1mg/kg). $^{***}p<0.001$ calculated by Kaplan-Meier log-rank test. Exact p-values in source data file.

(h) (Left) Representative tiled images of GS staining on LNP-CTNNB1 (1mg/kg) treated β-M animals at 16.5-week timepoint. (Right) Representative tiled images of V5-tag (present on hMet plasmid) staining on LNP-CTNNB1 (1mg/kg) treated β-M animals at 16.5-week timepoint. Scale bar indicates magnification.

Supplementary Figure 6

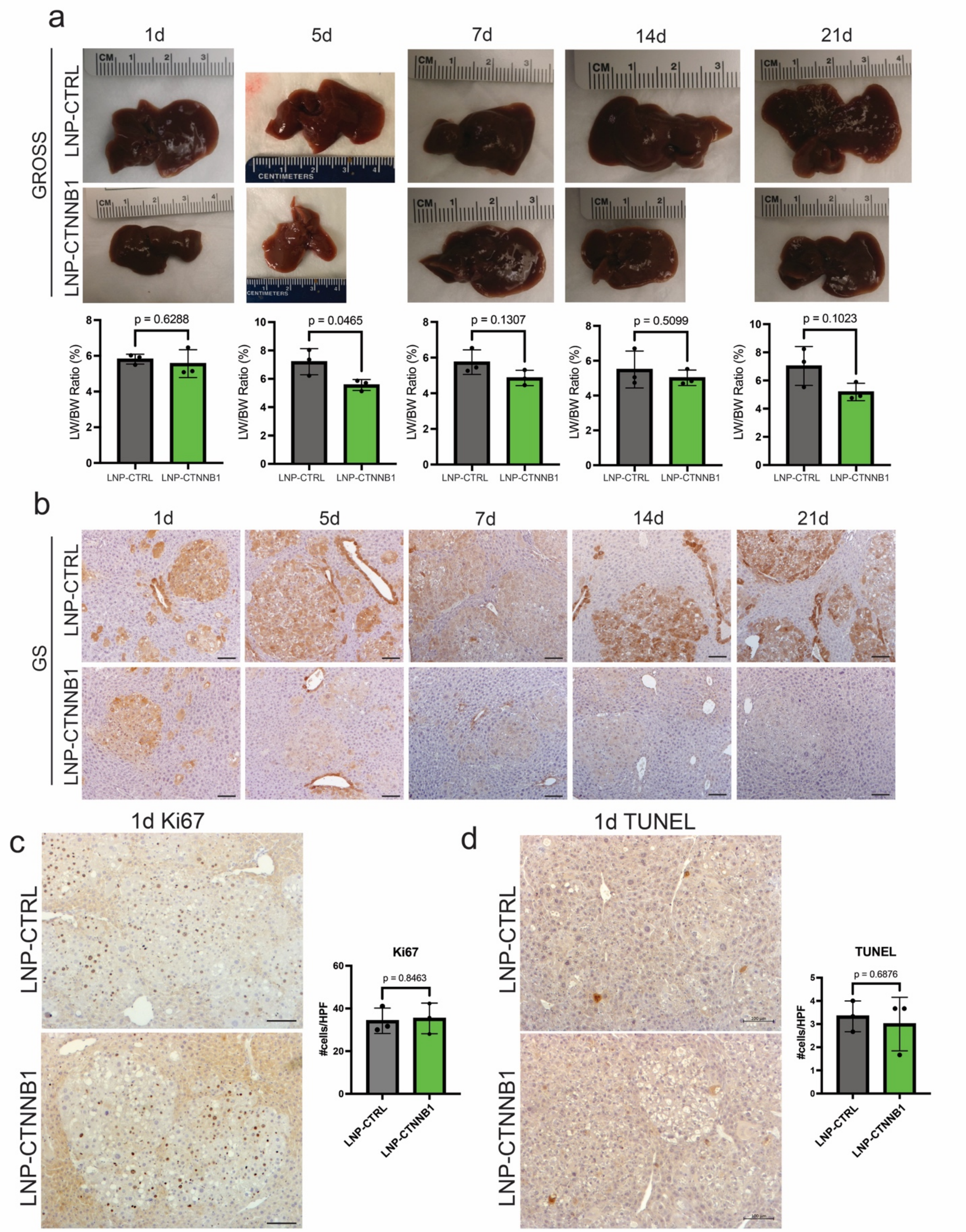

Supplementary Figure 6. Time course of response to RNAi-mediated β-catenin inhibition in β-catenin-Nrf2 (β-N) model in early-stage disease setting (related to Figure 2).

(a) (Top) Representative gross liver images of LNP-CTRL and LNP-CTNNB1 (1mg/kg) treated β-N model animals at indicated time points (1-day, 5-day, 7-day, 14-day, 21-day post-LNP treatment). (Bottom) Liver weight/body weight (LW/BW) ratio comparing LNP-CTRL (n=3 per timepoint) and LNP-CTNNB1 (n=3 per timepoint; 1mg/kg) treated β-N model animals at each of the indicated time points (1-day, 5-day, 7-day, 14-day, 21-day post-LNP treatment). Data presented as mean with error bars representing standard deviation (SD). P-value calculated by unpaired two-tailed Student's t-test. Exact p-values in source data file.

(b) Representative IHC images from LNP-CTRL and LNP-CTNNB1 (1mg/kg) treated β-N model animals at indicated time points (1-day, 5-day, 7-day, 14-day, 21-day post-LNP treatment) for glutamine synthetase (GS). 10X objective magnification for the images. Scale bar indicates 100μm.

(c) (Left) Representative IHC images from LNP-CTRL and LNP-CTNNB1 (1mg/kg) treated β-N model animals at 1-day post-LNP treatment) for Ki67. 10X objective magnification for the images. Scale bar indicates 100μm. (Right) Quantification of number of positive cells across multiple high-power fields (HPF) for Ki67 staining between LNP-CTRL and LNP-CTNNB1 (1mg/kg) treated β-N animals 1-days post 1st LNP treatment. Quantification performed from n=3 biological replicates for each LNP treatment condition. Data presented as mean with error bars representing standard deviation (SD). P-value calculated by unpaired two-tailed Student's t-test. Exact p-values in source data file.

(d) (Left) Representative IHC images from LNP-CTRL and LNP-CTNNB1 (1mg/kg) treated β-N model animals at 1-day post-LNP treatment) for TUNEL. 10X objective magnification for the images. Scale bar indicates 100μm. (Right) Quantification of number of positive cells across multiple high-power fields (HPF) for TUNEL staining between LNP-CTRL and LNP-CTNNB1 (1mg/kg) treated β-N animals 1-days post 1st LNP treatment. Quantification performed from n=3 biological replicates for each LNP treatment condition. Data presented as mean with error bars representing SD. P-value calculated by unpaired two-tailed Student's t-test. Exact p-values in source data file.

Supplementary Figure 7

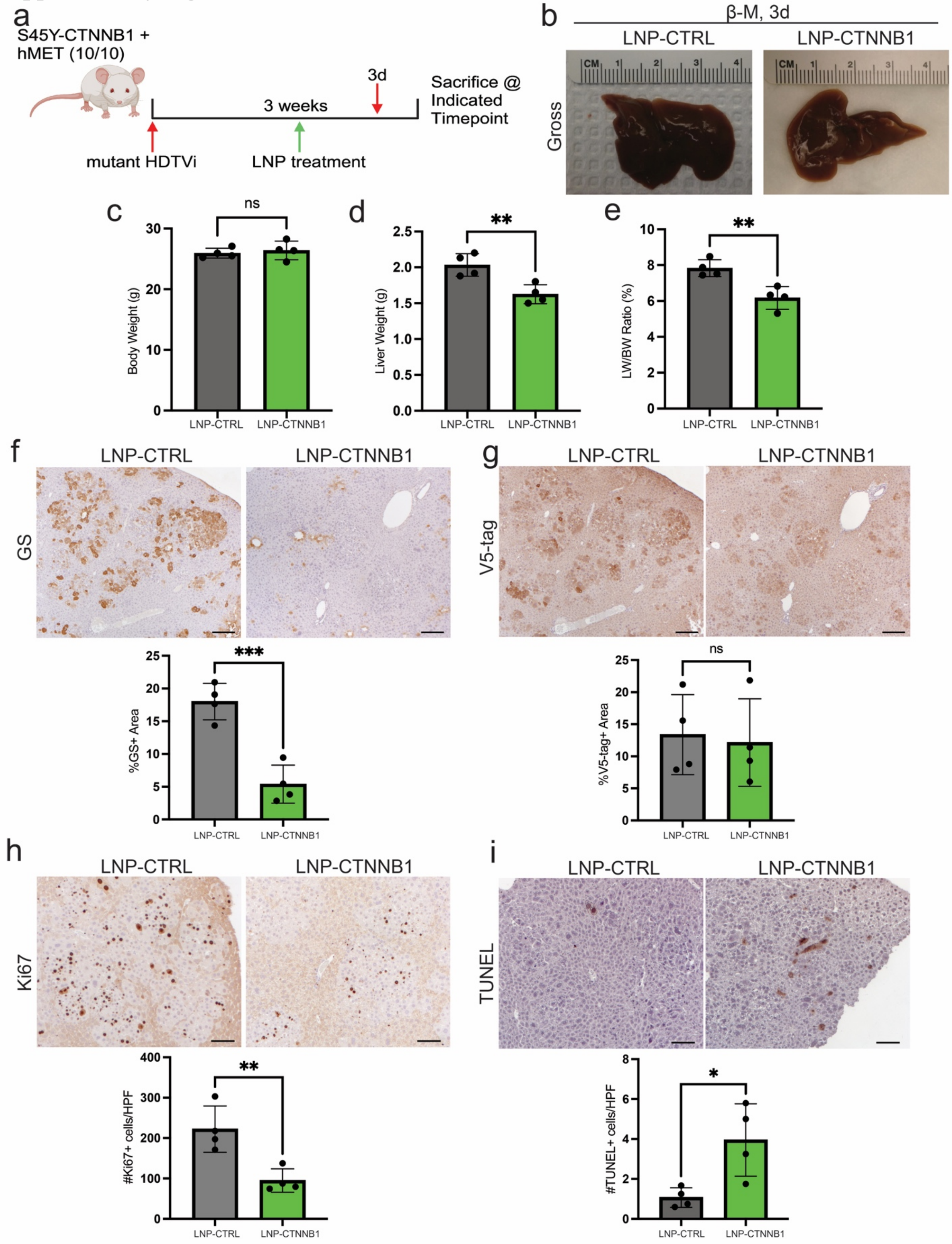

Supplementary Figure 7. Earliest biological response to RNAi-mediated β-catenin inhibition observed 3-days after LNP treatment in β-catenin-hMet (β-M) model (related to Figure 2).

(a) LNP treatment scheme in β-M model. Mice received one LNP treatment at 1mg/kg dosage at 3-weeks post-hydrodynamic tail vein injection (HDTVi) and sacrificed 3-days post-LNP treatment. Created in BioRender. Lehrich, B. (2025) https://BioRender.com/1v2f0xj.

(b) Representative gross liver images of LNP-CTRL and LNP-CTNNB1 treated β-M animals at 3-days post 1st LNP treatment.

(c) Body weights comparing LNP-CTRL (n=4) and LNP-CTNNB1 (n=4; 1mg/kg) treated β-M animals at at 3-days post 1st LNP treatment Data presented as mean with error bars representing standard deviation (SD). P-value calculated by unpaired two-tailed Student's t-test. Exact p-values in source data file.

(d) Liver weights comparing LNP-CTRL (n=4) and LNP-CTNNB1 (n=4; 1mg/kg) treated β-M animals at at 3-days post 1st LNP treatment. Data presented as mean with error bars representing SD. **$p<0.01$ calculated by unpaired two-tailed Student's t-test. Exact p-values in source data file.

(e) Liver weight/body weight (LW/BW) ratio comparing LNP-CTRL (n=4) and LNP-CTNNB1 (n=4; 1mg/kg) treated β-M animals at at 3-days post 1st LNP treatment. Data presented as mean with error bars representing SD. **$p<0.01$ calculated by unpaired two-tailed Student's t-test. Exact p-values in source data file.

(f) (Top) Representative immunohistochemistry (IHC) images from LNP-CTRL and LNP-CTNNB1 (1mg/kg) treated β-M model animals at 3-days post 1st LNP treatment for glutamine synthetase (GS). 5X objective magnification for the images. Scale bar indicates 200μm. (Bottom) Quantification of GS-positive area between LNP-CTRL (n=4) and LNP-CTNNB1 (n=4; 1mg/kg). Data presented as mean with error bars representing SD. ***$p<0.001$ calculated by unpaired two-tailed Student's t-test. Exact p-values in source data file.

(g) (Top) Representative IHC images from LNP-CTRL and LNP-CTNNB1 (1mg/kg) treated β-M model animals at 3-days post 1st LNP treatment for V5-tag (present on hMet plasmid). 5X objective magnification for the images. Scale bar indicates 200μm. (Bottom) Quantification of V5-tag-positive area between LNP-CTRL (n=4) and LNP-CTNNB1 (n=4; 1mg/kg). Data presented as mean with error bars representing SD. P-value calculated by unpaired two-tailed Student's t-test. Exact p-values in source data file.

(h) (Top) Representative IHC images from LNP-CTRL and LNP-CTNNB1 (1mg/kg) treated β-M model animals at 3-days post 1st LNP treatment for Ki67. 10X objective magnification for the images. Scale bar indicates 100μm. (Bottom) Quantification performed from n=4 biological replicates from each LNP treatment condition. Data presented as mean with error bars representing SD. **$p<0.01$ calculated by unpaired two-tailed Student's t-test. Exact p-values in source data file.

(i) (Top) Representative IHC images from LNP-CTRL and LNP-CTNNB1 (1mg/kg) treated β-M model animals at 3-days post 1st LNP treatment for TUNEL. 10X objective magnification for the images. Scale bar indicates 100μm. (Bottom) Quantification performed from n=4 biological replicates from each LNP treatment condition. Data presented as mean with error bars representing SD. *$p<0.05$ calculated by unpaired two-tailed Student's t-test. Exact p-values in source data file.

Supplementary Figure 8

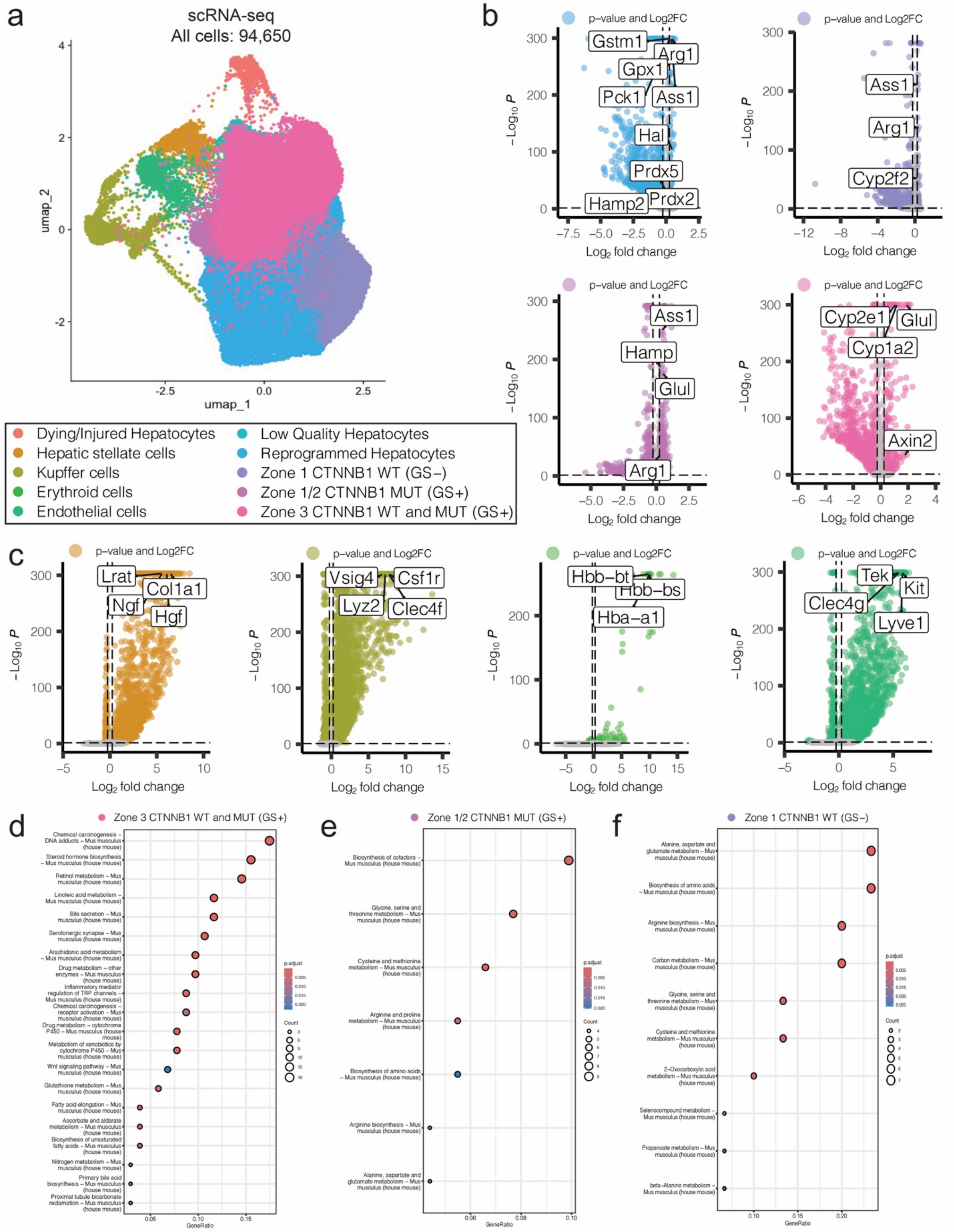

Supplementary Figure 8. Single-cell RNA-sequencing reveals β-catenin-mutated tumor cell metabolic heterogeneity across the liver lobule (related to Figure 3).

(a) Uniform manifold approximation and projection (UMAP) visualization of single-cell RNA-sequencing (scRNA-seq) data following liver perfusion and enrichment of hepatocyte cell populations from LNP-CTRL and LNP-CTNNB1 (1mg/kg) treated β-catenin-Nrf2 (β-N) animals 3-days post LNP treatment. UMAP showing integrated dataset of 94,650 total cells between both treatment conditions. Labeled cell populations indicated by color in box.

(b) Volcano plot highlighting selected differentially expressed genes (DEGs) from the scRNA-seq dataset within the tumor cell and normal hepatocyte populations compared to all other clusters. Marker genes for each population are indicated as follows: reprogrammed hepatocytes (expressing both zone 1 & 2 markers Ar*g1, Ass1, Pck1, Hal, Hamp2*, with tumor markers *Prdx2, Prdx5, Gstm1, Gpx1),* Zone 1 CTNNB1 WT (GS-) hepatocytes (expressing *Cyp2f2, Arg1, Ass1*), Zone 1/2 CTNNB1 MUT (GS+) hepatocytes (expressing *Glul* and *Arg1, Ass1, Hamp*), and Zone 3 CTNNB1 WT & MUT (GS+) hepatocytes (expressing *Axin2, Glul, Cyp2e1, Cyp1a2*).

(c) Volcano plot highlighting selected DEGs from the scRNA-seq dataset within the non-parenchymal cell populations compared to all other clusters. Marker genes for each population are indicated as follows: Hepatic stellate cells (expressing *Lrat, Hgf, Ngf, Col1a1*), Kupffer cells (expressing *Lyz2, Csf1r, Vsig4, Clec4f*), Endothelial cells (expressing *Tek, Kit, Clec4g, Lyve1*), and Erythroid cells (expressing *Hbb-bt, Hba-a1, Hbb-bs*).

(d) Kyoto encyclopedia of genes and genomes (KEGG) pathway enrichment analysis represented as dot plot for top upregulated pathways in Zone 3 CTNNB1 WT & MUT (GS+) hepatocytes.

(e) KEGG pathway enrichment analysis represented as dot plot for top upregulated pathways in Zone 1/2 CTNNB1 MUT (GS+) hepatocytes.

(f) KEGG pathway enrichment analysis represented as dot plot for top upregulated pathways in Zone 1 CTNNB1 WT (GS-) hepatocytes.

Supplementary Figure 9

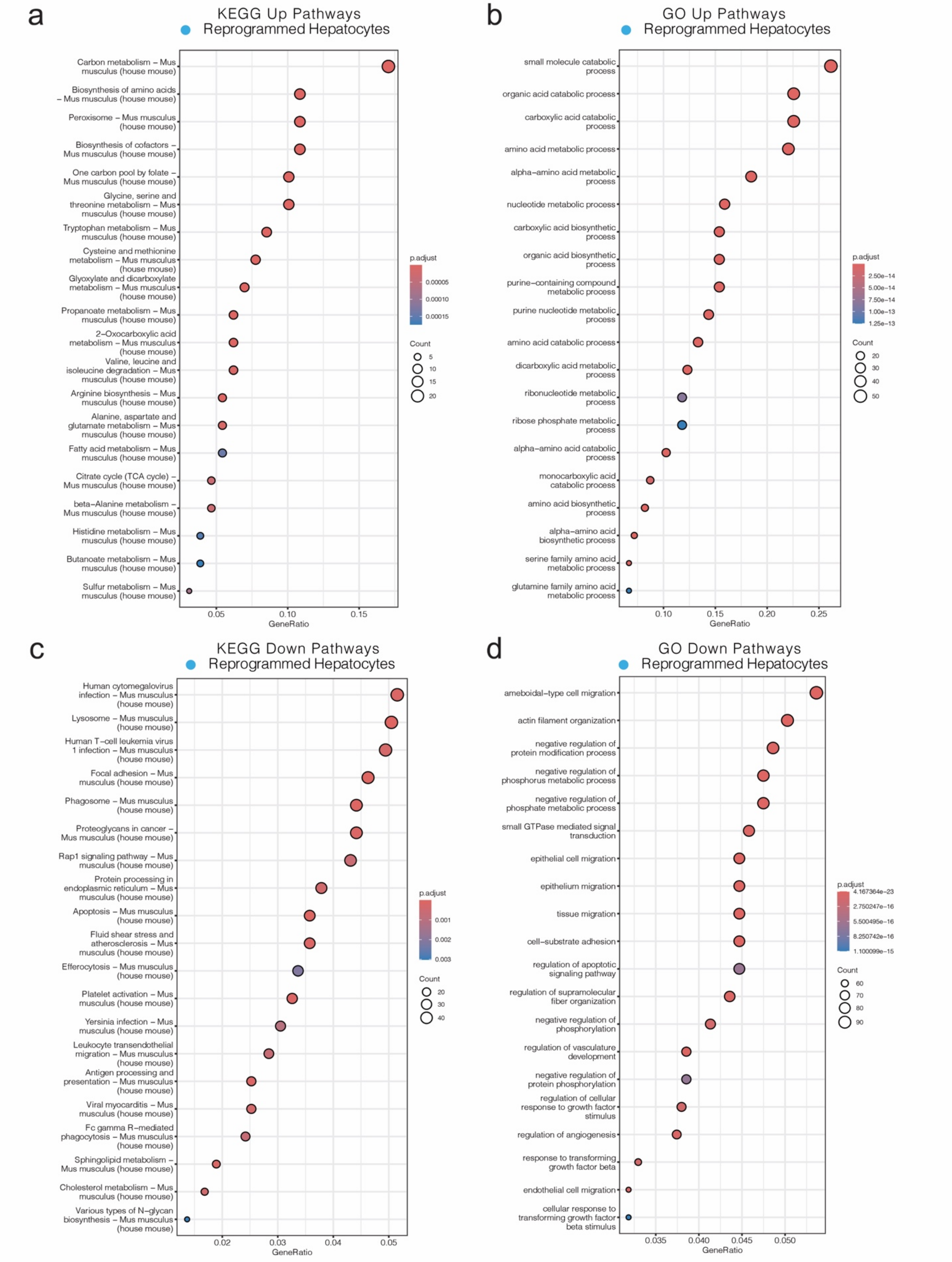

Supplementary Figure 9. Single-cell RNA-sequencing reveals underlying biology of reprogrammed hepatocyte cluster appearing *de novo* following β-catenin inhibition (related to Figure 3).

(a) Kyoto encyclopedia of genes and genomes (KEGG) pathway enrichment analysis represented as dot plot for top upregulated pathways in reprogrammed hepatocytes from scRNA-seq data on hepatocyte enriched cell population. Threshold criteria set as p_adjust <0.05 and average log2FC >0.25.

(b) Gene ontology (GO) pathway enrichment analysis represented as dot plot for top upregulated pathways in reprogrammed hepatocytes from scRNA-seq data on hepatocyte enriched cell population. Threshold criteria set as p_adjust <0.05 and average log2FC >0.25.

(c) KEGG pathway enrichment analysis represented as dot plot for top downregulated pathways in reprogrammed hepatocytes from scRNA-seq data on hepatocyte enriched cell population. Threshold criteria set as p_adjust <0.05 and average log2FC <-0.25.

(d) GO pathway enrichment analysis represented as dot plot for top downregulated pathways in reprogrammed hepatocytes from scRNA-seq data on hepatocyte enriched cell population. Threshold criteria set as p_adjust <0.05 and average log2FC <-0.25.

Supplementary Figure 10

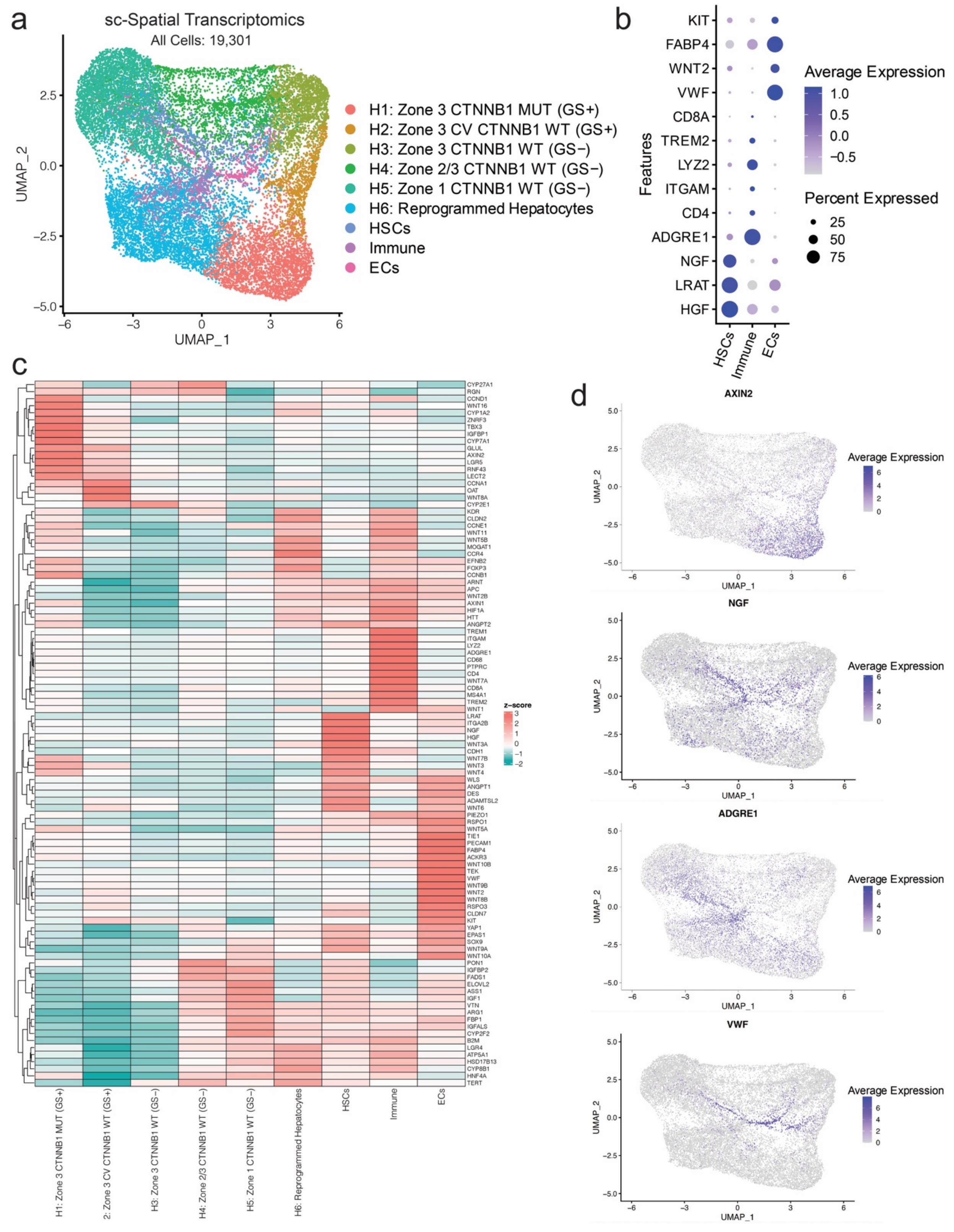

Supplementary Figure 10. Single-cell spatial transcriptomics identifies multiple unique hepatocyte and non-parenchymal cell populations in β-catenin-mutated HCC model (related to Figure 3).

(a) Uniform manifold approximation and projection (UMAP) visualization of single-cell spatial transcriptomic data via Molecular Cartogrpahy^{TM} platform (Resolve Biosciences) performed on frozen liver tissue sections of LNP-CTRL and LNP-CTNNB1 treated β-catenin-Nrf2 (β-N) animals 3-days post LNP treatment. UMAP generated based on expression of 100 genes and showing integrated analysis of both libraries across 19,301 total cells.

(b) Dot plot visualization of various non-parenchymal cell markers, including Hepatic stellate cells (HSCs; *Hgf, Lrat, Ngf*), Immune (*Adgre1, Cd4, Itgam, Lyz2, Trem2, Cd8a*), and Endothelial cells (ECs; *Vwf, Wnt2, Fabp4, Kit*) from (a).

(c) Heatmap of normalized expression of each of the 100 marker genes in the panel showing expression within each of the annotated cell populations. Normalized expression values of the 100 genes across cell types provided in source data file.

(d) UMAP plots visualizing expression of Axin2 (Zone 3 CTNNB1 MUT [GS+]), Ngf (HSCs), Adgre1 (Immune), and Vwf (ECs) to show marker gene expression to the different cell clusters.

Supplementary Figure 11

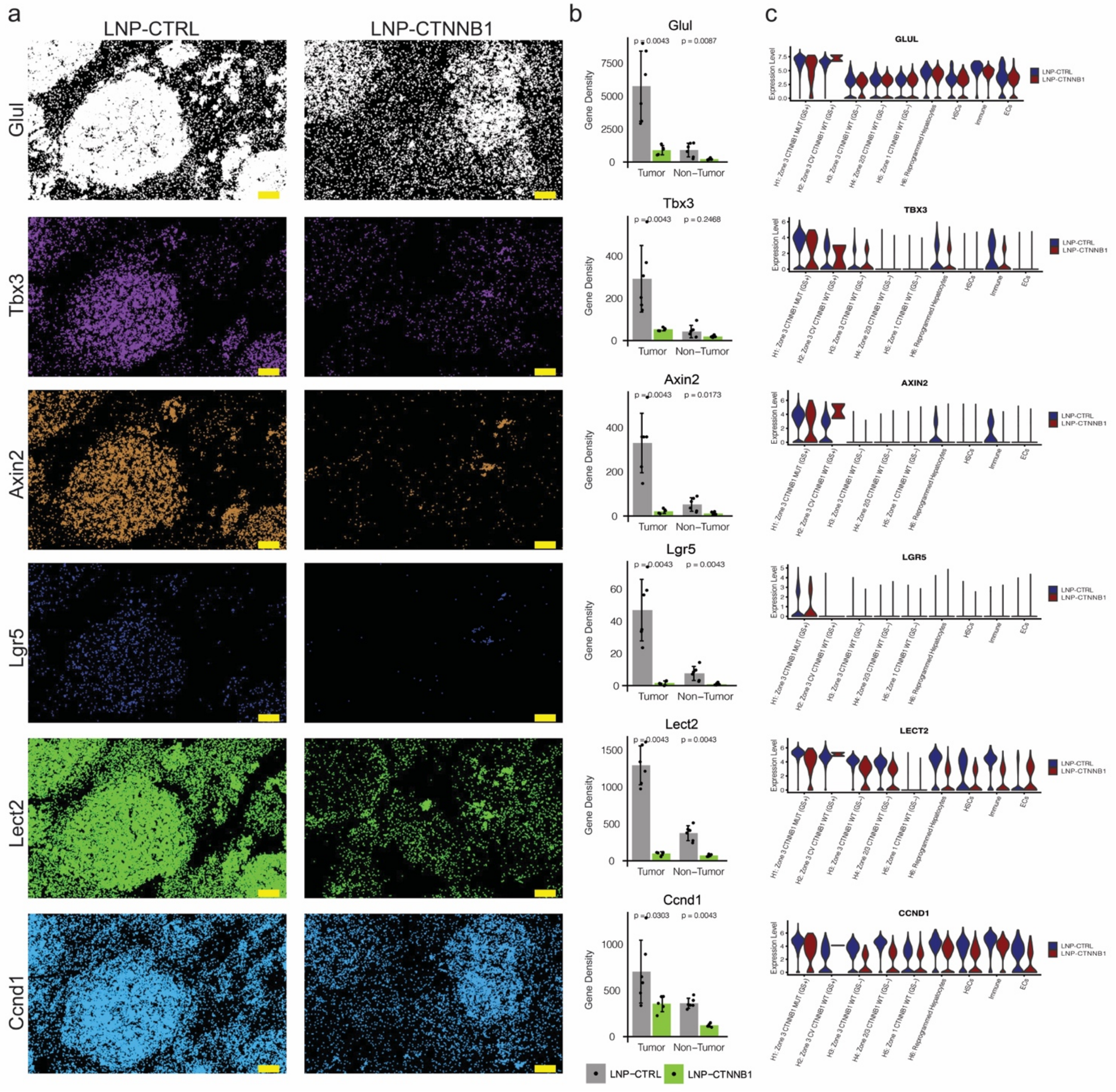

Supplementary Figure 11. Molecular Cartography™ reveals β-catenin-mutated HCC are defined by Wnt/β-catenin target genes (related to Figure 3).

(a) Molecular Cartography™ (Resolve Biosciences) showing expression of Wnt/β-catenin target genes on the tissue section: *Glul* (white), *Tbx3* (purple), *Axin2* (orange), *Lgr5* (blue), *Lect2* (green), and *Ccnd1* (cyan) comparing LNP-CTRL and LNP-CTNNB1 3-days post-treatment in β-catenin-Nrf2 (β-N) model. Images acquired from Resolve Biosciences online analysis platform. Scale bar indicates 100μm.

(b) Gene density quantification of each of the genes from (a) between tumor and non-tumor regions using Glul as a landmark gene to mark tumoral boundaries. Data presented as mean with error bars representing standard deviation (SD). Gene density values provided in source data files. P-values calculated using Wilcoxon signed-rank test.

(c) Violin plots demonstrating expression of each of the genes from (a) within each of the annotated cell types comparing LNP-CTRL and LNP-CTNNB1 3-days post-treatment in β-N model.

Supplementary Figure 12

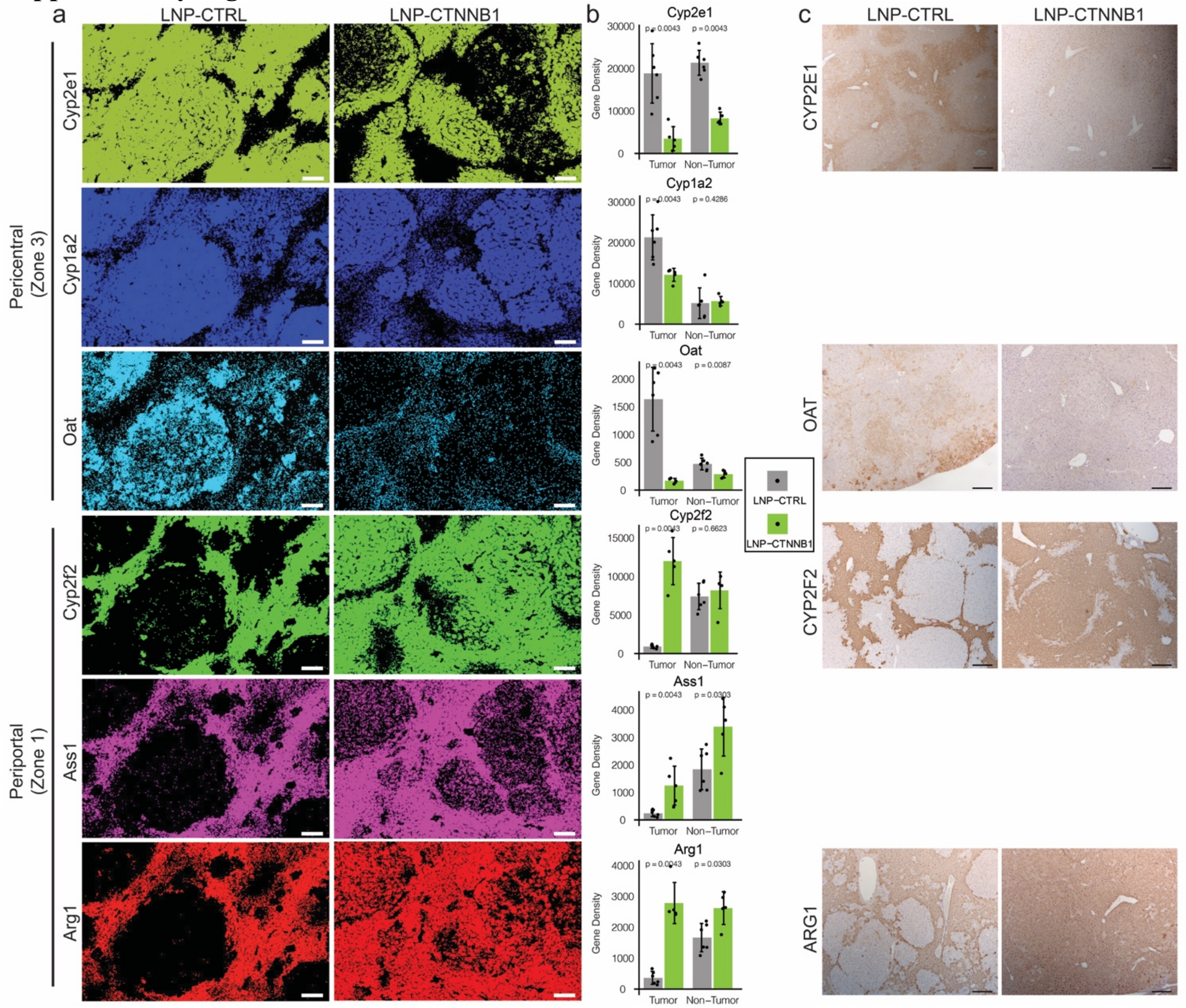

Supplementary Figure 12. Molecular Cartography^{TM} reveals zonated changes within β-catenin-mutated HCC tumor nodules (related to Figure 3).

(a) Molecular Cartography^{TM} (Resolve Biosciences) showing expression of liver zonated genes in LNP-CTRL and LNP-CTNNB1 treated β-catenin-Nrf2 (β-N) animals 3-days post LNP treatment. (Top) Pericentral (zone 3) metabolic genes, including *Cyp2e1* (green), *Cyp1a2* (blue), *Oat* (cyan). (Bottom) Periportal (zone 1) metabolic genes, including *Cyp2f2* (light green), *Ass1* (pink), and *Arg1* (red). Images acquired from Resolve Biosciences online analysis platform. Scale bar indicates 100μm.

(b) Gene density quantification of each of the genes from (a) between tumor and non-tumor regions using Glul as a landmark gene to mark tumor boundaries. Data presented as mean with error bars representing standard deviation (SD). Gene density values provided in source data files. P-values calculated using Wilcoxon signed-rank test.

(c) Representative immunohistochemistry (IHC) images from LNP-CTRL and LNP-CTNNB1 (1mg/kg) treated β-N model animals at 5-days post 1st LNP treatment for CYP2E1, OAT, CYP2F2, and ARG1. IHC repeated at least twice for each stain in multiple animals. 5X objective magnification for the images. Scale bar indicates 200μm.

Supplementary Figure 13

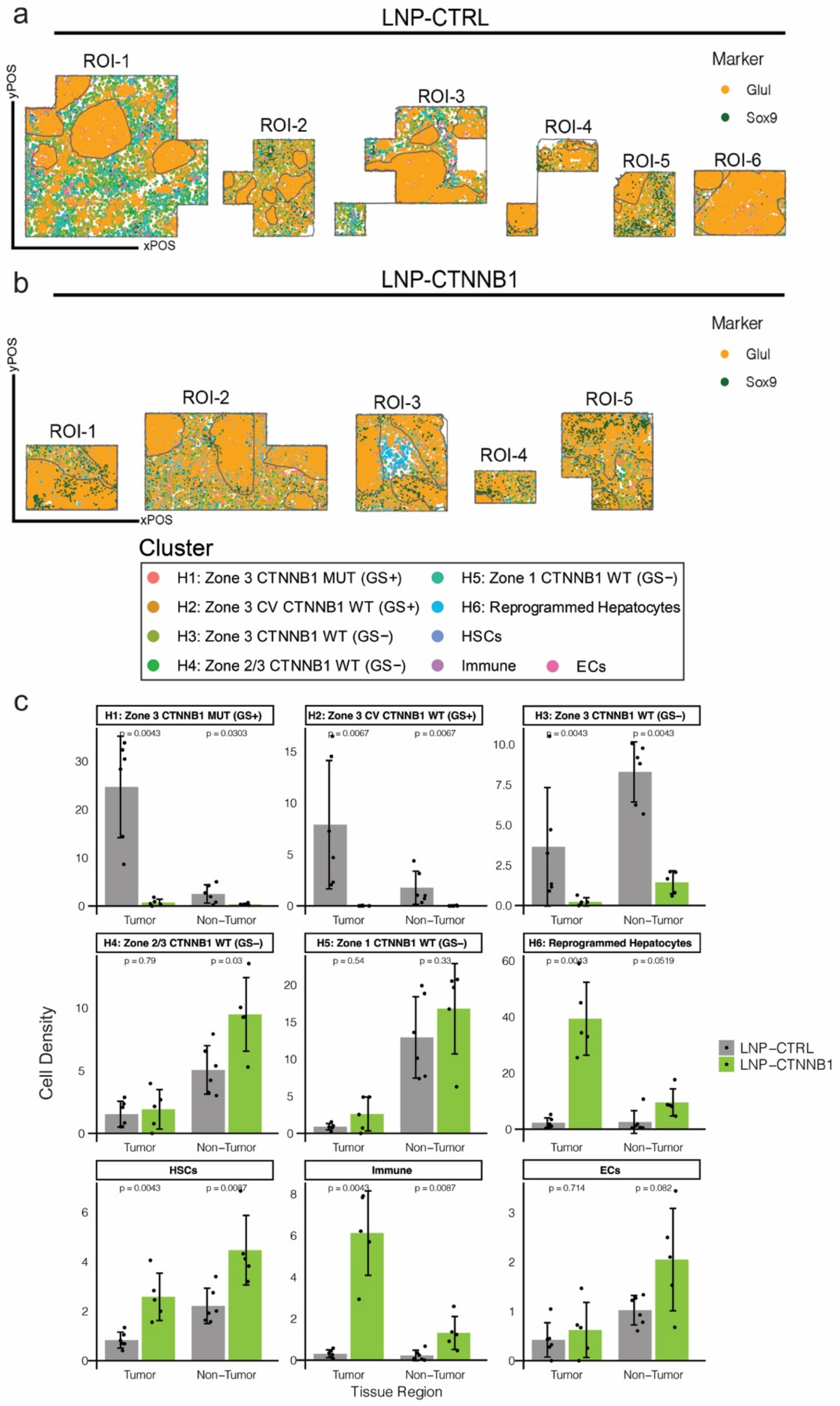

Supplementary Figure 13. Spatial plots for each region of interest by treatment condition from Molecular Cartography™ (Resolve Biosciences) (related to Figure 3).

(a) Spatial plots of the 9 annotated cell populations across the 6 different regions of interest (ROIs) for LNP-CTRL treated β-catenin-Nrf2 (β-N) animals 3-days post LNP treatment. Tissue section areas are colored by gene expression of *Glul* (yellow) and *Sox9* (green) landmark genes, along with by cell cluster.

(b) Spatial plots of the 9 annotated cell populations across the 5 ROIs for LNP-CTNNB1 treated β-N animals 3-days post LNP treatment. Tissue section areas are colored by gene expression of *Glul* (yellow) and *Sox9* (green) landmark genes, along with by cell cluster.

(c) Bar plot detailing quantification of cell cluster density in outlined tumoral and non-tumoral regions based on *Glul* expression outlining tumoral boundaries for LNP-CTRL and LNP-CTNNB1 treated β-catenin-Nrf2 (β-N) animals 3-days post LNP treatment. Data presented as mean with error bars representing standard deviation (SD). Cell density values provided in source data files. P-values calculated using Wilcoxon signed-rank test.

Supplementary Figure 14

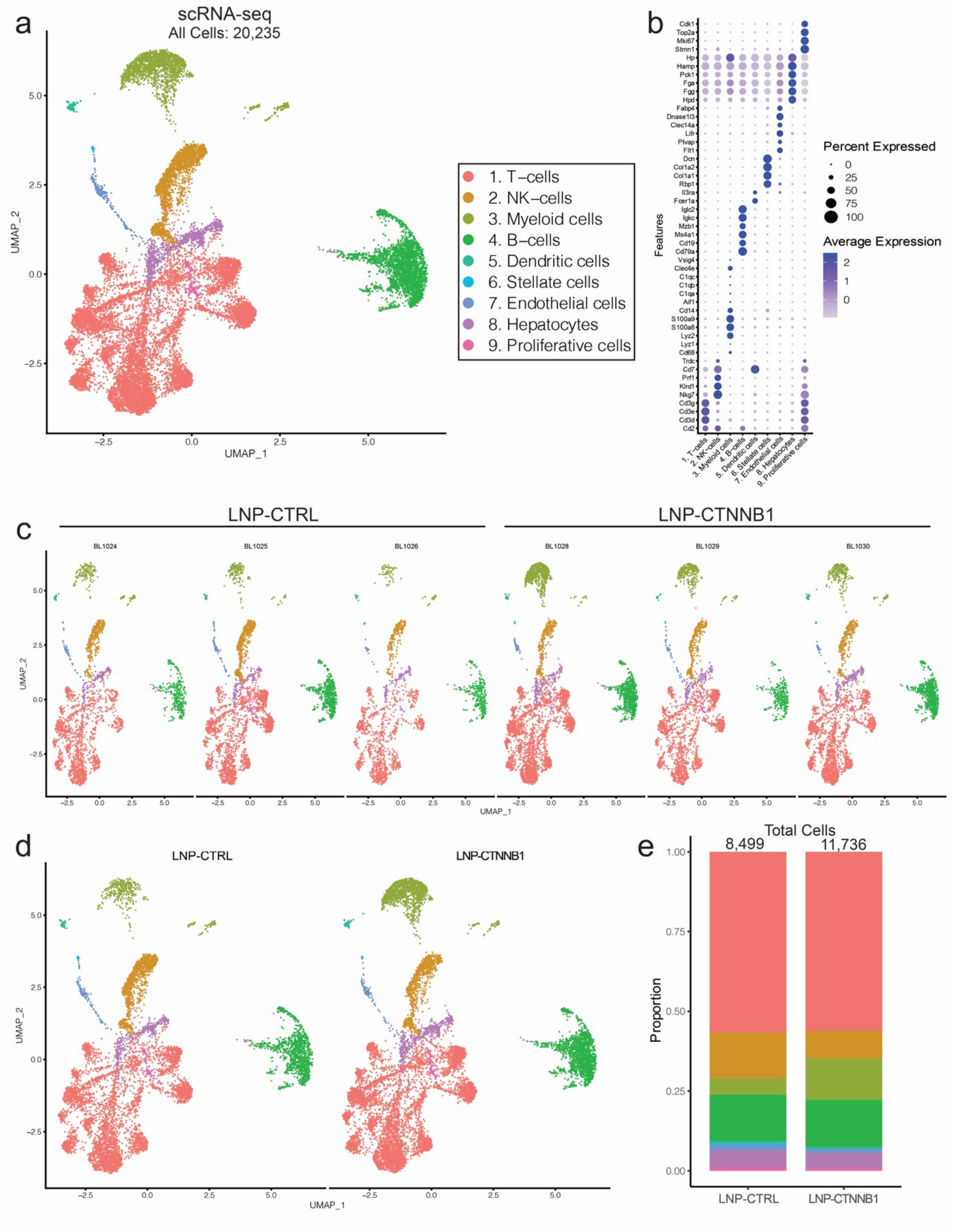

Supplementary Figure 14. Single-cell RNA-sequencing on immune cells reveals majority of T cells and myeloid cells in the β-catenin-mutated HCC tumor immune microenvironment (related to Figure 4).

(a) Uniform manifold approximation and projection (UMAP) visualization of single-cell RNA-sequencing (scRNA-seq) data following liver perfusion and enrichment of immune cell populations from LNP-CTRL and LNP-CTNNB1 (1mg/kg) treated β-catenin-Nrf2 (β-N) animals 3-days post LNP treatment. UMAP represents all cells (20,235).

(b) Dot plot showing expression by cluster for specific marker genes used to label each of the annotated cell types from UMAP in (a).

(c) UMAP from (a) split by LNP-CTRL library across the 3 replicates and LNP-CTNNB1 library across the 3 replicates. Clusters indicated by matching color to UMAP in (a).

(d) UMAP from (a) split by the two treatment conditions (LNP-CTRL, LNP-CTNNB1). Clusters indicated by matching color to UMAP in (a).

(e) Stacked bar plot of cell type proportions between LNP-CTRL and LNP-CTNNB1 treatment conditions based on UMAP from (d). Clusters indicated by matching color to UMAP in (a). Cell type proportion values provided in source data file.

Supplementary Figure 15

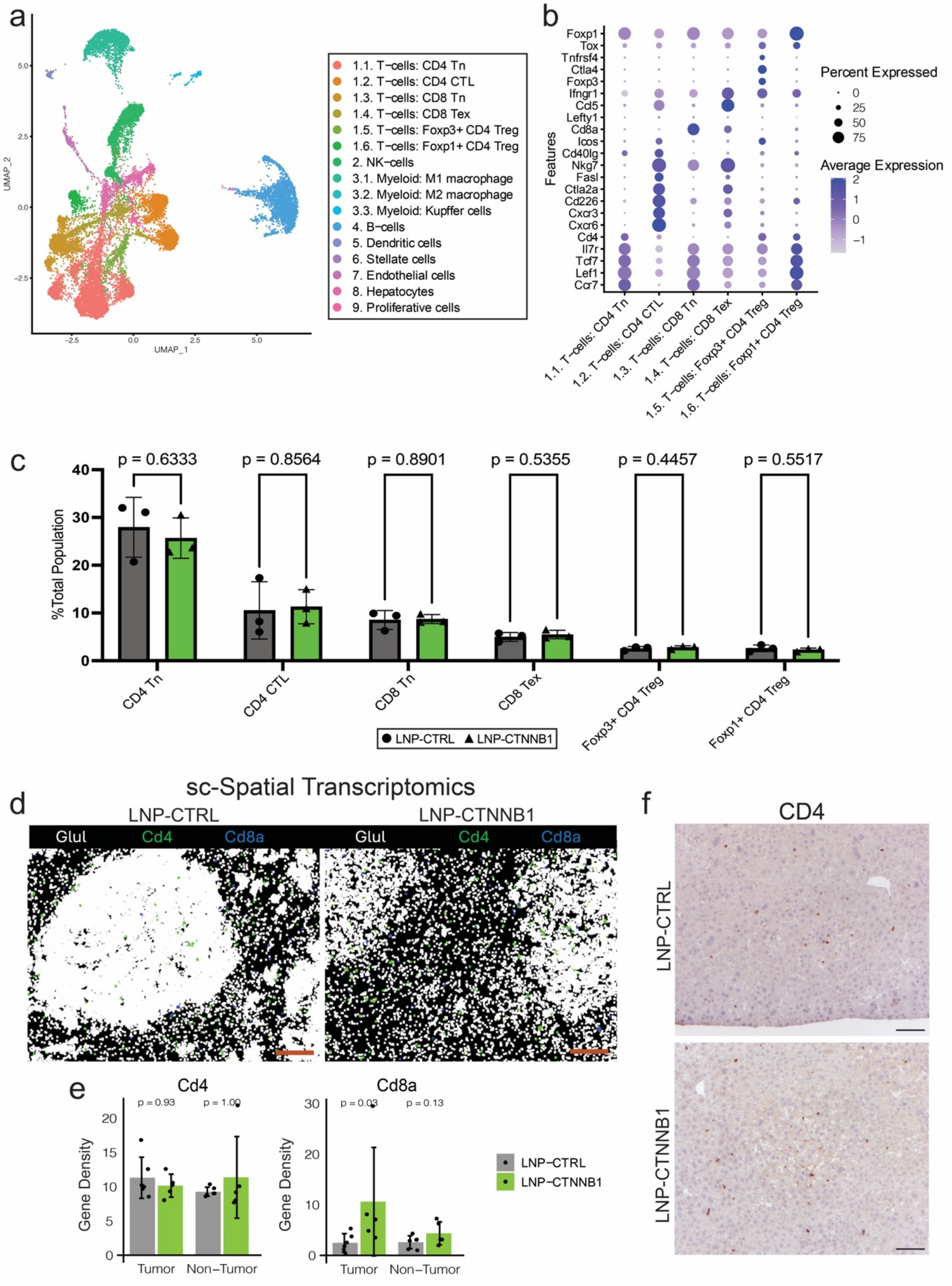

Supplementary Figure 15. Integrated single-cell analyses reveal adaptive immune surveillance is not activated 3-days post LNP-CTNNB1 treatment (related to Figure 4).

(a) Uniform manifold approximation and projection (UMAP) visualization of single-cell RNA-sequencing (scRNA-seq) data from Figure S14a further annotated and subclustered for different T cell functional states and myeloid functional states. Cell types are indicated by color. UMAP represents all cells (n=20,235).

(b) Dot plot showing expression by cluster for various marker genes used to annotate different subclusters from UMAP in (a) of the original T cell cluster from Figure S14a.

(c) Bar plot detailing quantification of the different T cell subclusters in (a) and (b) by percent of total population for LNP-CTRL (n=3) and LNP-CTNNB1 (n=3; 1mg/kg). Data presented as mean with error bars representing standard deviation (SD). P-values calculated by unpaired two-tailed Student's t-test.

(d) Molecular Cartography^{TM} (Resolve Biosciences) showing spatial gene expression of *Glul* (white), *Cd4* (green), and *CD8a* (blue) in β-N animals treated with either LNP-CTRL and LNP-CTNNB1 (1mg/kg). Scale bar indicates 100μm.

(e) Bar plot detailing gene density quantification of *Cd4* and *Cd8a* in outlined tumoral and non-tumoral regions based on *Glul* expression as landmark gene for LNP-CTRL and LNP-CTNNB1 (1mg/kg) treated β-catenin-Nrf2 (β-N) animals 3-days post LNP treatment. Data presented as mean with error bars representing SD. P-values calculated using Wilcoxon signed-rank test. Gene density values provided in source data files.

(f) Representative immunohistochemistry (IHC) images from LNP-CTRL and LNP-CTNNB1 (1mg/kg) treated β-M animals at 3-day post-LNP treatment) for CD4. IHC repeated at least twice in multiple replicate animals. 10X objective magnification for the images. Scale bar indicates 100μm.

Supplementary Figure 16

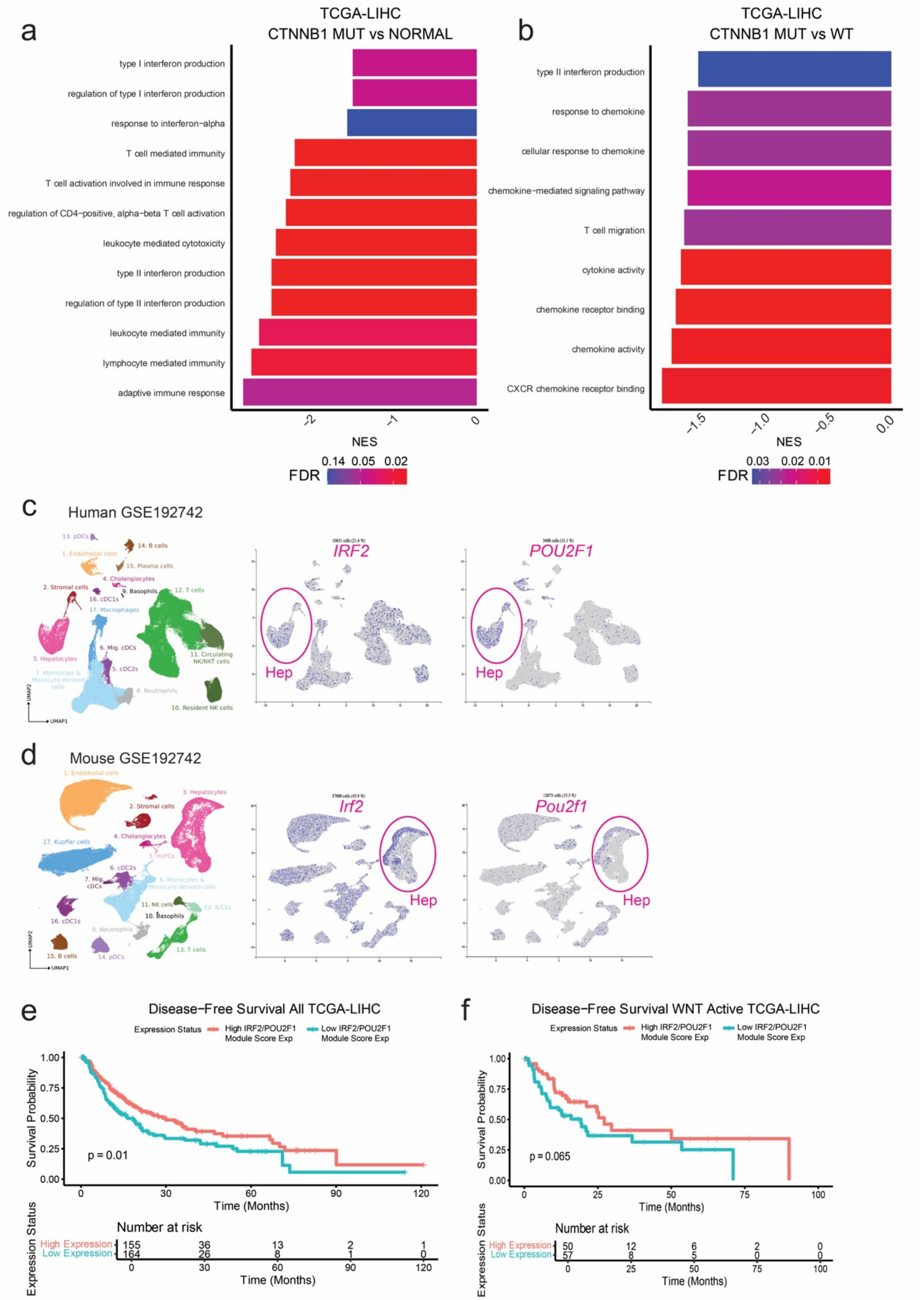

Supplementary Figure 16. Interferon signaling is downregulated in *CTNNB1*-mutated HCC and both IRF2 and POU2F1 are hepatocyte expressed transcription factors in both human and mouse normal liver (related to Figure 5).

(a) Gene ontology (GO) Pathway gene set enrichment analysis (GSEA) comparing CTNNB1-mutated HCC patients (n=98) vs adjacent normal (n=50) from TCGA-LIHC. Pathways ranked by normalized enrichment score (NES) and false discovery rate (FDR). NES values provided in source data file.

(b) GO Pathway GSEA comparing CTNNB1-mutated HCC patients (n=98) vs CTNNB1-wild-type (n=276) from TCGA-LIHC. Pathways ranked by NES and FDR. NES values provided in source data file.

(c) (Left) Uniform manifold approximation and projection (UMAP) visualization of single-cell RNA-sequencing (scRNA-seq) data taken from GSE192742 (https://www.livercellatlas.org/index.php) of all human liver cells annotated by cell type. (Middle) *IRF2* expression by cell type on UMAP demonstrating hepatocytes express *IRF2* in human liver parenchyma. (Right) *POU2F1* expression by cell type on UMAP demonstrating hepatocytes express *POU2F1* in human liver parenchyma. Images downloaded directly from the website.

(d) (Left) UMAP visualization of scRNA-seq data taken from GSE192742 (https://www.livercellatlas.org/index.php) of all mouse liver cells annotated by cell type. (Middle) *Irf2* expression by cell type on UMAP demonstrating hepatocytes express *Irf2* in mouse liver parenchyma. (Right) *Pou2f1* expression by cell type on UMAP demonstrating hepatocytes express *Pou2f1* in mouse liver parenchyma. Images downloaded directly from the website.

(e) Kaplan-Meier disease-free survival (DFS) curve comparing high vs low expression (based on median expression) of IRF2/POU2F1 target gene signature score in all TCGA-LIHC patients. P-value calculated via log-rank test.

(f) Kaplan-Meier DFS curve comparing high vs low expression (based on median expression) of IRF2/POU2F1 target gene signature score in Wnt-active (*CTNNB1-, AXIN1*-, and *APC*-mutated) TCGA-LIHC patients. P-value calculated via log-rank test.

Supplementary Figure 17

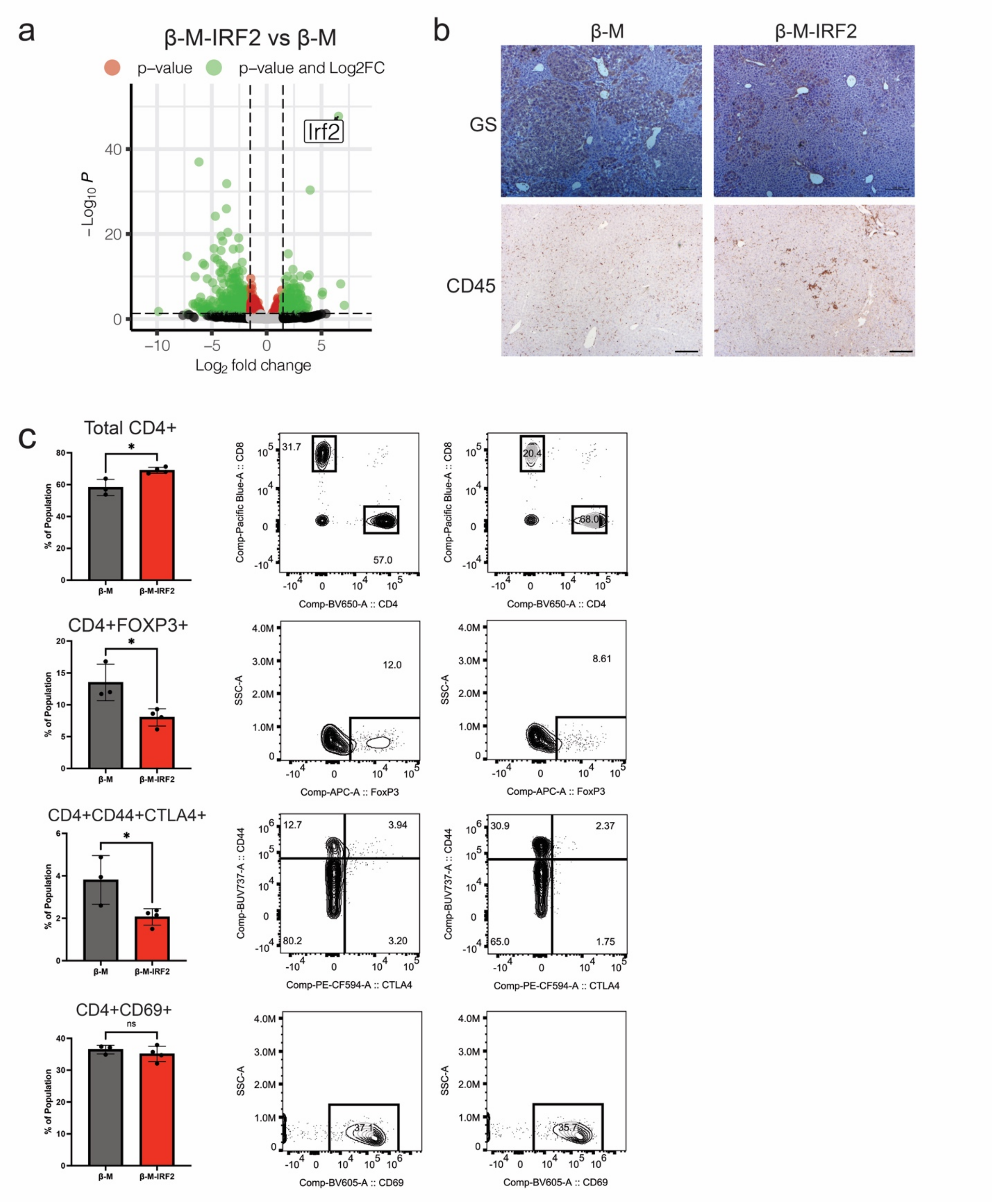

Supplementary Figure 17. IRF2 repression by mutated-β-catenin drives tumor progression and promotes an immune excluded tumor immune microenvironment in β-catenin-mutated HCC (related to Figure 5).

(a) Volcano plot highlighting *Irf2* as a differentially expressed gene comparing β-M-pT3 (n=2) and β-M-IRF2 (n=4) animals at 7.5-week timepoint. DEGs defined by p_adjust<0.05 and log2FC > 1.5.

(b) Representative immunohistochemistry (IHC) images from β-M-pT3 and β-M-IRF2 animals at 7.5-week timepoint stained for glutamine synthetase (GS) or CD45. IHC repeated at least twice for the indicated stains in multiple replicate animals. 10X objective magnification for the images. Scale bar indicates 100μm.

(c) (Left) Bar graphs of cell populations from fluorescence-activated cell sorting (FACS) data comparing β-M-pT3 (n=3) and β-M-IRF2 (n=4) animals at 7.5-week timepoint for total CD4+, CD4+FOXP3+, CD4+CD44+CTLA4+, and CD4+CD69+ cells. Data presented as mean with error bars representing standard deviation (SD). P-values calculated by unpaired two-tailed Student's t-test. Exact p-values provided in source data file. (Right) FACS plots from representative animals β-M-pT3 and β-M-IRF2 animals at 7.5-week timepoint for each of the indicated cell populations from the bar graphs.

Supplementary Figure 18

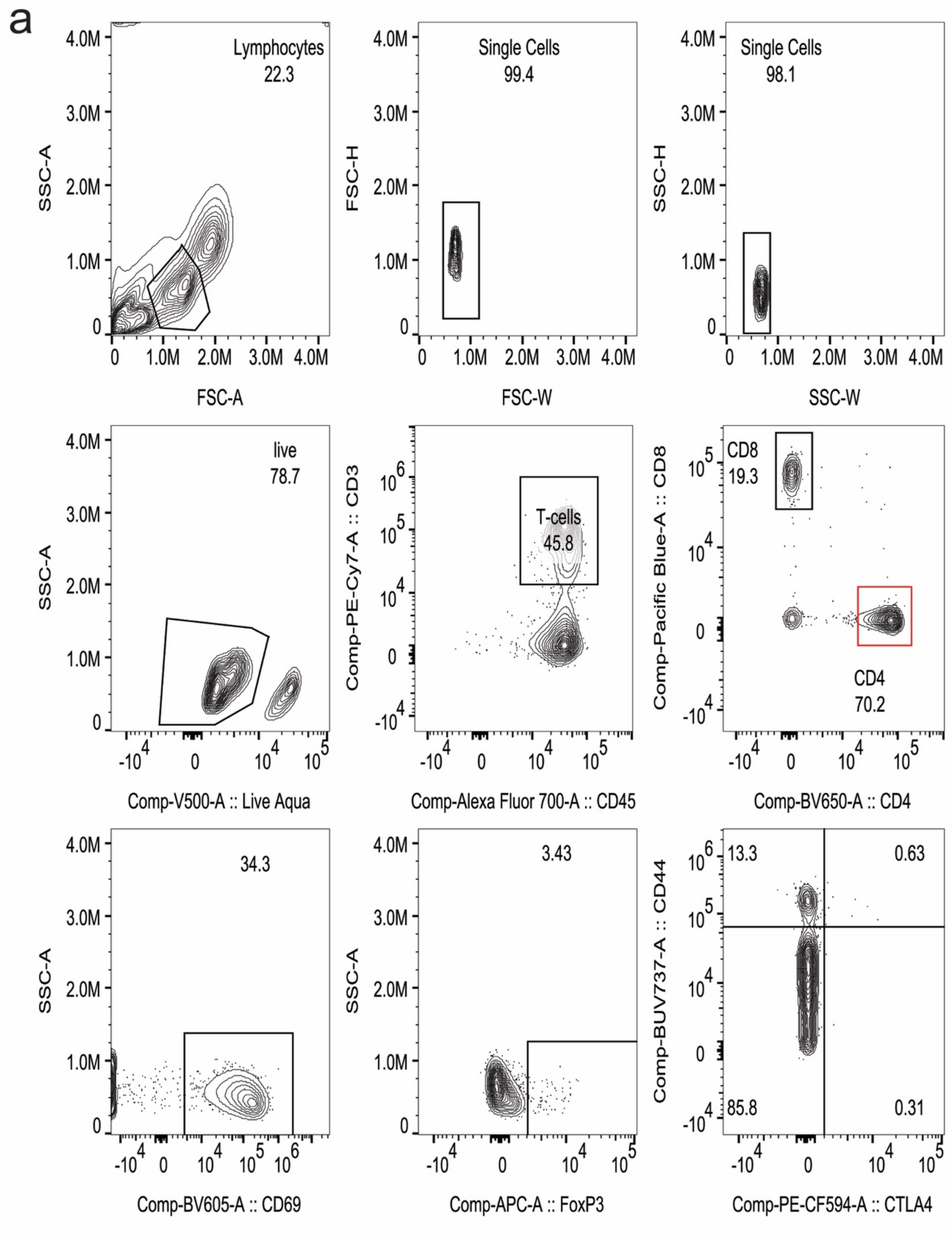

Supplementary Figure 18. Fluorescence-activated cell sorting gating strategy for identifying adaptive immune cell populations from Supplementary Figure 17 (related to Figure 5).

(a) Gating strategy for Supplementary Figure 17C. Lymphocytes were initially gated based on live/dead markers, then T cells identified by CD3 expression with CD4 cells then identified. From there, CD69 cells were gated and markers such as FOXP3 and CTLA4 were subsequently gated on.

Supplementary Figure 19

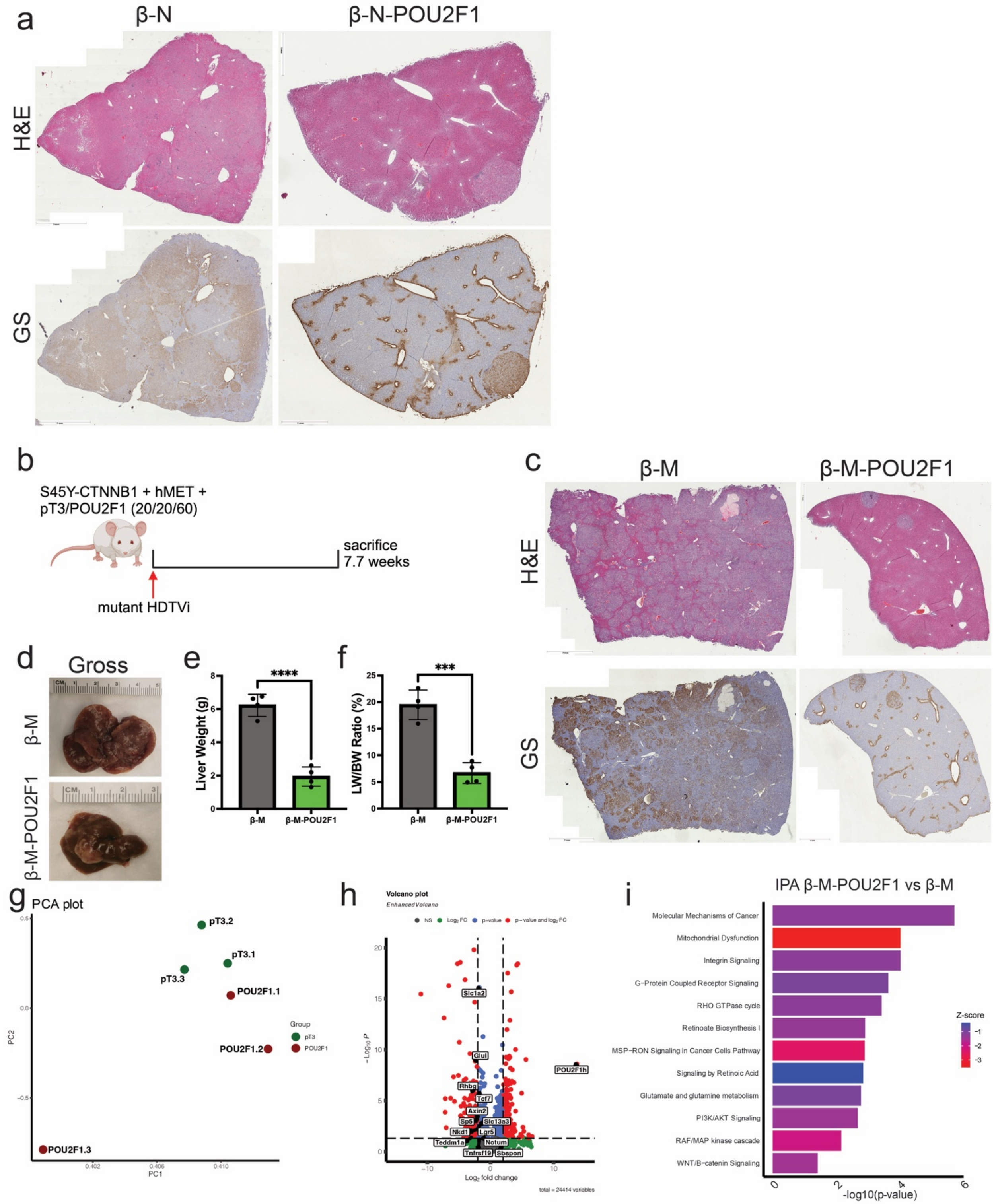

Supplementary Figure 19. POU2F1 repression by mutated-β-catenin drives tumor progression and promotes an immune excluded tumor immune microenvironment in β-catenin-mutated HCC (related to Figure 5).

(a) Representative tiled images of H&E and glutamine synthetase (GS) staining on β-catenin-Nrf2 (β-N) and β-catenin-Nrf2-POU2F1 (β-N-POU2F1) animals at 10.7-week timepoint. Scale bar indicates magnification.

(b) β-catenin-hMet (β-M) animals were co-injected with either pT3 (empty vector) or POU2F1 plasmid at time of hydrodynamic tail vein injection (HDTVi) and sacrificed at 7.7-weeks post-HDTVi. Created in BioRender. Lehrich, B. (2025) https://BioRender.com/vmg1nb0.

(c) Representative tiled images of H&E and GS staining on β-M and β-catenin-hMet-POU2F1 (β-M-POU2F1) animals at 7.7-week timepoint. Scale bar indicates magnification.

(d) Representative gross liver images of β-M animals co-injected with either pT3 (empty vector) or hPOU2F1.

(e) Liver weights comparing β-M-pT3 (n=4) and β-M-POU2F1 (n=4) animals at 7.7-week timepoint. Data presented as mean with error bars representing standard deviation (SD). P-values calculated by unpaired two-tailed Student's t-test. Exact p-values provided in source data file.

(f) Liver weight/body weight (LW/BW) ratio comparing β-M-pT3 (n=4) and β-M-POU2F1 (n=4) animals at 7.7-week timepoint. Data presented as mean with error bars representing SD. P-values calculated by unpaired two-tailed Student's t-test. Exact p-values provided in source data file.

(g) Principal component analysis of bulk RNA-sequencing transcriptomic profiles of β-M and β-M-POU2F1 animals, using all genes (n=3 per condition).

(h) Volcano plot highlighting differential expression of genes in mutated-β-catenin gene signature (MBGS) and human POU2F1 gene (POU2F1h) comparing β-M-pT3 (n=3) and β-M-POU2F1 (n=3) animals at 7.7-week timepoint.

(i) Ingeneuity pathway analysis demonstrating pathways downregulated by -log10(p-value) value and p-value comparing β-M-pT3 (n=3) and β-M-POU2F1 (n=3) animals at 7.7-week timepoint. Pathways also colored by z-score. Source data for this figure provided in source data file.

Supplementary Figure 20

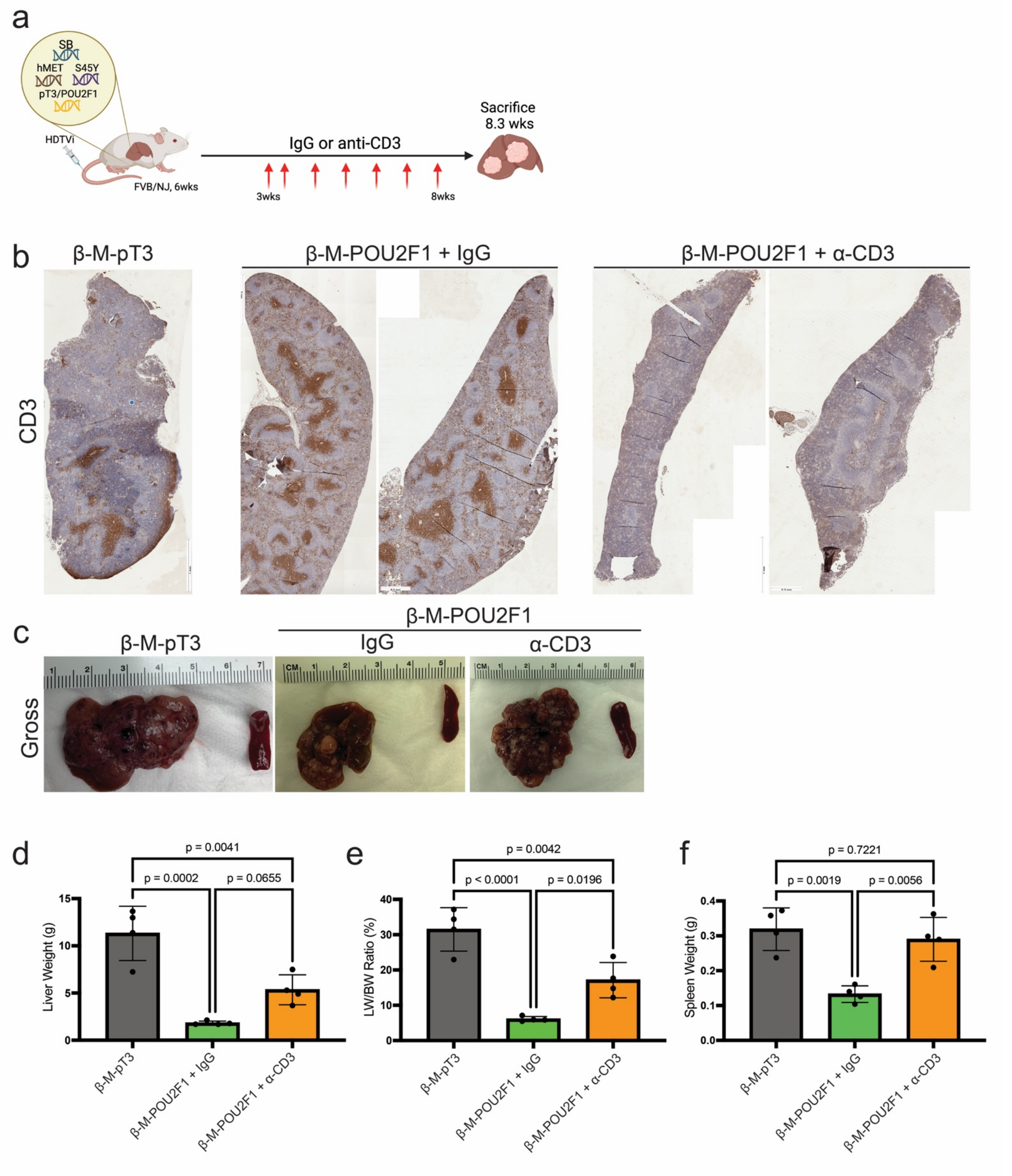

Supplementary Figure 20. Effects of POU2F1 overexpression in CTNNB1-mutated hepatocellular carcinoma are immune-dependent (related to Figure 5).

(a) β-catenin-hMet (β-M) animals were co-injected with either pT3 (empty vector) or POU2F1 plasmid at time of hydrodynamic tail vein injection (HDTVi) and β-M-POU2F1 animals were administered IgG control antibody or αCD3 antibody, and sacrificed at 8.3-weeks post-HDTVi. Created in BioRender. Lehrich, B. (2025) https://BioRender.com/1ciaxx9.

(b) Representative tiled images of CD3 staining on β-M-pT3, β-M-POU2F1 + IgG, and β-M-POU2F1 + α-CD3 at 8.3-week timepoint. Scale bar indicates magnification.

(c) Representative gross liver images of β-M animals co-injected with either pT3 (empty vector) or POU2F1 and administered either IgG or αCD3 antibody at 8.3-weeks post-HDTVi timepoint.

(d) Liver weight (g) comparing β-M-pT3 (n=4), β-M-POU2F1 + IgG, (n=4), and β-M-POU2F1 + α-CD3 animals at 8.3-weeks post-HDTVi timepoint. Data presented as mean with error bars representing standard deviation (SD). P-values calculated via one-way ANOVA with Tukey-HSD post-hoc correction. Exact p-values provided in source data file.

(e) Liver weight/body weight (LW/BW) ratio comparing β-M-pT3 (n=4), β-M-POU2F1 + IgG, (n=4), and β-M-POU2F1 + α-CD3 animals at 8.3-weeks post-HDTVi timepoint. Data presented as mean with error bars representing SD. P-values calculated via one-way ANOVA with Tukey-HSD post-hoc correction. Exact p-values provided in source data file.

(f) Spleen weight (g) comparing β-M-pT3 (n=4), β-M-POU2F1 + IgG, (n=4), and β-M-POU2F1 + α-CD3 animals at 8.3-weeks post-HDTVi timepoint. Data presented as mean with error bars representing SD. P-values calculated via one-way ANOVA with Tukey-HSD post-hoc correction. Exact p-values provided in source data file.

Supplementary Figure 21

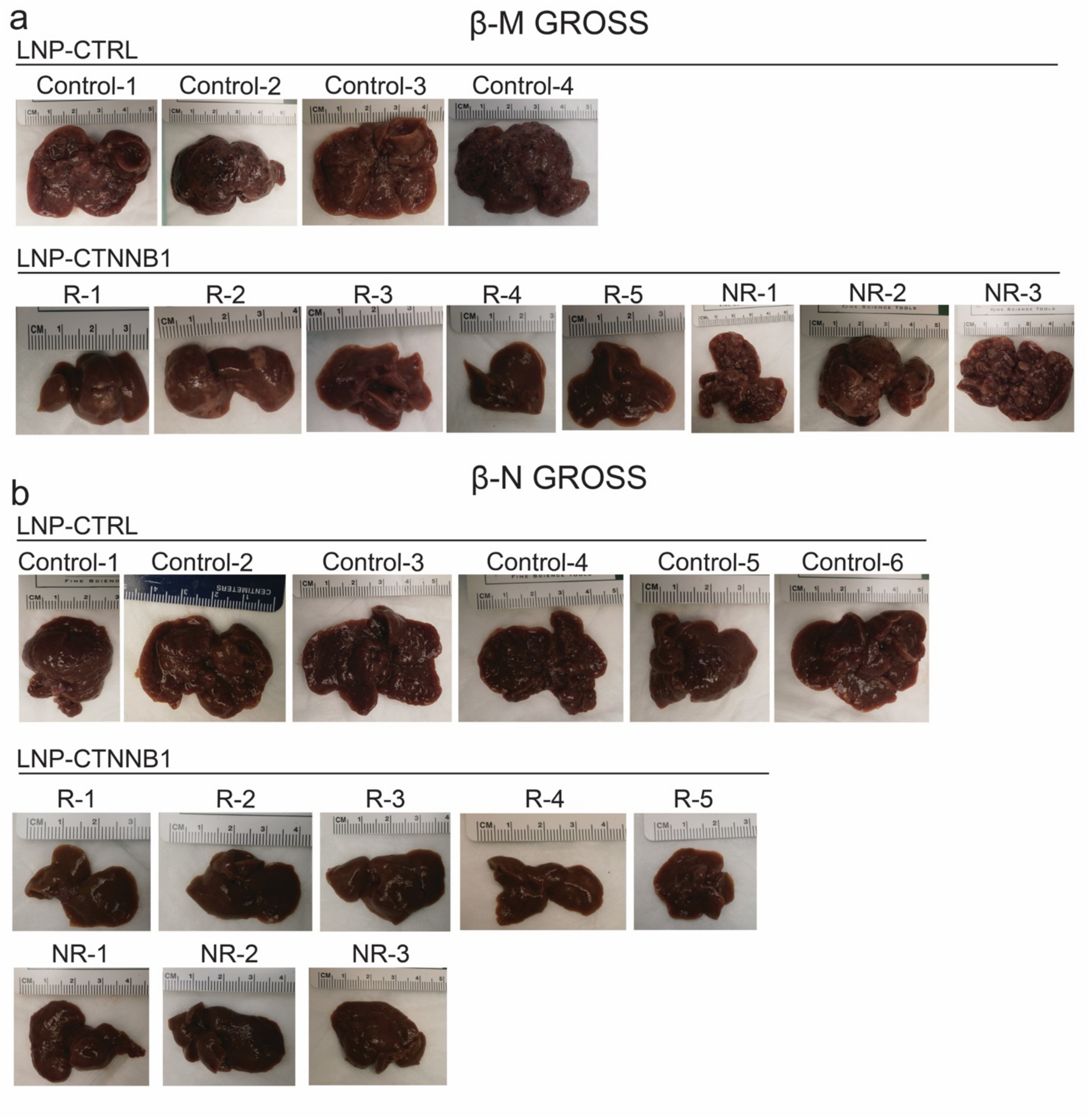

Supplementary Figure 21. Heterogenous response to LNP-CTNNB1 in β-catenin-hMet (β-M) and β-catenin-Nrf2 (β-N) models in advanced-stage disease setting (related to Figure 6).

(a) Gross liver images of LNP-CTRL and LNP-CTNNB1 treated β-M animals from the study at 8.5-week and 10.5-week post-HDTVi, respectively. Animals are designated by "Control" or "NR" (non-responder) or "R" (responder) status. These images are of gross livers shown from experiment in Figure 6a. There is overlap in represented gross livers from animals in Figure 6b.

(b) Gross liver images of LNP-CTRL and LNP-CTNNB1 treated β-N animals from the study at 10.5-week and 13.5-weeks post-HDTVi, respectively. Animals are designated by "Control" or "NR" (non-responder) or "R" (responder) status. These images are of gross livers shown from experiment in Figure 6d. There is overlap in represented gross livers from animals in Figure 6e.

Supplementary Figure 22

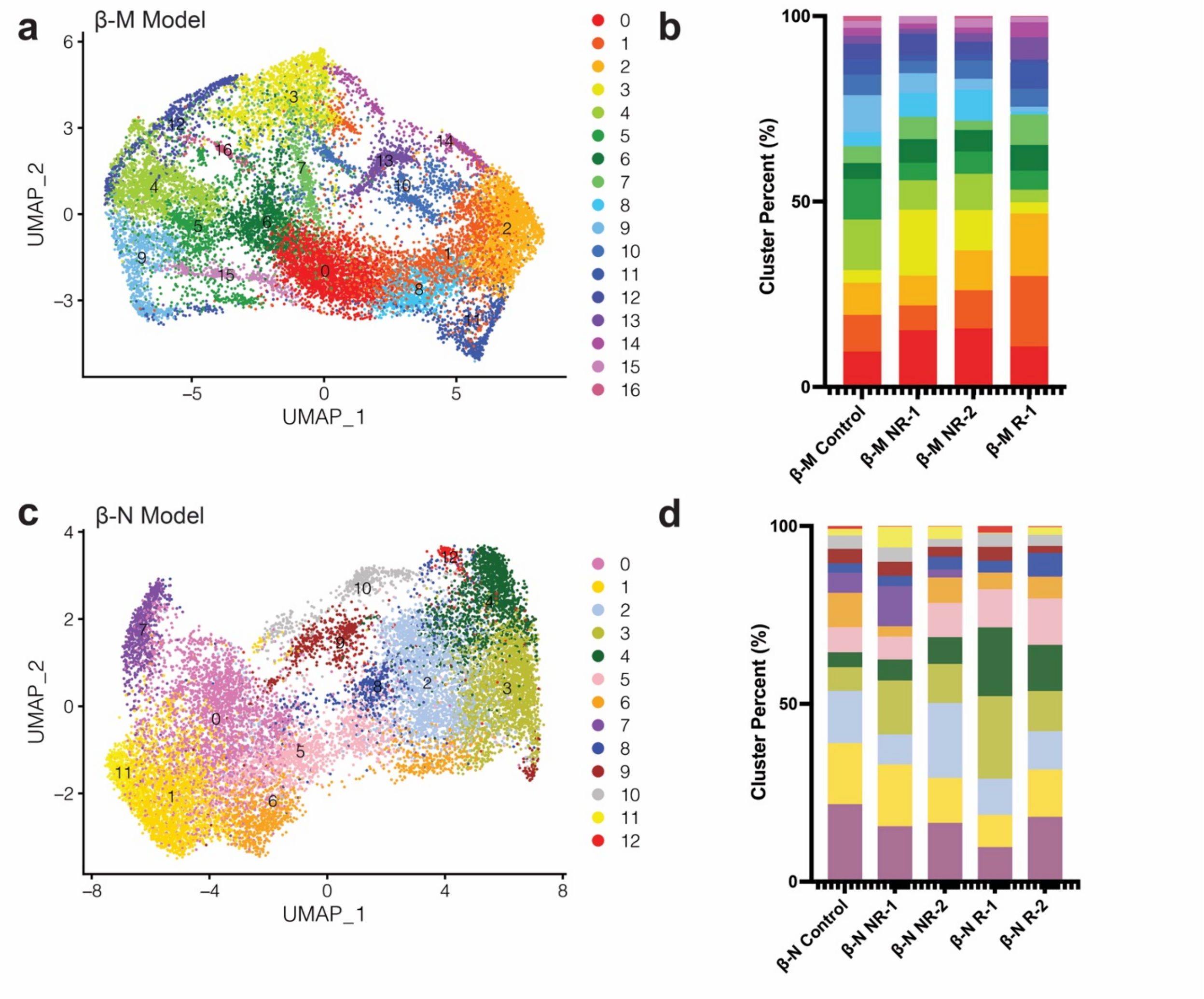

Supplementary Figure 22. 10X Visium spatial transcriptomics on β-catenin-hMet (β-M) and β-catenin-Nrf2 (β-N) models in advanced-stage disease setting reveals enrichment of various clusters based on LNP-CTNNB1 treatment response (related to Figure 6).

(a) Uniform manifold approximation and projection (UMAP) visualization of spot clusters from 10X Visium spatial transcriptomics of the integrated dataset across the β-M model samples. Each color indicates one of the 17 different clusters. UMAP represents 17,685 spots across the 4 slides.

(b) Stacked bar plot of cluster proportions split by treatment condition for β-M Control, β-M NR-1, β-M NR-2, and β-M R-1. There are 4,461 spots in β-M Control, 4,331 spots in β-M NR-1, 4,842 spots in β-M NR-2, and 4,051 spots in β-M R-1. Cluster proportions by LNP treatment response in source data file.

(c) UMAP visualization of spot clusters from 10X Visium spatial transcriptomics of the integrated dataset across the β-N model samples. Each color indicates one of the 13 different clusters. UMAP represents 17,130 spots across the 4 slides.

(d) Stacked bar plot split by treatment condition for β-N Control, β-N NR-1, β-N NR-2, β-N R-1, and β-N R-2. There are 3,884 spots in β-N Control, 3,624 spots in β-N NR-1, 4,037 spots in β-N NR-2, 3,390 spots in β-N R-1, and 2,195 spots in β-N R-2. Cluster proportions by LNP treatment response in source data file.

Supplementary Figure 23

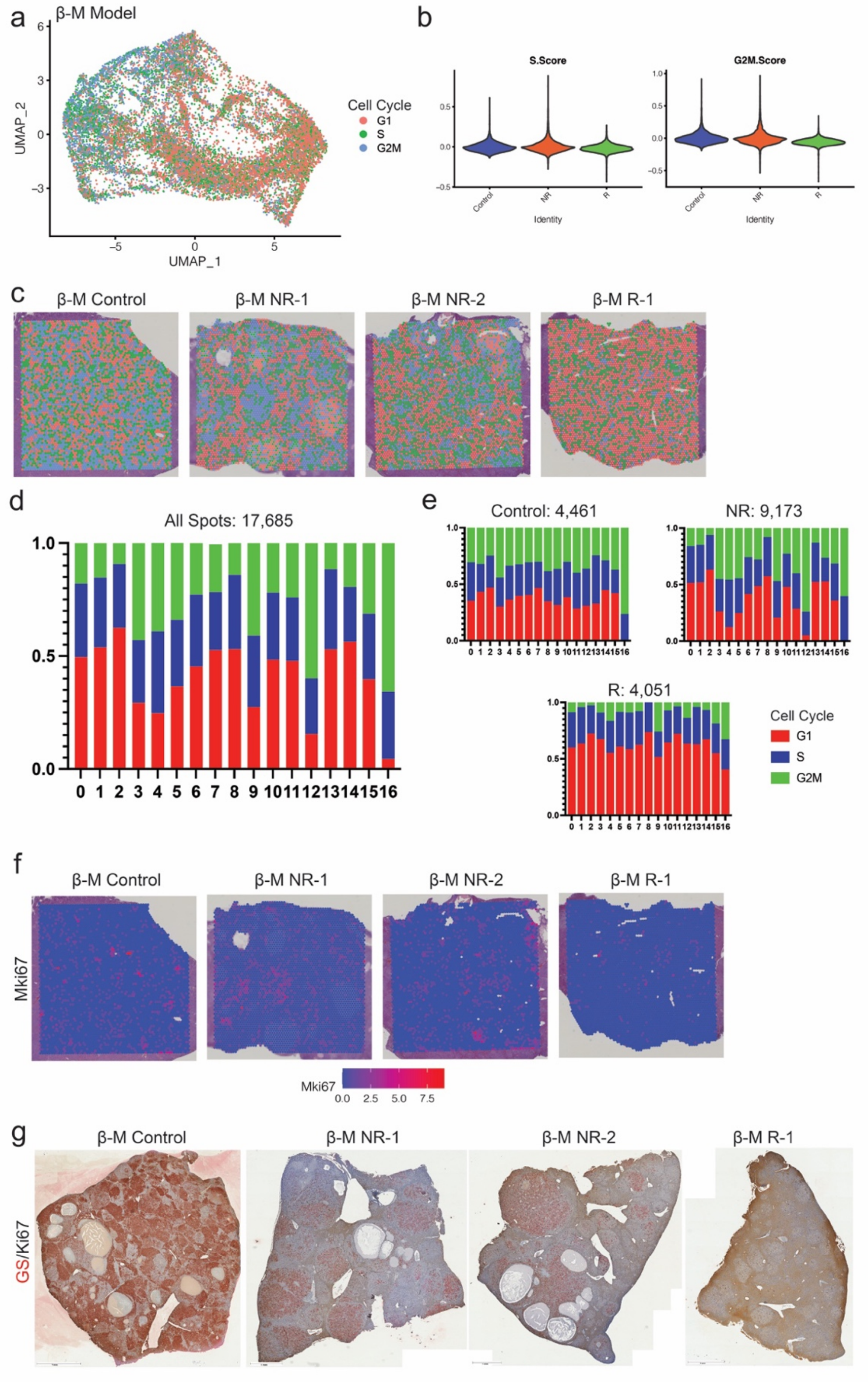

Supplementary Figure 23. 10X Visium spatial transcriptomics on β-catenin-hMet (β-M) model in advanced-stage disease setting reveals decreased cell proliferation following LNP-CTNNB1 treatment (related to Figure 6).

(a) Uniform manifold approximation and projection (UMAP) visualization of individual clusters belonging to either G1, S, or G2M phases of the cell cycle. G1 is colored, S colored blue, and G2M colored green.
(b) Violin plots demonstrating expression of S and G2M module scores across β-M Control, β-M NR, and β-M R animals.
(c) Spatial plots of individual spots belonging to either G1, S, or G2M phases of the cell cycle. G1 is colored, S colored blue, and G2M colored green.
(d) Stacked bar blot demonstrating the proportion of each cluster belonging to either G1, S, or G2M phases of the cell cycle for all cells across all conditions. G1 is colored, S colored blue, and G2M colored green. Cell cycle phase proportions provided in source data file.
(e) Stacked bar blot demonstrating the proportion of each cluster belonging to either G1, S, or G2M phases of the cell cycle for each condition: β-M Control, β-M NR, and β-M R. G1 is colored, S colored blue, and G2M colored green. Cell cycle phase proportions provided in source data file.
(f) Spatial plots of Mki67 expression across the 4 slides. Normalized expression is scaled across each slide.
(g) Representative tiled images of immunohistochemistry (IHC) for glutamine synthetase (GS)/Ki67 comparing LNP-CTRL and LNP-CTNNB1 treated β-M animals (NR and R) at 8.5-week and 10.5-week timepoint, respectively. Animals are designated by “Control” or “NR” (non-responder) or “R” (responder) status. Scale bar indicates magnification.

Supplementary Figure 24

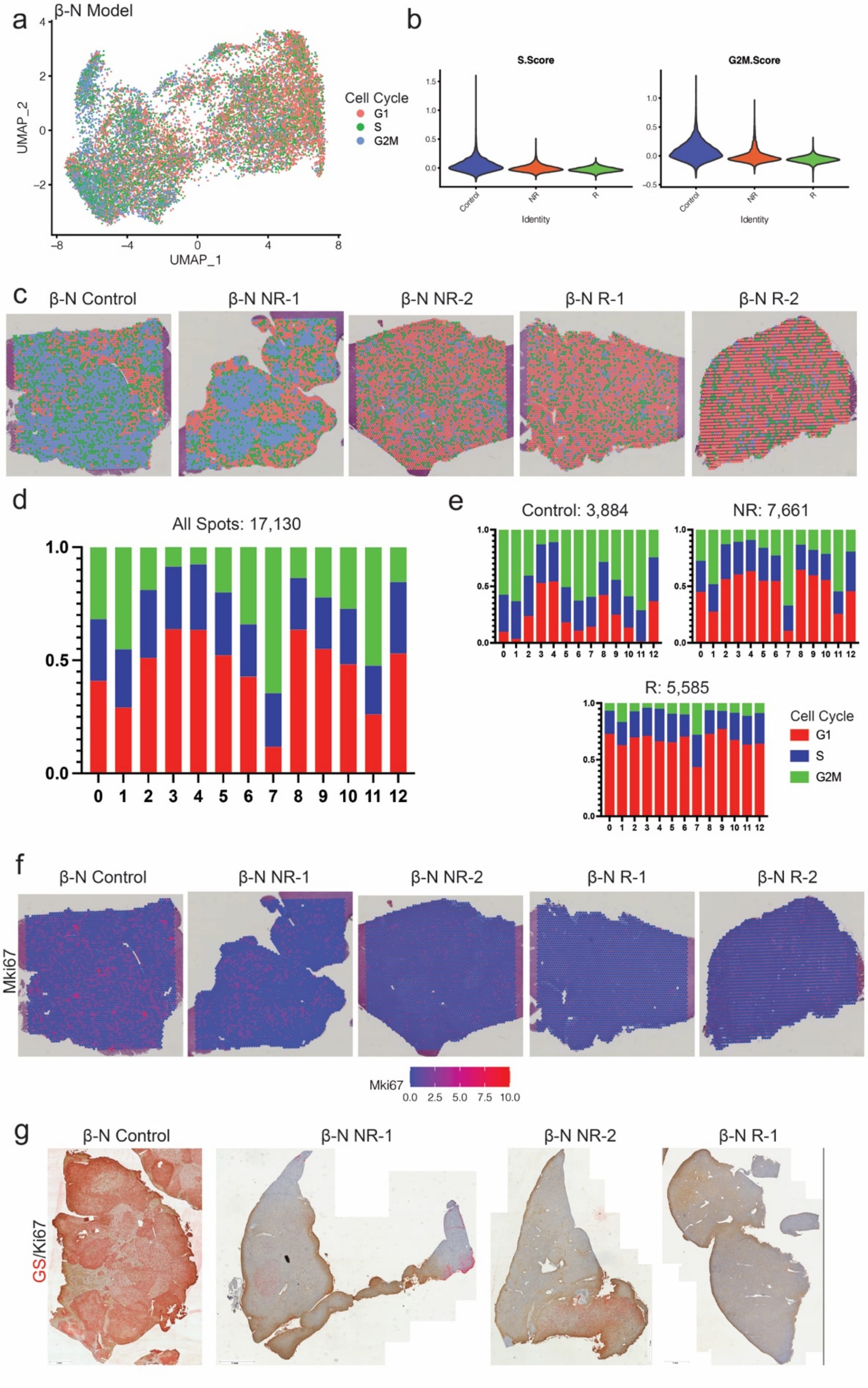

Supplementary Figure 24. 10X Visium spatial transcriptomics on β-catenin-Nrf2 (β-N) model in advanced-stage disease setting reveals decreased cell proliferation following LNP-CTNNB1 treatment (related to Figure 6).

(a) Uniform manifold approximation and projection (UMAP) visualization of individual clusters belonging to either G1, S, or G2M phases of the cell cycle. G1 is colored, S colored blue, and G2M colored green.
(b) Violin plots demonstrating expression of S and G2M module scores across β-N Control, β-N NR, and β-N R animals.
(c) Spatial plots of individual spots belonging to either G1, S, or G2M phases of the cell cycle. G1 is colored, S colored blue, and G2M colored green.
(d) Stacked bar blot demonstrating the proportion of each cluster belonging to either G1, S, or G2M phases of the cell cycle for all cells across all conditions. G1 is colored, S colored blue, and G2M colored green. Cell cycle phase proportions provided in source data file.
(e) Stacked bar blot demonstrating the proportion of each cluster belonging to either G1, S, or G2M phases of the cell cycle for each condition: β-N Control, β-N NR, and β-N R. G1 is colored, S colored blue, and G2M colored green. Cell cycle phase proportions provided in source data file.
(f) Spatial plots of Mki67 expression across the 4 slides. Normalized expression is scaled across each slide.
(g) Representative tiled images of IHC for glutamine synthetase (GS)/Ki67 comparing LNP-CTRL and LNP-CTNNB1 treated β-N animals (NR and R) at 10.5-week and 13.5-week timepoint, respectively. Animals are designated by “Control” or “NR” (non-responder) or “R” (responder) status. Scale bar indicates magnification.

Supplementary Figure 25

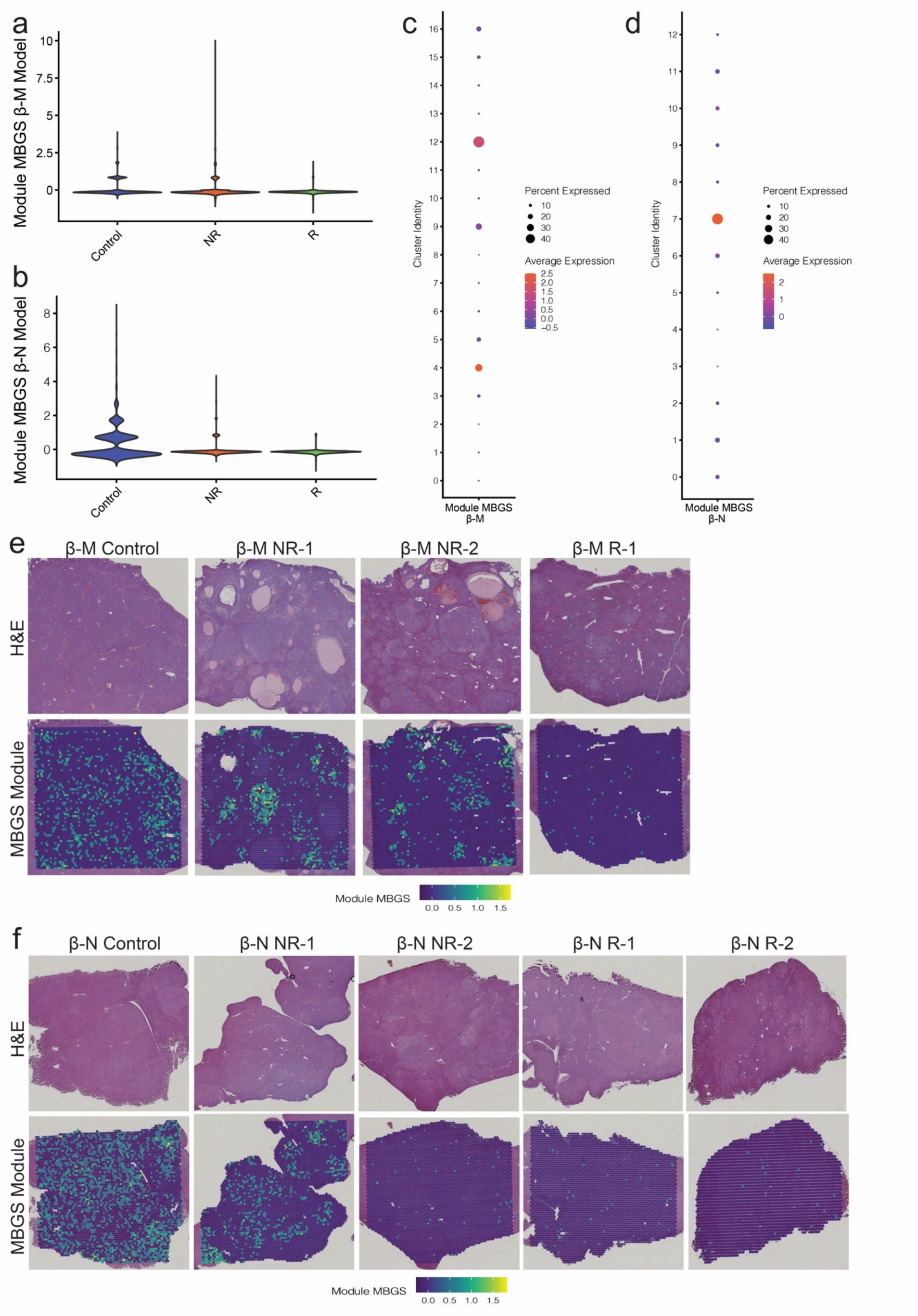

Supplementary Figure 25. 10X Visium spatial transcriptomics on β-catenin-hMet (β-M) and β-catenin-Nrf2 (β-N) models in advanced-stage disease setting reveals tumor nodules which are mutated-β-catenin gene signature (MBGS)-high are actively proliferating (related to Figure 6).

(a) Violin plot demonstrating expression of MBGS module score across β-M Control, NR, and R. There is graded decrease in expression of MBGS module score from β-M Control to NR to R.
(b) Violin plot demonstrating expression of MBGS module score across β-N Control, NR, and R. There is graded decrease in expression of MBGS module score from β-N Control to NR to R.
(c) Dot plot demonstrating expression of MBGS module score across the different clusters in β-M model. Highest expression in clusters 4, 9, and 12.
(d) Dot plot demonstrating expression of MBGS module score across the different clusters in β-N model. Highest expression in cluster 7.
(e) Spatial plot of MBGS module score across the β-M Control, NR, and R. MBGS module score has normalized expression across the 4 slides.
(f) Spatial plot of MBGS module score across the β-N Control, NR, and R. MBGS module score has normalized expression across the 5 slides.

Supplementary Figure 26

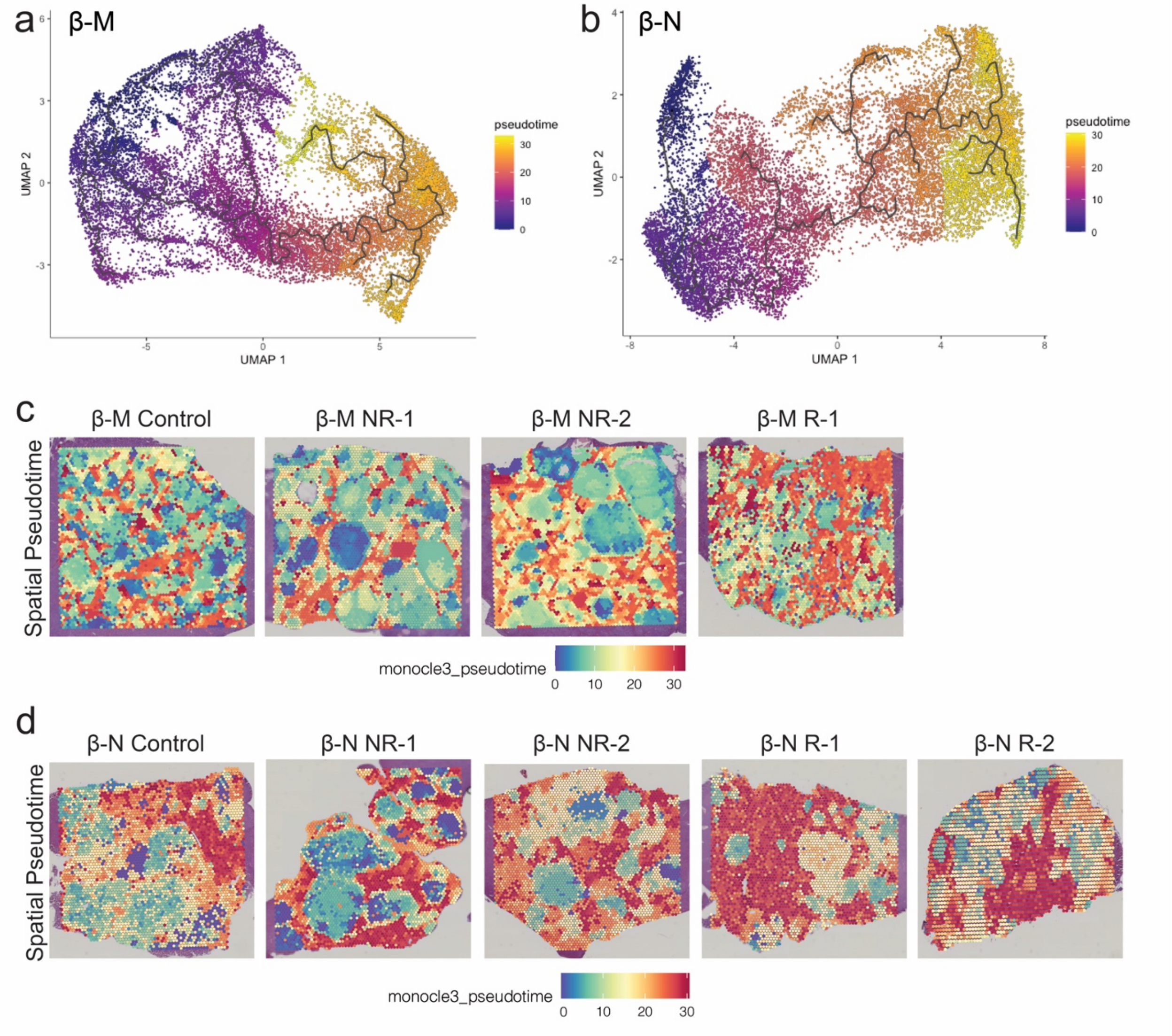

Supplementary Figure 26. Spatial pseudotime analysis on 10X Visium spatial transcriptomics on β-catenin-hMet (β-M) and β-catenin-Nrf2 (β-N) models in advanced-stage disease setting reveals incomplete tumor reprogramming in MBGS-/Mki67-low nodules (related to Figure 6).

(a) Uniform manifold approximation and projection (UMAP) visualization of pseudotime for the β-M model.
(b) Uniform manifold approximation and projection (UMAP) visualization of pseudotime for the β-N model.
(c) Spatial plots of pseudotime for the β-M model: Control, NR-1, NR-2, and R-1.
(d) Spatial plots of pseudotime for the β-N model: Control, NR-1, NR-2, R-1, and R-2.

Supplementary Figure 27

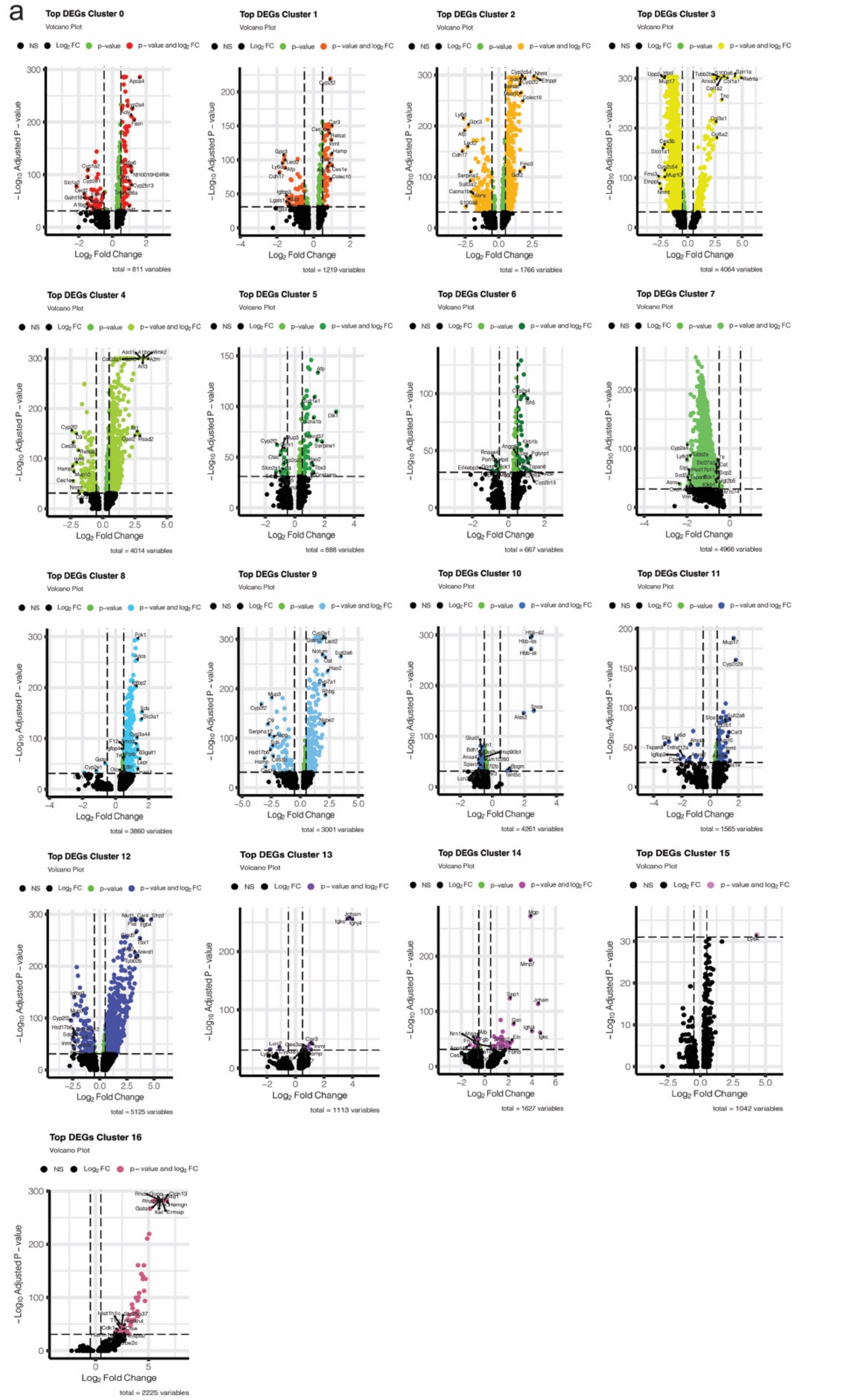

Supplementary Figure 27. Differential gene expression analysis by each cluster in the β-catenin-hMet (β-M) model from 10X Visium spatial transcriptomics (related to Figure 6).

(a) Volcano plot highlighting selected differentially expressed genes (DEGs) in each of the 17 different clusters from the 10X Visium spatial transcriptomics dataset comparing the DEGs in each cluster to all other clusters.

Supplementary Figure 28

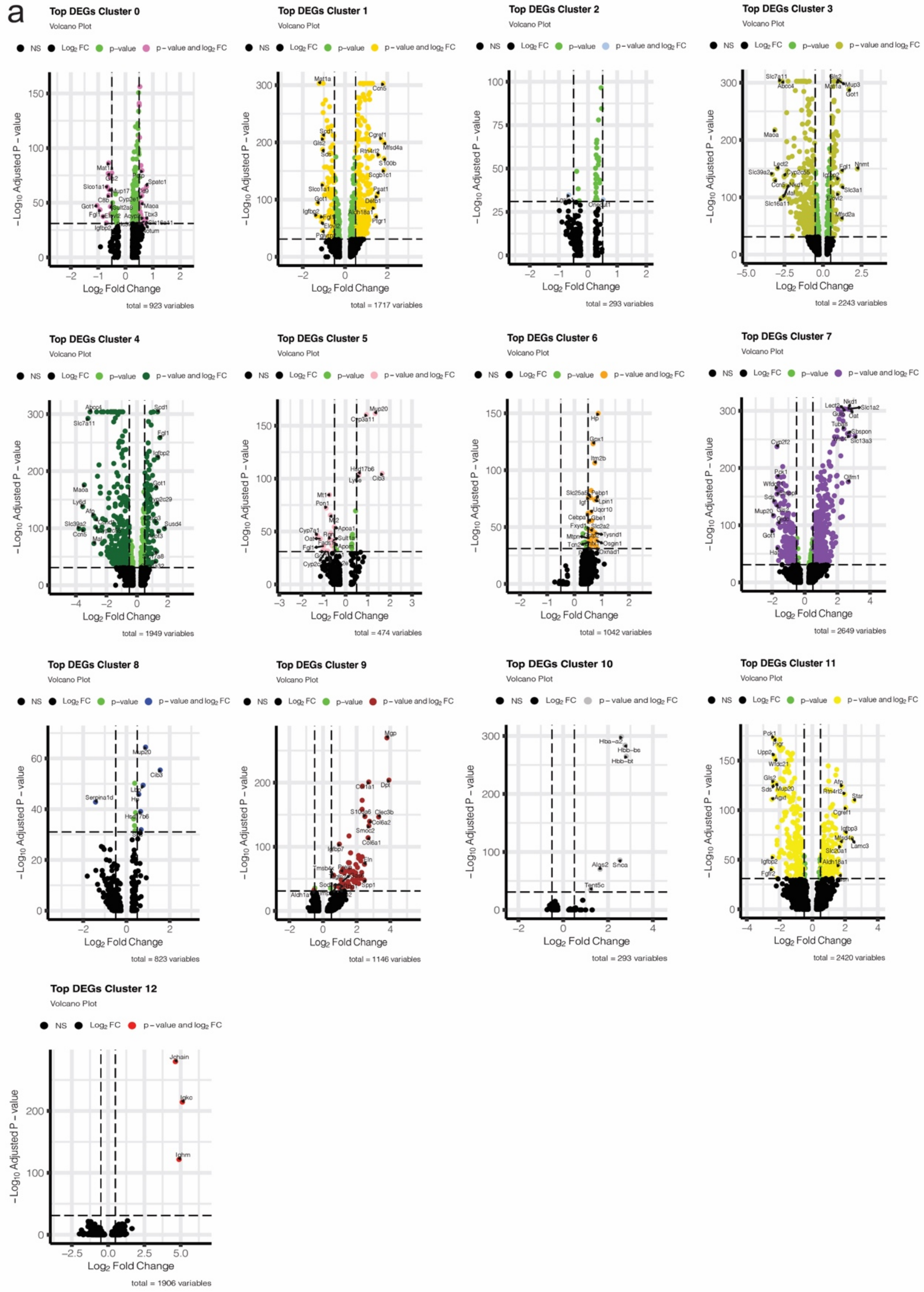

Supplementary Figure 28. Differential gene expression analysis by each cluster in the β-catenin-Nrf2 (β-N) model from 10X Visium spatial transcriptomics (related to Figure 6).

(a) Volcano plot highlighting selected differentially expressed genes (DEGs) in each of the 13 different clusters from the 10X Visium spatial transcriptomics dataset comparing the DEGs in each cluster to all other clusters.

Supplementary Figure 29

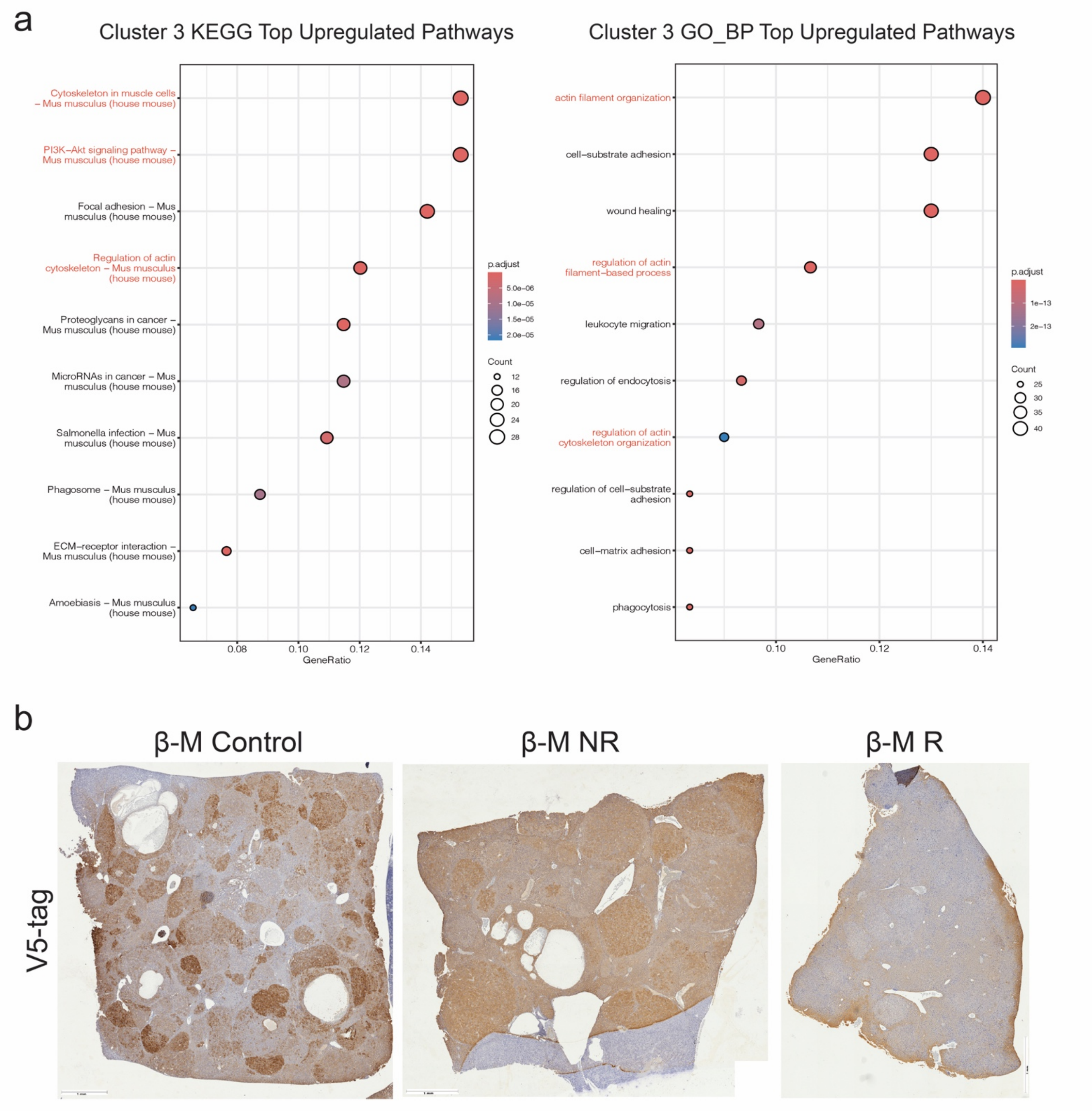

Supplementary Figure 29. Pathway enrichment analysis on cluster 3 in β-catenin-hMet (β-M) model from 10X Visium spatial transcriptomics reveals enrichment of MET signaling pathway. (related to Figure 6).

(a) (Left) Kyoto encyclopedia of genes and genomes (KEGG) pathway enrichment analysis represented as dot plot for top upregulated pathways in cluster 3 of β-M model. Significant differentially expressed upregulated genes defined by min.pct = 0.25, log2FC > 0.25, and p_adjusted <0.05. Significant pathways displayed defined by p_adjusted <0.05. (Right) Gene ontology (GO) pathway enrichment analysis represented as dot plot for top upregulated pathways in cluster 3 of β-M model. Significant differentially expressed upregulated genes defined by min.pct = 0.25, log2FC > 0.25, and p_adjusted <0.05. Significant pathways displayed defined by p_adjusted <0.05.

(b) Representative tiled images of IHC for V5-tag (present on the hMet plasmid) comparing LNP-CTRL and LNP-CTNNB1 treated β-M animals (NR and R) at 8.5-week and 10.5-week timepoint, respectively. Animals are designated by "Control" or "NR" (non-responder) or "R" (responder) status. Scale bar indicates magnification.

Supplementary Figure 30

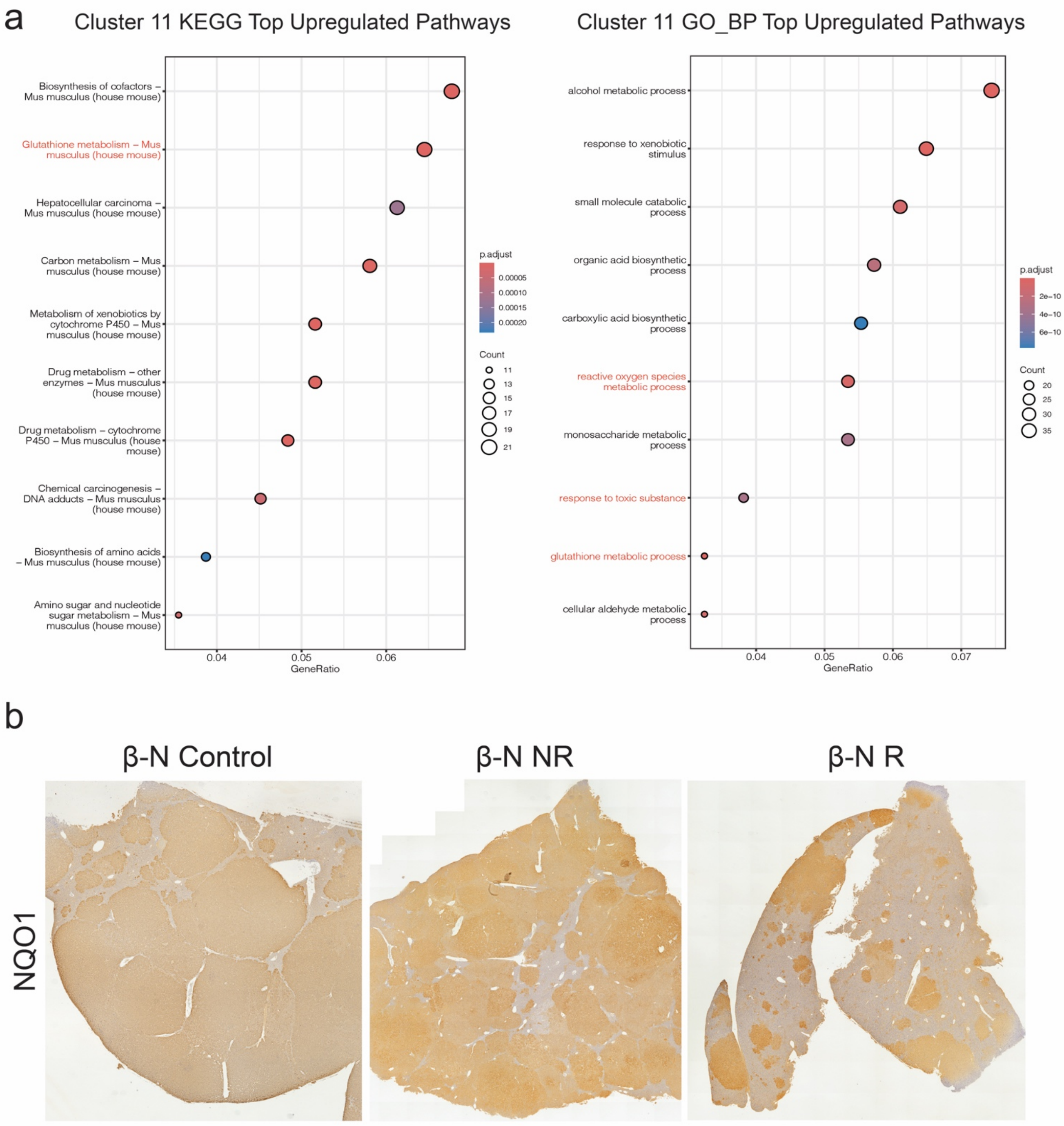

Supplementary Figure 30. Pathway enrichment analysis on cluster 11 in β-catenin-Nrf2 (β-N) model from 10X Visium spatial transcriptomics reveals enrichment of NRF2 signaling pathway. (related to Figure 6).

(a) (Left) Kyoto encyclopedia of genes and genomes (KEGG) pathway enrichment analysis represented as dot plot for top upregulated pathways in cluster 11 of β-N model. Significant differentially expressed upregulated genes defined by min.pct = 0.25, log2FC > 0.25, and p_adjusted <0.05. Significant pathways displayed defined by p_adjusted <0.05. (Right) Gene ontology (GO) pathway enrichment analysis represented as dot plot for top upregulated pathways in cluster 11 of β-N model. Significant differentially expressed upregulated genes defined by min.pct = 0.25, log2FC > 0.25, and p_adjusted <0.05. Significant pathways displayed defined by p_adjusted <0.05.

(b) Representative tiled images of IHC for NQO1 (downstream of NRF2 activation) comparing LNP-CTRL and LNP-CTNNB1 treated β-N animals (NR and R) at 10.5-week and 13.5-week timepoint, respectively. Animals are designated by "Control" or "NR" (non-responder) or "R" (responder) status. Scale bar indicates magnification.

Supplementary Figure 31

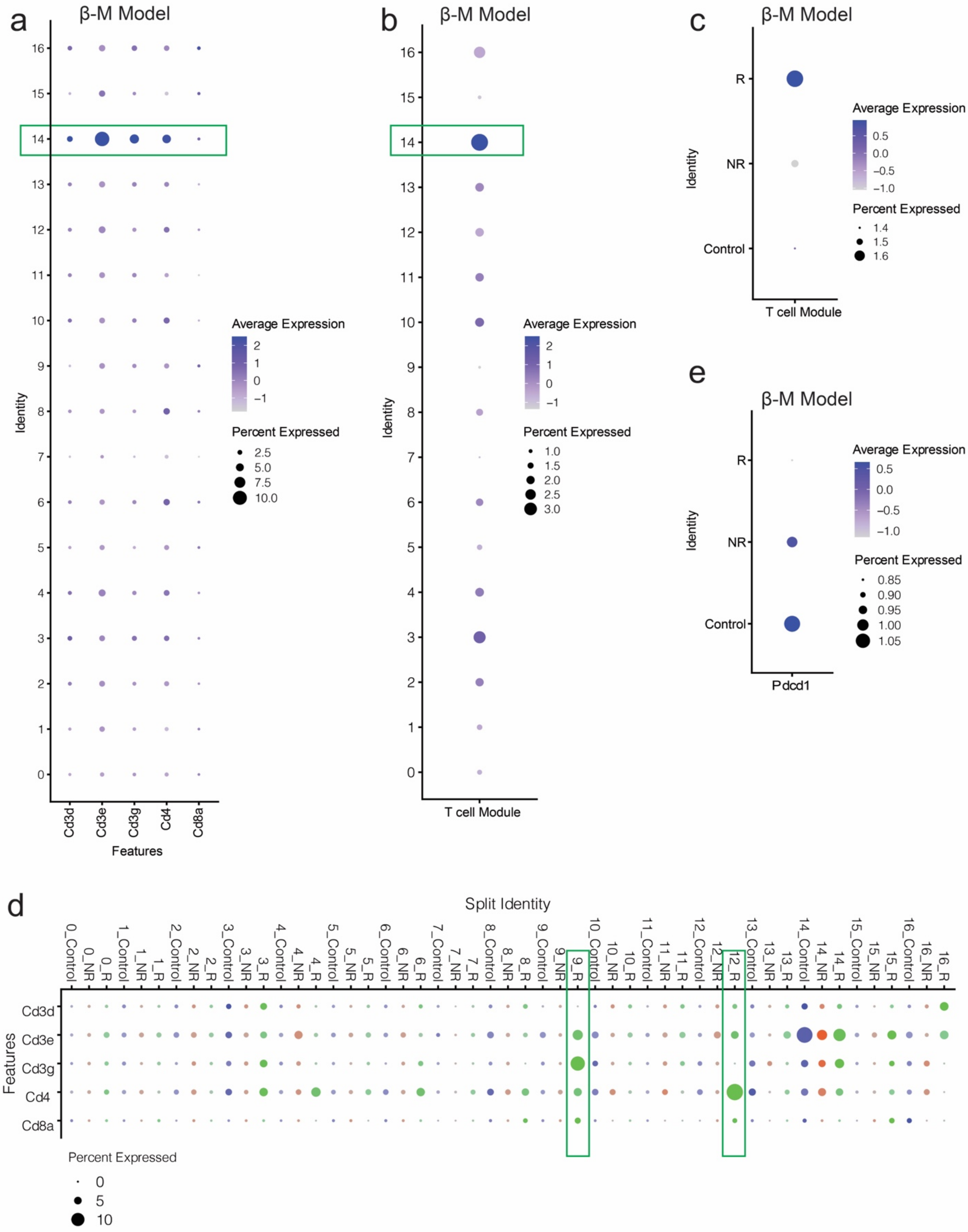

Supplementary Figure 31. 10X Visium spatial transcriptomics on β-catenin-hMet (β-M) model in late-stage disease setting reveals active T cell infiltration (related to Figure 7).

(a) Dot plot showing expression by cluster for various T cell marker genes: *Cd3d, Cd3e, Cd3g, Cd4, Cd8a* using the 10X Visium spatial transcriptomics data from the β-M model.
(b) Dot plot showing expression by cluster for T cell module score based on composite expression of T cell marker genes: *Cd3d, Cd3e, Cd3g, Cd4, Cd8a* using the 10X Visium spatial transcriptomics data from the β-M model.
(c) Dot plot showing expression by treatment response for T cell module score based on composite expression of T cell marker genes: *Cd3d, Cd3e, Cd3g, Cd4, Cd8a* using the 10X Visium spatial transcriptomics data from the β-M model.
(d) Dot plot showing expression by cluster and by treatment response for various T cell marker genes: *Cd3d, Cd3e, Cd3g, Cd4, Cd8a* using the 10X Visium spatial transcriptomics data from the β-M model.
(e) Dot plot showing expression by treatment response for *Pdcd1* using the 10X Visium spatial transcriptomics data from the β-M model.

Supplementary Figure 32

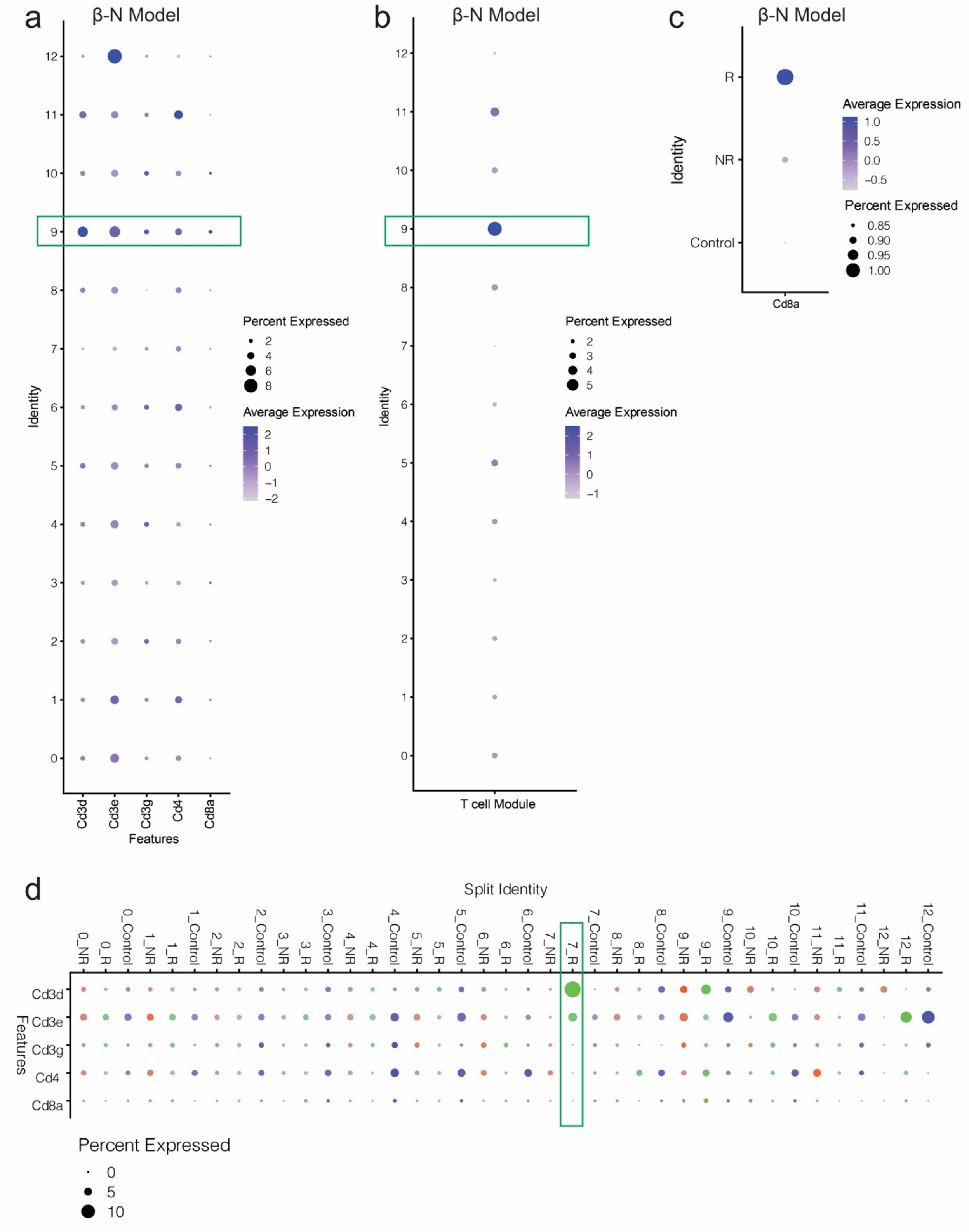

Supplementary Figure 32. 10X Visium spatial transcriptomics on β-catenin-Nrf2 (β-N) model in late-stage disease setting reveals active T cell infiltration (related to Figure 7).

(a) Dot plot showing expression by cluster for various T cell marker genes: *Cd3d, Cd3e, Cd3g, Cd4, Cd8a* using the 10X Visium spatial transcriptomics data from the β-N model.
(b) Dot plot showing expression by cluster for T cell module score based on composite expression of T cell marker genes: *Cd3d, Cd3e, Cd3g, Cd4, Cd8a* using the 10X Visium spatial transcriptomics data from the β-N model.
(c) Dot plot showing expression by treatment response for *Cd8a* using the 10X Visium spatial transcriptomics data from the β-N model.
(d) Dot plot showing expression by cluster and by treatment response for various T cell marker genes: *Cd3d, Cd3e, Cd3g, Cd4, Cd8a* using the 10X Visium spatial transcriptomics data from the β-N model.

Supplementary Figure 33

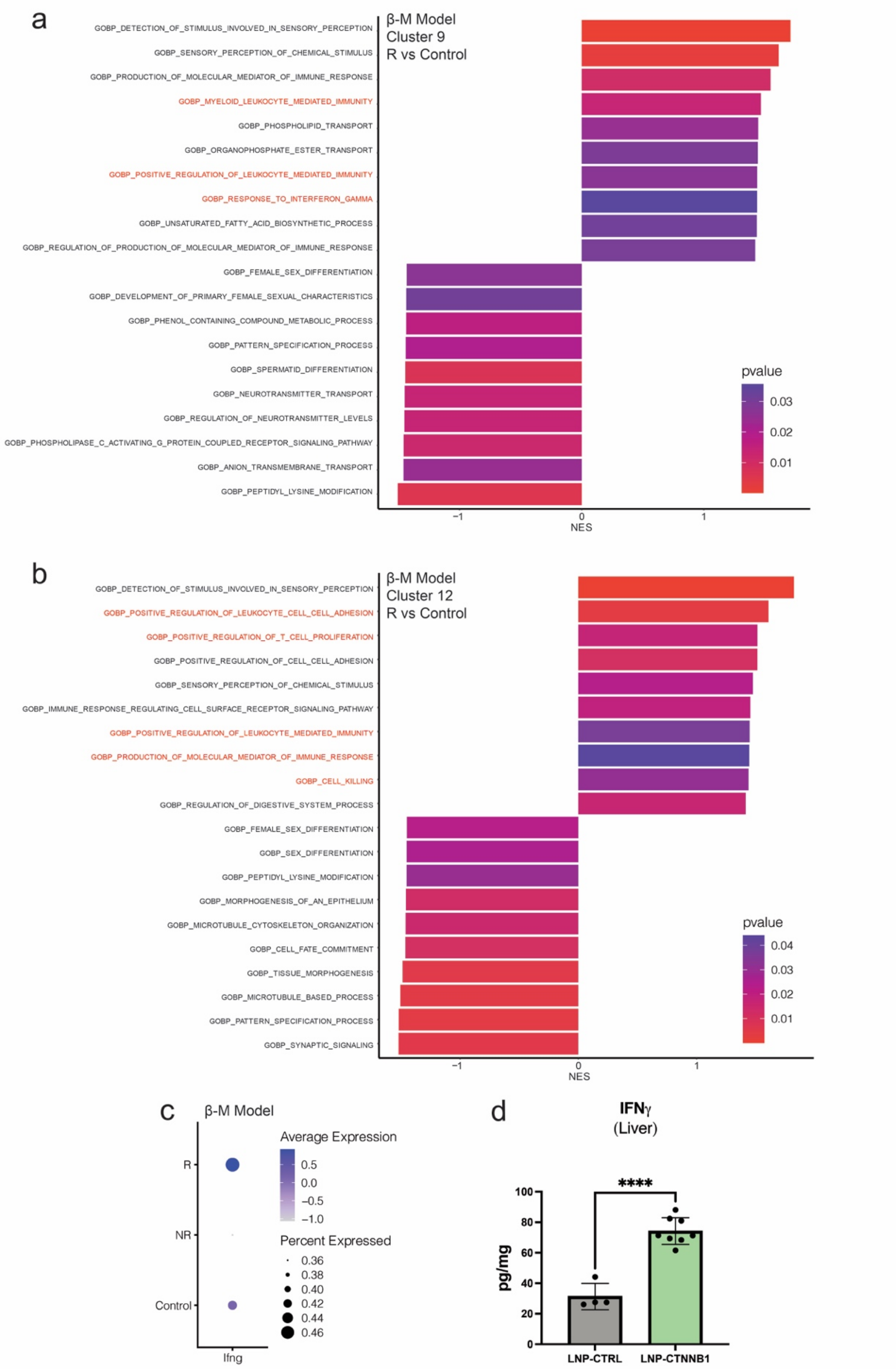

Supplementary Figure 33. Gene set enrichment analysis with gene ontology pathways from 10X Visium spatial transcriptomics on β-catenin-hMet (β-M) model in late-stage disease setting (related to Figure 7).

(a) Waterfall plot of gene set enrichment analysis (GSEA) with gene ontology biological processes (GOBP) pathways of cluster 9 comparing β-M R (LNP-CTNNB1; responder) to β-M Control (LNP-CTRL). Top 10 up and down pathways displayed based on normalized enrichment score (NES) and p-value.

(b) Waterfall plot GSEA with gene ontology biological processes (GOBP) pathways of cluster 12 comparing β-M R (LNP-CTNNB1; responder) to β-M Control (LNP-CTRL). Top 10 up and down pathways displayed based on normalized enrichment score (NES) and p-value.

(c) Dot plot showing expression by treatment response for *Ifng* using the 10X Visium spatial transcriptomics data from the β-M model.

(d) ELISA for IFNγ in LNP-CTRL (n=4) and LNP-CTNNB1 (n=8; 1mg/kg) treated β-catenin-Nrf2 (β-N) animals. Data presented as mean with error bars representing standard deviation (SD). P-values calculated by unpaired two-tailed Student's t-test. Exact p-value provided in source data file.

Supplementary Figure 34

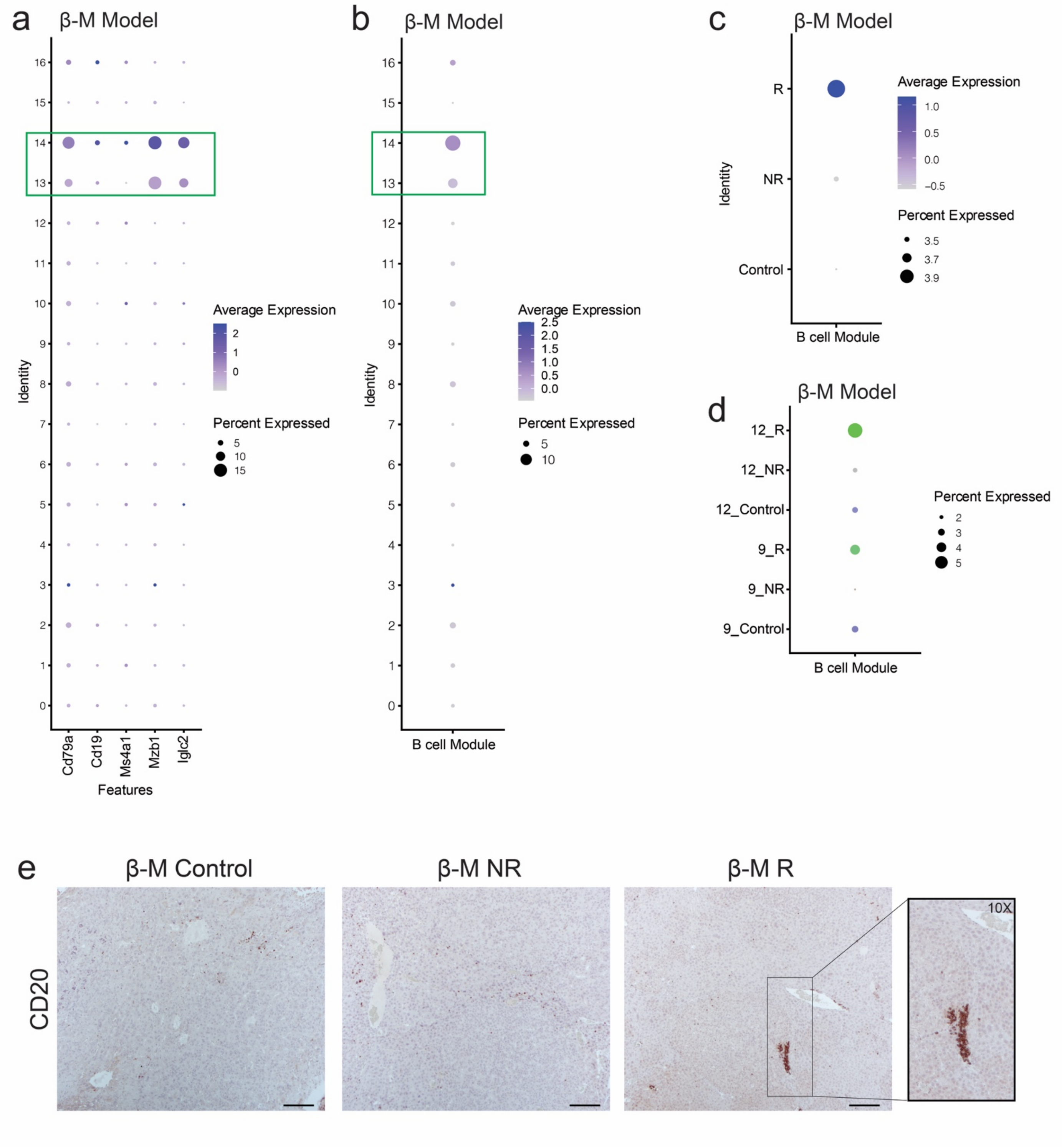

Supplementary Figure 34. 10X Visium spatial transcriptomics on β-catenin-hMet (β-M) model in late-stage disease setting reveals active B cell infiltration (related to Figure 7).

(a) Dot plot showing expression by cluster for various B cell marker genes: *Cd79a, Cd19, Ms4a1, Mzb1,* and *Iglc2* using the 10X Visium spatial transcriptomics data from the β-M model.

(b) Dot plot showing expression by cluster for B cell module score based on composite expression of B cell marker genes: *Cd79a, Cd19, Ms4a1, Mzb1, Igkc, Ighm, Jchain* using the 10X Visium spatial transcriptomics data from the β-M model.

(c) Dot plot showing expression by LNP treatment response for B cell module score using the 10X Visium spatial transcriptomics data from the β-M model.

(d) Dot plot showing expression by cluster and by LNP treatment response for B cell module score using the 10X Visium spatial transcriptomics data from the β-M model.

(e) Representative immunohistochemistry (IHC) images for CD20 across β-M Control, β-M NR, and β-M R LNP-CTNNB1 (1mg/kg) treated animals. IHC repeated at least twice for the indicated CD20 stain in multiple replicate animals. 5x objective magnification. Scale bar indicates 200μm.

Supplementary Figure 35

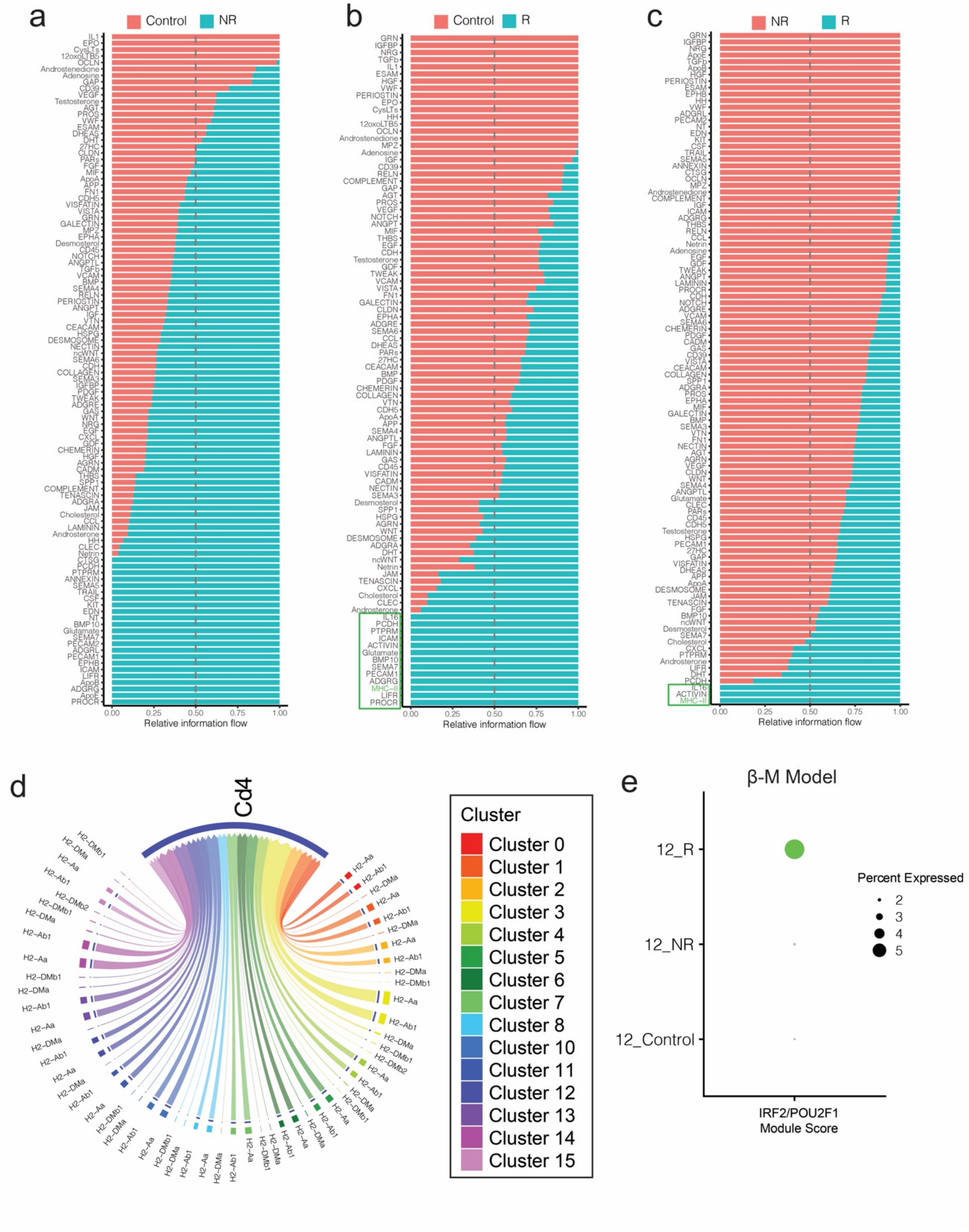

Supplementary Figure 35. Spatial CellChat analysis reveals increased MHC-II signaling in Cluster 12 within LNP-CTNNB1 responders in β-catenin-hMet (β-M) model in late-stage disease (related to Figure 7).

(a) Stacked horizontal bar plot comparing relative information flow from CellChat between β-M Control (LNP-CTRL) to β-M NR (LNP-CTNNB1; non-responder).

(b) Stacked horizontal bar plot comparing relative information flow from CellChat between β-M Control (LNP-CTRL) to β-M R (LNP-CTNNB1; responder). Boxed pathways showing 100% information flow in β-M R animals. MHC-II highlighted in green as 100% enriched in β-M R.

(c) Stacked horizontal bar plot comparing relative information flow from CellChat between β-M NR (LNP-CTNNB1; non-responder) to β-M R (LNP-CTNNB1; responder). Boxed pathways showing 100% information flow in β-M R animals. MHC-II highlighted in green as 100% enriched in β-M R.

(d) CellChat cord diagram for MHC-II pathway in R β-M animals demonstrating information flow from each cluster demonstrating antigen expression to *Cd4* within cluster 12. No information flow observed in Control or NR β-M animals.

(e) Dot plot showing expression by treatment response in cluster 12 for IRF2/POU2F1 target gene module score using the 10X Visium spatial transcriptomics data from the β-M model.

Supplementary Figure 36

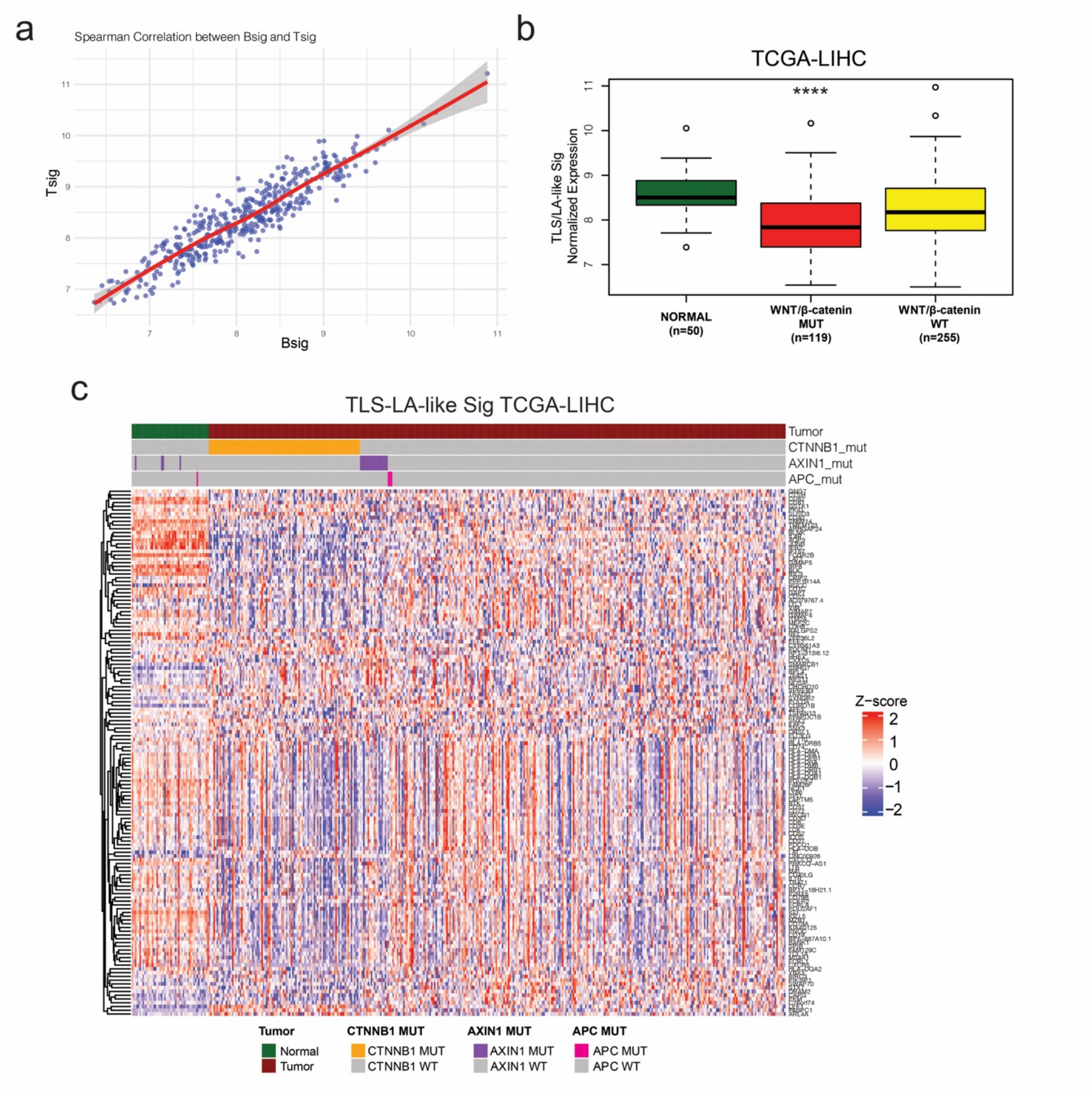

Supplementary Figure 36. Tertiary lymphoid structure (TLS)/Lymhpoid aggregate (LA)-like signature demonstrates decreased expression in WNT/β-catenin active tumors in TCGA-LIHC (related to Figure 8).

(a) Correlation plot between CD4 T cell signature and B cell signature (see Methods) in TCGA-LIHC. Rho=0.9156054, *p*<0.001.

(b) Boxplots of average normalized expression of LA-like gene signature stratified by adjacent normal liver (n=50; green), Wnt/β-catenin mutant (n=119; red), and Wnt/β-catenin wild-type (n=255; yellow). Wnt/β-catenin mutant (n=119) defined by *CTNNB1*-mutated patients (n=98), *AXIN1*-mutated patients (n=18), and *APC*-mutated patients (n=3). The centre line shows the median, the box limits show the interquartile range (IQR; the range between the 25th and 75th percentile) and the whiskers show 1.5 x IQR. P-value calculated by one-way ANOVA with Tukey-HSD post-hoc correction. ****p<0.0001 comparing Wnt/β-catenin mutant vs Wnt/β-catenin wild-type. Exact p-values for all comparisons provided in the source data file.

(c) Heatmap visualization of z-scored expression values of the LA-like signature in TCGA-LIHC patients (n=374; red) and adjacent normal liver (n=50; green). Tumor data is stratified by *CTNNB1*-mutated patients (n=98; gold), *AXIN1*-mutated patients (n=18; purple), and *APC*-mutated patients (n=3; pink).

SUPPLEMENTARY TABLES

Supplementary Table 1: Primary Antibodies and Antigen Retrieval Conditions for Immunohistochemistry (IHC)

Antibody	Catalogue Number	Company	Species	Primary Ab Dilution	Secondary Ab Dilution	Antigen Retrieval Conditions
MYC-tag	2278	Cell Signaling	RbM	1:100	1:250	DAKO, PC, ON
NQO1	376023	Santa Cruz	MsM	1:100	1:250	Cit, PC, ON
V5-tag	14-6796-82	eBioscience	MsM	1:100	1:250	Cit, PC, ON
Glutamine synthetase (GS)	G2781	Sigma-Aldrich	RbP	1:1500	1:1000	NA, no antigen retrieval
Ki67	12202	Cell Signaling	RbM	1:500	1:250	Cit, PC, 1hr RT
Cyclin-D1	Ab134175	Abcam	RbM	1:100	1:250	Tris EDTA, Steamer, ON
CYP2E1	HPA-009128	Sigma	RbP	1:100	1:500	Cit, PC, 1hr RT
OAT	376050	Santa Cruz	MsM	1:100	1:500	Tris EDTA, PC, 1hr RT
HAMP	190775	Abcam	RbM	1:100	1:500	Cit, PC, 1hr RT
ARG1	91279	Abcam	RbP	1:100	1:500	Cit, MW, 1hr RT
CYP2F2	374540	Santa Cruz	MsM	1:100	1:500	Cit, PC, 1hr RT
CD3	Ab16669	Abcam	RbM	1:100	1:500	DAKO, PC, ON
CD4	Ab183685	Abcam	RbM	1:100	1:500	DAKO, PC, ON

CD8	Ab217344	Abcam	RbM	1:100	1:500	DAKO, PC, ON
CD20	Ab236434	Abcam	RbM	1:100	1:500	DAKO, PC, ON
S100A8/9	Ab288715	Abcam	RbM	1:100	1:500	DAKO, PC, ON
CD45	53665	Santa Cruz	RtM	1:100	1:250	DAKO, PC, 1hr RT
GZMB	Ab4059	Abcam	RbP	1:100	1:500	Tris EDTA, PC, ON

Abbreviations: Ab=Antibody; Rb=Rabbit; Ms=Mouse; Rt=Rat; PC=pressure cooker; Cit=Citrate; ON=overnight; M=monoclonal; P=polyclonal; RT=room temperature.

Supplementary Table 2: Sequence of qPCR Primers

Gene	Forward	Reverse
mRn18s	GTAACCCGTTGAACCCCATT	CCATCCAATCGGTAGTAGCG
mCtnnb1	GGGTCCTCTGTGAACTTGCTC	TTCTTGTAATCCTGTGGCTTGTCC
mGlul	CTCGCTCTCCTGACCTGTTC	TTCAAGTGGGAACTTGCTGA
mCcnd1	TTTCTTTCCAGAGTCATCAAGTGT	TGACTCCAGAAGGGCTTCAA
mLect2	GGACGTGTGACAGCTATGGC	TCCCAGTGAATGGTGCATACA
mRgn	GTATGGGAGGAAGCGTCACAGT	CAATGGTGGCAACATAGCCTCC
hPOU2F1	TGGGCTTACTTTGACGCCTG	GTAGGAGGTTGGCTTGGCTT

Supplementary Table 3: qPCR Primers for Mouse Ctnnb1 and Human CTNNB1

Gene	Species	Dye	Assay ID
Ctnnb1	Mouse	FAM	Mm00483039_m1
CTNNB1	Human	FAM	Hs00355045_m1
Gapdh	Mouse	VIC	Mouse GAPDH Control Mix Reference Number 4351309
GAPDH	Human	VIC	GAPDH Control Mix Reference Number 4325792

Abbreviations: *CTNNB1*=gene that encodes β-catenin protein; *GAPDH=glyceraldehyde phosphate dehydrogenase*; FAM=Fluorescein amidite; VIC=VIC phosphoramidite.

Supplementary Table 4: Antibody List for Flow Cytometry and Channels

**Panel**				
Antibody	Channel	Clone	Company	Catalog#
CD45	Alexa 700	I3/2.3	Biolegend	147716
CD3	PECy7	17A2	BD Biosciences	560591
CD8	eFluor 450	53-6.7	Biolegend	100725
CD4	BV 650	GK1.5	Biolegend	100404
FOXP3	APC	FJK-16s	Ebioscience	17-5773-82
CD44	BUV737	IM7	BD Biosciences	612799
CTLA4	PECD594	UC10-4F10-11	BD Biosciences	564332
CD69	BV 605	H1.2F3	BD Biosciences	563290

Supplementary Table 5: Marker Gene Lists used for Annotation of scRNA-seq Part 1

**Cell Type**	**Genes**
Zone 3 CTNNB1 WT and MUT (GS+)	*Glul, Axin2, Cyp2e1, Cyp1a2*
Zone 1/2 CTNNB1 MUT (GS+)	*Glul, Hamp, Arg1, Ass1*
Zone 1 CTNNB1 WT (GS-)	*Cyp2f2, Arg1, Ass1*
Reprogrammed hepatocytes	*Prdx2, Prdx5, Hal, Hamp2, Arg1, Ass1, Gstm1*
Endothelial cells	*Tek, Kit, Clec4g, Lyve1*
Erythroid cells	*Hbb-bt, Hba-a1, Hbb-bs*
Hepatic stellate cells	*Ngf, Col1a1, Lrat, Hgf*
Kupffer cells	*Lyz2, Csf1r, Vsig4, Clec4f*

Supplementary Table 6: Marker Gene Lists used for Annotation of scRNA-seq Part 2

Cell Type	Genes
1. T-cells	*Cd2, Cd3d, Cd3e, Cd3g*
1.1 T-cells: CD4 Tn	*Ccr7, Lef1, Tcf7, Il7r, Cd4*
1.2 T-cells CD4 CTL	*Cxcr6, Cxcr3, Cd226, Ctla2, Fasl, Nkg7, Cd40lg, Icos*
1.3 T-cells: CD8 Tn	*Icos, Cd8a, Tcf7, Lef1, Ccr7, Il7r*
1.4 T-cells: CD8 Tex	*Ccl5, Nkg7, Cd8a, Ifngr1, Ctla2a*
1.5 T-cells: Foxp3+ CD4 Treg	*Foxp3, Ctla4, Tnfrsf4, Tox*
1.6 T-cells Foxp1+ CD4 Treg	*Foxp1, Cd4, Ccr7, Il7r, Tox*
2. NK-cells	*Nkg7, Klrd1, Prf1, Cd7, Trdc*
3. Myeloid cells	*Cd68, Lyz1, Lyz2, S100a8, S100a9, Cd14, Aif1, C1qa, C1qb, C1qc, Clec4e, Vsig4*
3.1 Myeloid: M1 macrophage	*S100a8, S100a9, Il1b, Cd14, Lyz2, Itgam, Socs3, Ccl6*
3.2 Myeloid: M2 macrophage	*Clec4f, C1qa, C1qb, C1qc, Vsig4, Folr2, Mrc1, Axl, Msr1, Mertk, Myz2*
3.3 Myeloid: Kupffer cells	*Cd68, Csf1r*
4. B-cells	*Cd79a, Cd19, Ms4a1, Mzb1, Igkc, Iglc2*
5. Dendritic cells	*Fcer1a, Il3ra*
6. Stellate cells	*Rbp1, Col1a1, Col1a2, Dcn*
7. Endothelial cells	*Flt1, Plvap, Lifr, Clec14a, Dnase1l3, Fabp4*
8. Hepatocytes	*Hpd, Fgg, Fga, Pck1, Hamp, Hp*
9. Proliferative cells	*Stmn1, Mki67, Top2a, Cdk1*

Supplementary Table 7: 100 Gene List for Molecular Cartography^{TM} Panel

**Genes**
1. ACKR3
2. ADAMTSL2
3. ADGRE1
4. ANGPT1
5. ANGPT2
6. APC
7. ARG1
8. ARNT
9. ASS1
10. ATP5A1
11. AXIN1
12. AXIN2
13. B2M
14. CCNA1
15. CCNB1
16. CCND1
17. CCNE1
18. CCR4
19. CD4
20. CD68
21. CD8A
22. CDH1
23. CLDN2
24. CLDN7
25. CYP1A2
26. CYP27A1
27. CYP2E1
28. CYP2F2
29. CYP7A1
30. CYP8B1
31. DES
32. EFNB2
33. ELOVL2
34. EPAS1
35. FABP4
36. FADS1
37. FBP1
38. FOXP3
39. GLUL
40. HGF
41. HIF1A
42. HNF4A

43. HSD17B13
44. HTT
45. IGF1
46. IGFALS
47. IGFBP1
48. IGFBP2
49. ITGA2B
50. ITGAM
51. KDR
52. KIT
53. LECT2
54. LGR4
55. LGR5
56. LRAT
57. LYZ2
58. MOGAT1
59. MS4A1
60. NGF
61. OAT
62. PECAM1
63. PIEZO1
64. PON1
65. PTPRC
66. RGN
67. RNF43
68. RSPO1
69. RSPO3
70. SOX9
71. TBX3
72. TEK
73. TERT
74. TIE1
75. TREM1
76. TREM2
77. VTN
78. VWF
79. WLS
80. WNT1
81. WNT10A
82. WNT10B
83. WNT11
84. WNT16
85. WNT2
86. WNT2B

87.	WNT3
88.	WNT3A
89.	WNT4
90.	WNT5A
91.	WNT5B
92.	WNT6
93.	WNT7A
94.	WNT7B
95.	WNT8A
96.	WNT8B
97.	WNT9A
98.	WNT9B
99.	YAP1
100.	ZNRF3

Supplementary Table 8: Marker Gene Lists Used for Molecular Cartography™ Annotation of Cell Types

**Cell Type**	**Genes**
H1: Zone 3 CTNNB1 MUT (GS+)	*Axin2, Glul, Lgr5, Tbx3, Lect2, Cyp1a2*
H2: Zone 3 CV CTNNB1 WT (GS+)	*Glul, Oat, Cyp2e1*
H3: Zone 3 CTNNB1 WT (GS-)	*Cyp2e1, Rgn*
H4: Zone 2/3 CTNNB1 WT (GS-)	*Pon1, Igfbp2, Tert, Fads1, Rgn*
H5: Zone 1 CTNNB1 WT (GS-)	*Elovl2, Ass1, Arg1, Fbp1, Cyp2f2*
H6: Reprogrammed hepatocytes	*Cyp1a2, Cyp8b1, Tert, Atp5a1, Arg1, Cyp2f2*
Endothelial cells	*Vwf, Wnt2, Fabp4, Kit*
Hepatic stellate cells	*Hgf, Lrat, Ngf*
Immune cells	*Adgre1, C4, Itgam, Lyz2, Cd8*

Supplementary Table 9: Marker Gene Lists used for 10X Visium Spatial Transcriptomics Annotation of Cell Types

Cell type	Genes
Tumor markers	*Afp, Ctnnb1, Met, Nfe2l2, Nqo1*
Mutated-β-catenin gene signature (MBGS)	*Axin2, Glul, Lgr5, Nkd1, Notum, Rhbg, Sbspon, Slc1a2, Slc13a3, Sp5, Tcf7, Teddm1, Tnfrsf19*
Proliferation markers	*Stmn1, Mki67, Top2a, Cdk1*
B cell markers	*Cd79a, Cd19, Ms4a1, Mzb1, Iglc2, Igkc, Ighm, Jchain*
T cell markers	*Cd2, Cd3d, Cd3e, Cd3g, Cd4, Cd8a*
NK cells	*Ncam, Klrd1, Prf1, Cd7, Trdc*
Myeloid cell markers	*Cd68, Lyz1, Lyz2, S100a8, S100a9, Cd14, Aif1, C1qa, C1qb, C1qc, Clec4e, Vsig4*
Endothelial cell markers	*Flt1, Plvap, Lifr, Clec14a, Dnase1l3, Fabp4*
Dendritic cell markers	*Fcer1a, Il3ra*
Stellate cell markers	*Rbp1, Col1a1, Col1a2, Dcn*
Hepatocyte markers	*Hpd, Fgg, Fga, Pck1, Hamp, Hp*
Zone 1 hepatocyte markers	*Arg1, Ass1, Cyp2f2, Pck1, Gls2*
Zone 2 hepatocyte markers	*Hamp2, Igfbp2, Pon1, Tert, Cyp8b1*
Zone 3 hepatocyte markers	*Axin2, Cyp1a2, Cyp2e1, Oat, Rgn*

Supplementary Table 10: IRF2 and POU2F1 Target Genes

Gene	Gene set
IRF2	*CD86,RTP4,APP,IFITM1,SAMD9L,NCF4,LST1,FPR1,GIMAP4,IKZF1,FPR2,CFP,IL2RG,IFIT1,ITGAL, IFIT3,IFIT2,CASP1,EPSTI1,EMILIN1,GBP2,CTSC,GBP3,SRGN,P2RY13,CD74,LY86,VAV1,PSMB8, KLF2,PSMB9,HCK,THEMIS2*
POU2F1	*P2RY12,MSR1,COLEC11,RELN,HEXB,LY86,CASP1,IFIT3,CD55,IFIT2*

Supplementary Table 11: Key Resources Table

REAGENT or RESOURCE	SOURCE	IDENTIFIER
Antibodies		
Rabbit polyclonal anti-GS (IHC)	Sigma-Aldrich	Cat#G2781; RRID: AB_259853
Rabbit monoclonal anti-Ki67 (IHC)	Cell Signaling Technology	Cat#12202
Rabbit monoclonal anti-Cyclin D1 (IHC)	Abcam	Cat#ab134175; RRID: AB_2750906
Rabbit monoclonal anti-Myc-tag (IHC)	Cell Signaling Technology	Cat#2278
Mouse monoclonal anti-NQO1 (IHC)	Santa Cruz BioTechnologies	Cat#sc-376023
Mouse monoclonal anti-V5-tag (IHC)	eBioscience™ via Invitrogen	Cat#**14-6796-82**
Rabbit monoclonal anti-CD3 (IHC)	Abcam	Cat#16669
Rabbit polyclonal anti-GZMB (IHC)	Abcam	Cat#4059
Rabbit monoclonal anti-CD4 (IHC)	Abcam	Cat#183685
Rabbit monoclonal anti-CD8 (IHC)	Abcam	Cat#217344
Rabbit monoclonal anti-CD20 (IHC)	Abcam	Cat#236434
Rabbit polyclonal anti-CYP2E1 (IHC)	Sigma-Aldrich	Cat#HPA009128
Mouse monoclonal anti-OAT (IHC)	Santa Cruz BioTechnologies	Cat#sc-376050
Rabbit monoclonal anti-HAMP (IHC)	Abcam	Cat#ab190775
Rabbit polyclonal anti-ARG1 (IHC)	Abcam	Cat#ab91279
Mouse monoclonal anti-CYP2F2 (IHC)	Santa Cruz BioTechnologies	Cat#sc-374540
Rat monoclonal anti-CD45 (IHC)	Santa Cruz BioTechnologies	Cat#sc-53665; RRID: AB_629093
Rat monoclonal anti-CD4 (FACS)	BioLegend	Cat#100404
Rat monoclonal anti-mouse anti-CD3 (FACS)	BD Biosciences	Cat#560591
Rat monoclonal anti-mouse anti-CD8 (FACS)	BioLegend	Cat#100725 RRID: AB_493426
Rat monoclonal anti-FOXP3 (FACS)	eBioscience™ via Invitrogen	Cat#17-5773-82
Rat monoclonal anti-CD44 (FACS)	BD Biosciences	Cat#612799
Hamster monoclonal anti-CTLA4/CD152 (FACS)	BD Biosciences	Cat#564332
Hamster monoclonal anti-CD69 (FACS)	BD Biosciences	Cat#563290
Rat monoclonal anti-CD45 (FACS)	BioLegend	Cat#147716 RRID: AB_2750449
Biotinylated secondary antibody, Rabbit (IHC)	Millipore Sigma	Cat#AP182BMI
Biotinylated secondary antibody, Mouse (IHC)	Millipore Sigma	Cat#AP181B
Bacterial and virus strains		
NA	NA	NA
Biological samples		
NA	NA	NA
Chemicals, peptides, and recombinant proteins		
Formalin	Fisher Chemicals	Cat#SF100-4
Hematoxylin	Fisher Chemicals	Cat#SH26-500D
Eosin	Eosin Y	Cat#23-314-63

DAKO reagent	Agilent Technologies	Cat#S236784-2
H_2O_2 solution	Fisher Chemicals	Cat#H327-500
Super Block	ScyTek Laboratories	Cat#AAA125
PBS	Fisher	Cat#PI28374
Mouse IFNγ	Miltenyi Biotec	Cat#130-105-785
InVivoMAb anti-mouse CD3	BioXCell	Cat#BE0002
Critical commercial assays		
TRIzol™	Thermo Scientific	Cat#15596026
RNeasy Mini Kit	Qiagen	Cat#74104
Power SYBR® Green PCR Master Mix	Applied Biosystems	Cat#4367660
VECTASTAIN® Elite® ABC-HRP Kit, Peroxidase	Vector Laboratories	Cat#PK-6101
VECTASTAIN® Elite® ABC-AP Kit, Alkaline Phosphatase	Vector Laboratories	Cat#AK-5000
DAB Substrate Kit, Peroxidase (HRP)	Vector Laboratories	Cat#SK-4100
Vector® Red Substrate Kit, Alkaline Phosphatase (AP)	Vector Laboratories	Cat#SK-5100
Endotoxin-Free Maxiprep kit	Sigma-Aldrich	Cat#NA0410
ApopTag Peroxidase In Situ Apoptosis Detection Kit	Millipore Sigma	Cat#S7100
TotalSeq™-C Mouse Universal Cocktail	BioLegend	Cat#199903
Deposited data		
Raw transcriptomic data (β-N and β-M models treated with LNP-CTRL and LNP-CTNNB1; n=3-4 each condition)	This paper	GSE270414
Raw transcriptomic data (2 β-M-pT3and 4 β-M-IRF2 livers)	This paper	GSE270415
Raw single-cell RNA-seq data (β-N model treated with LNP-CTRL and LNP-CTNNB1) – single-cell GEX	This paper	GSE270714
Raw single-cell RNA-seq data (β-N model treated with LNP-CTRL and LNP-CTNNB1) – single-cell with immune profiling	This paper	GSE270974
Raw single-cell spatial data via Molecular Cartography™ (β-N model treated with LNP-CTRL and LNP-CTNNB1)	This paper	GSE270708
Raw 10X Visium spatial data (β-M model treated with LNP-CTRL and LNP-CTNNB1)	This paper	GSE270975
Raw transcriptomic data (β-catenin-mutated HCC mouse models)	Ruiz de Galarreta et al. 2019[1]	GSE125336
Raw transcriptomic data (β-catenin knockout mouse livers)	Lempiainen et al. 2011[2] & Luisier et al. 2014[3]	GSE68779
Single-cell human liver atlas	Guilliams et al. 2022[4]	GSE192742
Single-cell mouse liver atlas	Guilliams et al. 2022[4]	GSE192742
TCGA-LIHC (374 Hepatocellular Carcinoma whole exome and transcriptomic data)	The Cancer Genome Atlas Research Network et al. 2017[5]	https://portal.gdc.cancer.gov/projects/TCGA-LIHC
Experimental models: Cell lines		
NA	NA	NA
Experimental models: Organisms/strains		
Mouse: FVB/NJ	The Jackson Laboratories	Strain #:001800 RRID:IMSR JAX:001800
Mouse: Ctnnb1$^{f/f}$ (C57BL/6J background)	The Jackson Laboratories	Strain #:004152 RRID:IMSR JAX:004152
Oligonucleotides		

Primers for qPCR	This paper	Table SX
Probes for mouse versus human CTNNB1	This paper	Table SX
Probes for Molecular Cartography	This paper	Table SX
Recombinant DNA		
pT3-EF5αh-S45Y-hCTNNB1-Myc-tag	Tao et al. 2016[6]	https://doi.org/10.1002/hep.28601
pT3-EF1αh-T41A-hCTNNB1-Myc-tag	Tao et al. 2021[7]	https://doi.org/10.1002/hep.31730
pT3-EF5αh-hMet-V5-tag	Tao et al. 2016[6]	https://doi.org/10.1002/hep.28601
pT3-EF1αh-G31A-hNFE2L2	Tao et al. 2021[7]	https://doi.org/10.1002/hep.31730
pT3-EF1α-HA-myr-AKT	Ho et al. 2012[8]	https://doi.org/10.1002/hep.24736
pT2-Caggs-N-RasV12	Ho et al. 2012[8]	https://doi.org/10.1002/hep.24736
pT3-EF1αh-Irf2	This paper	pFUW-tetO-Irf2 plasmid from Addgene (plasmid #139814)
pT3-EF1αh-POU2F1	This paper	pENTR223-POU2F1 plasmid from DNASU (plasmid #HsCD00505520)
pCMV-empty vector (pCMV-pT3)	Zhang et al. 2021[9]	https://doi.org/10.1016%2Fj.ajpath.2021.01.010
pCMV-Sleeping Beauty transposase (pCMV-SB)	Tao et al. 2016[6]	https://doi.org/10.1002/hep.28601
pCMV-Cre	Zhang et al. 2021[9]	https://doi.org/10.1016%2Fj.ajpath.2021.01.010
Software and algorithms		
ImageJ	Benhamouche et al. 2006[10]	https://imagej.nih.gov/ij/
QuPathv0.5.1	Bankhead et al. 2017[11]	https://qupath.readthedocs.io/en/stable/
Seuratv4	Butler et al. 2018[12]	https://doi.org/10.1016/j.cell.2021.04.048
Other		
NA	NA	NA

Resource availability

Lead contact: Further information and requests for resources, data, and reagents should be directed to and will be fulfilled by the lead contact, Satdarshan P. Monga (smonga@pitt.edu).

Materials availability: Reagents used in this study are available from the lead contact with a completed Material Transfer Agreement
