# Supplementary material for: Precision targeting of β-catenin induces tumor reprogramming and immunity in hepatocellular cancers
Source: Nat Commun. 2025 May 30;16:5009. doi: 10.1038/s41467-025-60457-2 (PMC12122713; doi:10.1038/s41467-025-60457-2)
Supplement: Supplementary file 2 — Reporting Summary [file 41467_2025_60457_MOESM2_ESM.pdf]

nature portfolio

Corresponding author(s): Satdarshan P. Monga, MD

Last updated by author(s): May 7, 2025

Reporting Summary

Nature Portfolio wishes to improve the reproducibility of the work that we publish. This form provides structure for consistency and transparency in reporting. For further information on Nature Portfolio policies, see our Editorial Policies and the Editorial Policy Checklist.

Statistics

For all statistical analyses, confirm that the following items are present in the figure legend, table legend, main text, or Methods section.

n/a	Confirmed	
☐	☒	The exact sample size (*n*) for each experimental group/condition, given as a discrete number and unit of measurement
☐	☒	A statement on whether measurements were taken from distinct samples or whether the same sample was measured repeatedly
☐	☒	The statistical test(s) used AND whether they are one- or two-sided<br>*Only common tests should be described solely by name; describe more complex techniques in the Methods section.*
☒	☐	A description of all covariates tested
☐	☒	A description of any assumptions or corrections, such as tests of normality and adjustment for multiple comparisons
☐	☒	A full description of the statistical parameters including central tendency (e.g. means) or other basic estimates (e.g. regression coefficient) AND variation (e.g. standard deviation) or associated estimates of uncertainty (e.g. confidence intervals)
☐	☒	For null hypothesis testing, the test statistic (e.g. *F*, *t*, *r*) with confidence intervals, effect sizes, degrees of freedom and *P* value noted<br>*Give P values as exact values whenever suitable.*
☒	☐	For Bayesian analysis, information on the choice of priors and Markov chain Monte Carlo settings
☒	☐	For hierarchical and complex designs, identification of the appropriate level for tests and full reporting of outcomes
☒	☐	Estimates of effect sizes (e.g. Cohen's *d*, Pearson's *r*), indicating how they were calculated

Our web collection on statistics for biologists contains articles on many of the points above.

Software and code

Policy information about availability of computer code

Data collection	All analysis was performed using R (Version 4.3.1)
Data analysis	Seurat v4 was utilized for single-cell RNA-seq and spatial transcriptomic analyses<br>No custom code was generated, standard pipelines available from Seurat vignettes were utilized

For manuscripts utilizing custom algorithms or software that are central to the research but not yet described in published literature, software must be made available to editors and reviewers. We strongly encourage code deposition in a community repository (e.g. GitHub). See the Nature Portfolio guidelines for submitting code & software for further information.

Data

Policy information about availability of data

All manuscripts must include a data availability statement. This statement should provide the following information, where applicable:

- Accession codes, unique identifiers, or web links for publicly available datasets
- A description of any restrictions on data availability
- For clinical datasets or third party data, please ensure that the statement adheres to our policy

For high-throughput multi-omics data, all the raw and processed files are uploaded to Gene Expression Omnibus (GEO) with accession ID GSE270977 (https://www.ncbi.nlm.nih.gov/geo/query/acc.cgi?acc=GSE270977). The bulk RNA-seq CTNNB1/NFE2L2 data, CTNNB1/hMet/NFE2L2 data, CTNNB1/MET/IRF2 data, and CTNNB1/NRF2/POU2F1 data can be downloaded by GSE290449, GSE270414, GSE270415, and GSE290444, respectively; the single-cell spatial transcriptomics data

by Resolve Biosciences Molecular Cartography can be accessed by GSE270708; the single-cell RNA-seq GEX and the single-cell coupled with hashtag immune profiling data can be downloaded by GSE270714 and GSE270974, respectively; and, the spatial transcriptomics data by 10X Visium platform can accessed by GSE270975 (-M model) and GSE290445 (-N model). All clinical, raw RNA-seq and WES data for the IMbrave150 trial are deposited in the European Genome-Phenome Archive under accession no. EGAS00001005503. Qualified researchers may request access to individual patient-level data through the clinical study data request platform (https://vivli.org/). The remaining data are available within the Source Data file. All key resources used are provided in Supplementary Table 11.

Research involving human participants, their data, or biological material

Policy information about studies with human participants or human data. See also policy information about sex, gender (identity/presentation), and sexual orientation and race, ethnicity and racism.

Reporting on sex and gender	N/A
Reporting on race, ethnicity, or other socially relevant groupings	N/A
Population characteristics	N/A
Recruitment	N/A
Ethics oversight	N/A<br>N/A; human data utilized in the study was retrospective in nature, deidentified and did not require IRB oversight. This was also reported in previous Nature Medicine paper (Genentech collaboration). PMID: 35739268.

Note that full information on the approval of the study protocol must also be provided in the manuscript.

Field-specific reporting

Please select the one below that is the best fit for your research. If you are not sure, read the appropriate sections before making your selection.

☒ Life sciences ☐ Behavioural & social sciences ☐ Ecological, evolutionary & environmental sciences

For a reference copy of the document with all sections, see nature.com/documents/nr-reporting-summary-flat.pdf

Life sciences study design

All studies must disclose on these points even when the disclosure is negative.

Sample size	Sample size calculations were made using GPower3 with a a=0.05, b=0.8, and µ=2.5. Overall survival among the treatment groups was assessed with Kaplan-Meier curve and log-rank test. Statistical analysis was performed using Prism 9 for Mac OS X software (Version 9) (GraphPad Software, Inc.).
Data exclusions	No data was excluded from the study.
Replication	All experimental results from sequencing were validated by traditional molecular biology techniques. Results were replicated in multiple models utilized. Immunohistochemistry was performed at least twice and in multiple replicate animals.
Randomization	Animals were all housed in same conditions. 3-4 mice were housed in a cage at a time. Each cage was designated a different treatment group.
Blinding	Blinding during the study was not performed as many experiments required knowledge of treatment groups for allocation to subsequent treatments or for relevant downstream analyses. In analyses requiring unbiased analyses, blinding would hinder the ability to identify meaningful results.

Reporting for specific materials, systems and methods

We require information from authors about some types of materials, experimental systems and methods used in many studies. Here, indicate whether each material, system or method listed is relevant to your study. If you are not sure if a list item applies to your research, read the appropriate section before selecting a response.

Materials & experimental systems

n/a	Involved in the study	
☐	☒	Antibodies
☒	☐	Eukaryotic cell lines
☒	☐	Palaeontology and archaeology
☐	☒	Animals and other organisms
☐	☒	Clinical data
☒	☐	Dual use research of concern
☒	☐	Plants

Methods

n/a	Involved in the study	
☒	☐	ChIP-seq
☐	☒	Flow cytometry
☒	☐	MRI-based neuroimaging

Antibodies

Antibodies used	Please see Supplementary Table 1 in the Online Supplement for more Details.<br>MYC-tag 2278 Cell Signaling RbM (1:100); clone 71D10<br>NQO1 376023 Santa Cruz MsM (1:100); clone H-9<br>V5-tag 14-6796-82 eBioscience MsM (1:100); clone TCM5<br>Glutamine synthetase (GS) G2781 Sigma-Aldrich RbP(1:1500); clone G2781<br>Ki67 12202 Cell Signaling RbM (1:500); clone D3B5<br>Cyclin-D1 Ab134175 Abcam RbM (1:100); clone EPR2241<br>CYP2E1 HPA-009128 Sigma RbP (1:100); polyclonal<br>OAT 376050 Santa Cruz MsM (1:100); clone D10<br>HAMP 190775 Abcam RbM (1:100); clone EPR18937<br>ARG1 91279 Abcam RbP(1:100); polyclonal<br>CYP2F2 374540 Santa Cruz MsM (1:100); clone F-9<br>CD3 Ab16669 Abcam RbM (1:100); clone SP7<br>CD4 Ab183685 Abcam RbM (1:100); clone EPR19514<br>CD8 Ab217344 Abcam RbM (1:100); clone EPR21769<br>CD20 Ab236434 Abcam RbM (1:100); clone SP32<br>S100A8/9 Ab288715 Abcam RbM (1:100); clone RM1038<br>CD45 53665 Santa Cruz RtM (1:100); clone 30-F11<br>GZMB Ab4059 Abcam RbP (1:100); clone polyclonal
Validation	All antibodies were commerically available and validated by respective companies. Additionally, all antibodies have been previously published by us. We have utilized all pre-validated antibodies based on the manufacturer website, we also run positive and negative controls based on expected distribution.

Animals and other research organisms

Policy information about studies involving animals; ARRIVE guidelines recommended for reporting animal research, and Sex and Gender in Research

Laboratory animals	FVB/NJ male mice 6-8 weeks of age from Jackson Labs; the number of mice is indicated in each legend. Animals are housed in 12h light/dark cages in NIH funded DLAR supported IACUC approved facility under ACUC approved protocol.
Wild animals	N/A
Reporting on sex	We utilized male mice in this study. Liver cancer occurs in a 2:1 ratio in males:females.
Field-collected samples	N/A
Ethics oversight	University of Pittsburgh IACUC; protocol # 22112055.

Note that full information on the approval of the study protocol must also be provided in the manuscript.

Clinical data

Policy information about clinical studies
All manuscripts should comply with the ICMJEguidelines for publication of clinical research and a completedCONSORT checklist must be included with all submissions.

Clinical trial registration	N/A
Study protocol	N/A; this was retrospective analysis of previously published work performed by Genentech and previously published in Nature Medicine paper (PMID: 35739268)
Data collection	N/A

Outcomes	Overall and progression-free survival, CTNNB1 mutational status, tertiary lymphoid structure presence on H&E, and B/T cell signature

Plants

Seed stocks	N/A
Novel plant genotypes	N/A
Authentication	N/A

Flow Cytometry

Plots

Confirm that:

- [x] The axis labels state the marker and fluorochrome used (e.g. CD4-FITC).
- [x] The axis scales are clearly visible. Include numbers along axes only for bottom left plot of group (a 'group' is an analysis of identical markers).
- [x] All plots are contour plots with outliers or pseudocolor plots.
- [x] A numerical value for number of cells or percentage (with statistics) is provided.

Methodology

Sample preparation	Hepatic lymphocytes and monocytes were enriched from the nonparenchymal fraction using a Percoll gradient combined with centrifugation. Briefly, the nonparenchymal fraction from liver perfusions were centrifuged (2000rpm for 10 minutes) followed by 36% Percoll separation in Roswell Park Memorial Institute 1640 medium supplemented with + 5% FBS. Erythrocytes were removed using Ammonium-Chloride-Potassium Lysing Buffer (ThermoFisher Scientific). Isolated cells were counted for viability, which was typically >90%, using trypan blue.
Instrument	Cytek Aurora (Cytek Biosciences, Bethesda, MD)
Software	FlowJo software v10.1 (Treestar, Ashland, OR)
Cell population abundance	No cell sorting was performed.
Gating strategy	Gating strategy provided in supplemental file. SSC-A/FSC-A were initial gates, the indicated cell populations in percent are detailed there on the figure.

- [x] Tick this box to confirm that a figure exemplifying the gating strategy is provided in the Supplementary Information.
